# Initiator and elongator tRNA recognition mechanism in *Mycobacterium tuberculosis* methionyl-tRNA synthetase

**DOI:** 10.1016/j.jbc.2025.110939

**Published:** 2025-11-14

**Authors:** Shivani Thakur, Rukmankesh Mehra

**Affiliations:** 1 Department of Chemistry, Indian Institute of Technology Bhilai, Durg, Chhattisgarh, India; 2 Department of Bioscience and Biomedical Engineering, Indian Institute of Technology Bhilai, Durg, Chhattisgarh, India

**Keywords:** aminoacyl tRNA synthetase, elongator tRNA, enzyme mechanism, initiator tRNA, methionyl-tRNA synthetase, molecular dynamics, *Mycobacterium tuberculosis*, transfer RNA

## Abstract

Protein synthesis is an essential target for anti-tubercular drug design. Methionyl-tRNA synthetase (MetRS) plays a crucial role in both the initiation and elongation phases of protein synthesis. Molecular recognition of the CAU anticodon of tRNA by MetRS during these two processes is a key step. To date, no experimental structures from *Mycobacterium tuberculosis* (*Mtb*) have revealed this binding. Therefore, we modeled the *Mtb* MetRS complexes with initiator and elongator tRNAs to find their differential binding mechanisms during molecular dynamics simulations of 9 microseconds. We found that the elongator tRNA formed a stable complex with the protein, whereas the initiator tRNA bound transiently. Nevertheless, major intra-tRNA interactions were maintained in both. This may reflect the rapid charging of the initiator tRNA, in contrast to the elongator tRNA, which may require more time for aminoacylation. tRNA interacts with both the active site and the anticodon-binding domain. Electrostatic attractions between tRNA and the protein’s catalytic domain likely facilitate its charging with methionine. Meanwhile, a combination of repulsive and attractive forces between the tRNA and the protein’s connective peptide domain and KMSKS loop triggered opening of the binding pocket, promoting the reaction and subsequent product release. Concurrently, strong tRNA binding to the anticodon domain supported this process. These findings suggest a possible pathway of tRNA charging. The tRNA formed salt-bridges with positively charged Arg and Lys, whereas negatively charged Asp and Glu caused repulsive binding. In brief, this study provides a plausible mechanism for the differential recognition of initiator and elongator tRNA by *Mtb* MetRS.

Tuberculosis is a global epidemic caused by *Mycobacterium tuberculosis* (https://www.who.int/teams/global-programme-on-tuberculosis-and-lung-health/tb-reports/global-tuberculosis-report-2024). The emergence of drug resistance is challenging current therapies and driving ongoing efforts to explore new drug targets (1, 2, 3, 4, 5, 6, 7, 8, 9, 10, 11, 12). Protein synthesis is a crucial target in the development of new anti-tubercular agents. Aminoacyl-tRNA synthetases (aaRSs) catalyze the attachment of specific amino acids to their cognate tRNAs, which subsequently deliver the amino acids to the ribosome for protein synthesis. Methionine initiates translation and is also incorporated during elongation of the polypeptide chain. This makes methionyl-tRNA synthetase (MetRS), the enzyme responsible for methionine charging, both a key drug target (13, 14) and central to research on the origin of life (15, 16, 17).

Methionyl-tRNA synthetase (MetRS) charges two types of tRNAs, initiator and elongator, associated with the initiation and elongation phases of translation, respectively (Fig. 1, *A* and *B*). *M. tuberculosis* (*Mtb*) possesses three genes that encode tRNAs with the CAU anticodon: *metU* for initiator tRNA, and *metT* and *metV* for elongator tRNA (Fig. S1 and Supplementary Section S1). *metV* undergoes post-transcriptional modifications and is charged with isoleucine instead of methionine (14, 18). The initiator and elongator tRNAs (each 77 nucleotides in length) corresponding to *metU* and *metT* participate in binding to MetRS (13, 14).

**Figure 1:**
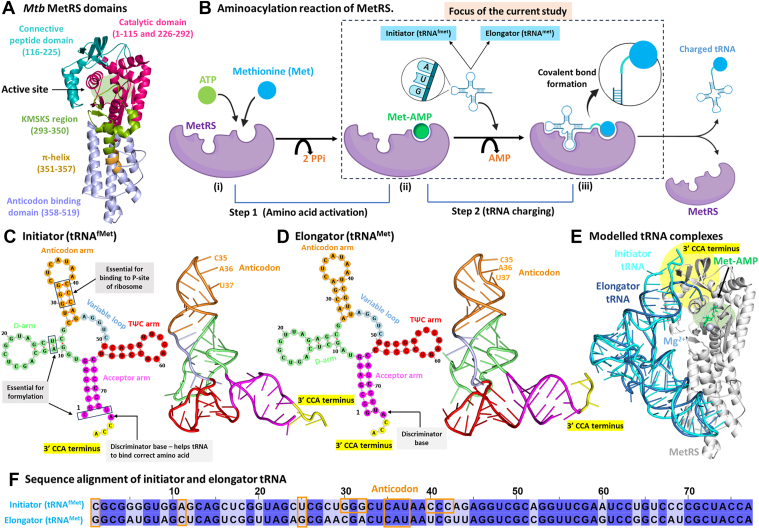
**MetRS and tRNA in the aminoacylation reaction.***A*, *Mtb* MetRS structure showing the catalytic domain (pink, 1–115 and 226–292), connective peptide (CP) domain (*dark cyan*, 116–225), KMSKS domain (*green*, 293–350), π-helix region (brown, 351–357) and anticodon domain (*light blue*, 358–519). *B*, steps involved in the aminoacylation reaction. Cloverleaf and L-shaped model of (*C*) the initiator and (*D*) the elongator tRNA from *Mtb*. *E*, superimposed structures of the modeled protein-tRNA complexes (initiator and elongator). *F*, nucleotide sequence alignment of both tRNAs, highlighting distinct nucleotides and the conserved anticodon (*orange*). For consistency, nucleotides are numbered sequentially from 1 to 77 (may differ from the literature).

MetRS catalyzes a two-step reaction (Fig. 1*B*) (19, 20). In the first step, the substrates methionine and ATP bind to the enzyme and form the methionyl-AMP (Met-AMP) intermediate. Subsequently, the tRNA binds to MetRS *via* its anticodon and 3′ CCA (acceptor arm) at two distinct binding sites, resulting in the charging of the tRNA with methionine, which is then released from the enzyme (19, 20, 21).

The *Mtb* MetRS consists of 519 amino acids and is structurally divided into five main regions: the catalytic domain (residues 1–115 and 226–292), the connective peptide (CP) domain (116–225), the KMSKS loop region (293–350), the π-helix region (351–357), and the anticodon binding domain (ABD; 358–519) (Fig. 1*A*) (22). Aminoacyl-tRNA synthetases are classified into two structural classes: class I and II (23). *Mtb* MetRS belongs to class I, which is characterized by a Rossmann fold in the catalytic domain and two signature motifs in the active site, namely, HIGH (in *Mtb*: _18_HVGH_21_) and _299_KMSKS_303_. Class I enzymes interact with the minor groove of the tRNA acceptor stem and transfer the amino acid to the 2′-OH of the ribose at the 3′ terminal adenosine (24). In contrast, class II enzymes attach the amino acid to the 3′-OH of the terminal adenosine on the tRNA. They comprise a unique α/β fold and three conserved motifs (23).

The initiator and elongator methionine tRNAs of *Mtb* adopt a typical cloverleaf structure (Fig. 1, *C* and *D*), each consisting of 77 nucleotides. The structure is divided into the acceptor arm (nucleotides 1–7 and 67–77), D-arm (8–27), anticodon arm (28–44), variable loop (45–49), and TΨC arm (50–66) (25, 26, 27). Three highly conserved features of eubacterial initiator tRNA (also in *Mtb*) include unpaired bases at positions C1:U73, three consecutive G:C base pairs between G30 to 32 and C40 to 42 in the acceptor stem, and an A11:U25 base pair in the D-arm. These features are absent in elongator tRNA (28).

The substrates (methionine and ATP) and the 3′ CCA terminus of the tRNA bind to the catalytic domain, CP domain, and KMSKS region of the protein (22, 29, 30). The tRNA anticodon arm also interacts with the ABD site of MetRS, enabling recognition of the CAU anticodon by the enzyme. These interactions at two distinct protein sites facilitate the tRNA charging. The initiator and elongator tRNAs share 67.9% sequence identity (Fig. 1*F*, Table S1), suggesting the possibility of differential recognition mechanisms and functional consequences.

Molecular dynamics (MD) simulations of MetRS have previously been performed on homologs from *Escherichia coli* (29, 31, 32, 33), *Leishmania infantum* (34), *Proteus mirabilis* (35), and *Mtb* (20, 21, 36, 37). In the present study, we performed a detailed study of the molecular recognition of initiator *versus* elongator tRNA by *Mtb* MetRS using MD simulations. We modeled the three-dimensional (3D) states of these complexes and identified structure-function relations among the tRNA, protein, and Met-AMP. These relations helped elucidate distinct structural and binding features of the initiator and elongator tRNA complexes, as well as plausible mechanisms of tRNA recognition.

## Results and discussion

### The protein in the elongator state exhibited higher conformational dynamics

**Figure 2:**
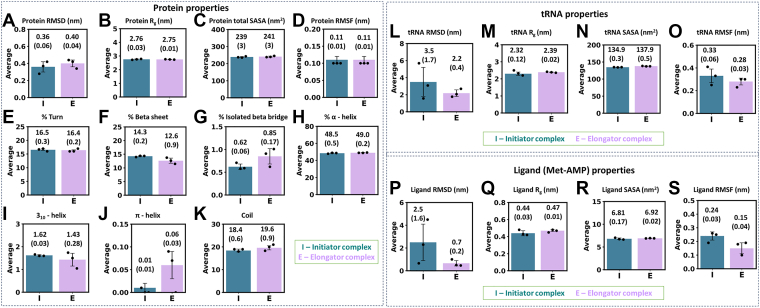
**Comparisons of simulated average properties and standard deviations (value in bracket; error bar) from three independent MD runs for initiator (I; teal) *versus* elongator (E; light purple) complexes.** Protein properties: (*A*) RMSD, (*B*) R_g_, (*C*) total SASA, (*D*) RMSF, and (*E*–*K*) secondary structures. tRNA properties: (*L*) RMSD, (*M*) R_g_, (*N*) SASA, and (*O*) RMSF. Ligand (Met-AMP) properties: (*P*) RMSD, (*Q*) R_g_, (*R*) total SASA, and (*S*) RMSF. Further, the statistically significant differences between the properties of the initiator and elongator complexes were analyzed using block averaging strategies and by performing t-tests (*p*-values) on the block-averaged data, as discussed in Supplementary Section S2 and shown in Tables S3 and S4.

We prepared two *Mtb* tRNA-MetRS complexes: one with initiator tRNA (tRNA^fMet^) and the other with elongator tRNA (tRNA^Met^) (Figs. 1*E*, S2). In both complexes, intermediate (Met-AMP) and Mg^2+^ were present. MD simulations of both complexes were performed in triplicate for 1000 ns each, for a total of 6 microseconds (μs; 2 systems × 3 μs each). Primary analysis was carried out on the full trajectory, involving RMSD, R_g_, and SASA. The protein backbone RMSD showed greater conformational flexibility in the elongator protein compared to the initiator (Fig. S3*A*). The corresponding average RMSD values across the three MD replicates were 0.40 ± 0.04 nm and 0.36 ± 0.06 nm (Fig. 2*A*).

Despite some structural fluctuations, the protein’s R_g_ stayed consistent in both complexes (Fig. S3*B*). This suggests that the overall size and compactness of the protein were preserved throughout the simulations (Fig. 2*B*). The solvent-accessible surface area of MetRS in the initiator (239 ± 3 nm^2^) and elongator (241 ± 3 nm^2^) states remained similar (Figs. 2*C* and S3*C*). In line with this, the hydrophobic and hydrophilic SASA of protein in both initiator and elongator complexes were also similar (Fig. S4), indicating that the conformational changes did not disturb the solvent exposure of the protein surface residues.

### Dominant motions observed in the N-terminal, CP domain, KMSKS loop, and C-terminal

Free energy landscape principal component analysis (FEL-PCA) was performed to investigate the dominant motions of the protein in the initiator and elongator tRNA complexes. The first two eigenvectors (EV1 and EV2) accounted for a significant portion of the total motion (details in Supplementary Section S3 and Fig. S5). The porcupine plots of EV1 and EV2 for six simulations revealed the conformationally active protein regions (Figs. S6 and S7). Dominant motions were observed in key functional regions, including the catalytic domain (residues 9–21), the CP domain (116–155), the KMSKS loop (296–306), and the C-terminal, suggesting enhanced mobility around the active site.

Residue-wise root-mean-square-fluctuation (RMSF) (Figs. 2*D* and S8) and the ratio of intra-molecular contacts (Fig. S9) of protein further supported the observations from the porcupine plots. The N- and C-terminals, CP domain, and KMSKS loop consistently exhibited higher flexibility and more intra-molecular contacts. Notably, the CP domain displayed increased RMSF and a higher number of intramolecular contacts in the elongator complex than in the initiator (Fig. S9*C*). This might be due to the conformational changes driven by prominent interactions of the CP domain with the elongator tRNA. In contrast, the KMSKS loop (299–303) exhibited comparatively greater flexibility and contacts in the initiator complex. This indicates that tRNA interactions at this region may induce opening of the KMSKS loop around the active site.

### Decrease in β-sheet content found in elongator protein

Analysis of the protein secondary structures over the last 500 ns of the simulations revealed distinct patterns between the initiator and the elongator states (Fig. 2, *E*–*K* and trajectories in Fig. S10). Specifically, the β-sheet content was higher in the initiator complex (14.3 ± 0.2%) compared to the elongator (12.6 ± 0.9%). These findings indicate the formation of comparatively more stable structural elements in the initiator protein. Minor differences were observed in other secondary structures. The percentage of isolated β-bridge was marginally lower in the initiator protein, whereas the π-helix content was elevated in the elongator.

Notably, residues 120 to 125 in the CP domain formed a regular β-sheet in the initiator protein, whereas this region majorly converted to a flexible coil structure in the elongator complex (indicated by dashed line, Fig. S11). Thus, the initiator complex exhibited a higher proportion of β-sheets, contributing to the comparatively greater stability of the protein, with the prominent differences localized to the CP domain (120–125). The CP domain directly binds with tRNA *via* its CP1 knuckle region (residue 120–140) (14, 29, 38). As noted above, this domain showed substantial fluctuations and formed numerous intra-molecular contacts in both initiator and elongator states, with higher fluctuations and more contacts in the elongator protein. These results suggest that prolonged binding of the elongator tRNA to the CP domain promotes the transition of a stable β-sheet into a coil structure, possibly due to persistent repulsive tRNA-protein interactions in this region (discussed later). In contrast, such repulsive interactions in the initiator complex likely occurred over a shorter duration, thereby stabilizing the β-sheet structure.

### Stable binding of elongator tRNA and transient binding of initiator tRNA

**Figure 3:**
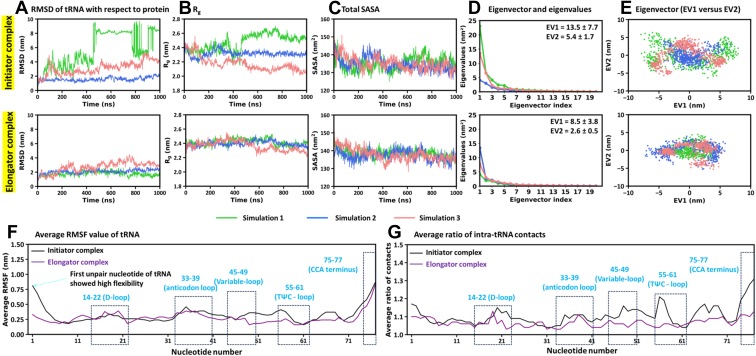
**Comparisons of simulated properties of initiator and elongator tRNAs.***A*, RMSD. *B*, R_g_. *C*, Total SASA. *D*, Eigenvectors *versus* eigenvalues plots. Average and standard deviation of eigenvalues for EV1 and EV2 over the three simulations are shown. *E*, 2D projections along EV1 and EV2. *F*, average RMSF values from three runs, showing flexibility of nucleotides. *G*, average ratio of intra-tRNA contacts of each nucleotide. The contact ratio is the total number of contacts divided by their mean value over the simulation.

Primary analysis of tRNA dynamics over the full trajectory revealed greater conformational flexibility of the initiator tRNA compared to the elongator (Figs. 2*L* and 3*A*). The average RMSD (across three replicates) was 3.5 ± 1.7 nm for the initiator tRNA, *versus* 2.2 ± 0.4 nm for the elongator. This suggests that the elongator tRNA maintained a more stable conformation, while the initiator tRNA formed transient interactions with the protein (Figs. S12–S18). Interestingly, one of the simulations of the initiator complexes (in green) showed significantly higher tRNA movement (Fig. 3*A*). In this simulation, the tRNA moved from its initial position to multiple sites on the protein (Fig. S13). Specifically, the 3′ CCA end shifted from the front of the active site (toward the KMSKS loop and CP domain; Fig. S12*A*) to the opposite side of MetRS, where it interacted with the catalytic domain. Figure S13 illustrates this phenomenon with structures from every 100 ns frame. In this case, the tRNA (green in Fig. 3*B*) adopted a more extended conformation compared to the other two runs (red and blue).

Although the average R_g_ of the initiator tRNA (2.32 ± 0.12 nm) was similar to the elongator (2.39 ± 0.02 nm, Fig. 2*M*), the initiator tRNA exhibited a higher variability (Fig. 3*B*). However, the average SASA of the initiator tRNA was lower (134.9 ± 0.3 nm^2^) than the elongator (137.9 ± 0.5 nm^2^; Figs. 2*N* and 3*C*). These findings indicate that the initiator tRNA binds transiently, with a higher variability in size. The elongator tRNA maintains a more stable and slightly extended conformation during simulations.

To confirm the differential motion of initiator *versus* elongator tRNA, FEL-PCA was performed. This analysis showed greater motion and a broader conformational landscape for the initiator tRNA (Fig. 3, *D* and *E*). The average EV1 and EV2 values (of three runs) were 13.5 ± 7.7 and 5.4 ± 1.7 nm^2^ for the initiator tRNA, respectively, compared to 8.5 ± 3.8 and 2.6 ± 0.5 nm^2^ for the elongator tRNA (Fig. 3*D*). Scatter plots of EV1 *versus* EV2 further illustrated a wider conformational space sampled by the initiator tRNA (Fig. 3*E*), whereas the elongator tRNA spanned a comparatively confined space, reflecting its reduced motion.

RMSF analysis of nucleotides showed that the initiator tRNA typically displayed higher fluctuations (Figs. 2*O*, 3*F*, and S19), which could relate to its higher ratio of intramolecular contacts (Figs. 3*G* and S20) compared to the elongator tRNA. The contact ratio represents an increase in intramolecular contacts, defined as the ratio of the number of atomic contacts of a residue to the mean contacts within 1.5 nm distance. This distance cutoff caused the fluctuating residues to move in and out of contact frequently, thereby increasing the contact count. Flexible regions, including loops, formed many transient contacts because they swept across a wide region and interacted with many different atoms. Consequently, transient contacts of mobile regions increased the contact count while also exhibiting high RMSF. In contrast, stable contacts were associated with reduced RMSF. Therefore, higher RMSF regions were related to more dynamic contacts.

The CCA end (nucleotides 75–77) showed increased dynamics in both initiator and elongator tRNAs. The first nucleotide (C1) of the initiator tRNA showed elevated flexibility. In addition, a higher contact ratio was observed for nucleotides in the TΨC (55–61) and variable loops (45–49) of the initiator tRNA (Fig. 3*G*). In one initiator complex simulation (green), the TΨC loop (nucleotides 55–61) exhibited comparatively higher fluctuations (Fig. S19*A*) and contact ratio (Fig. S20*A*) compared to the other two runs (blue and red). This region interacted with the catalytic domain (residues 243–248) of MetRS (Fig. S6*A*) and induced its higher movement, which was absent in the other two initiator runs (blue and red; Fig. S6, *B* and *C*).

These results lead to the concept that the initiator tRNA in *Mtb* may bind for a shorter time and perform aminoacylation reaction more rapidly, which could be due to its necessity to initiate protein synthesis. However, the elongator tRNA remained stably bound to MetRS and may require a longer residence time for charging, consistent with its role in translation elongation. The respective short and long lifetimes of the initiator and elongator tRNA complexes, respectively, perfectly correlated with their experimental association constant (K_a_) values of 16 ± 2 and 57 ± 10 μM^-1^ in *Escherichia coli* (*E. coli*) (39). A lower K_a_ indicates a shorter complex lifetime. The quick reaction also possibly relates to the rapid formylation of initiator tRNAs, which is essential for their preferential binding to the ribosome’s P-site and exclusion of other tRNAs (40).

### Ligand spanned a shorter period in the active site of the initiator complex

Primary analysis of the ligand over 1000 ns across three simulation replicates revealed a highly unstable nature of Met-AMP in the initiator complex relative to the elongator (Figs. 2*P* and S21*A*). In the initiator complex, Met-AMP remained in the protein’s binding pocket for a shorter duration, whereas in the elongator complex, the ligand occupied the active site throughout the simulations (Fig. S21*A*). The average RMSD of Met-AMP across the three runs was 2.5 ± 1.6 nm in the initiator complex and 0.7 ± 0.2 nm in the elongator complex (Fig. 2*P*).

The higher ligand motion in the initiator complex was supported by increased variability in its R_g_ in initiator complexes (Fig. S21*B*). In contrast, the ligand’s R_g_ remained stable in the elongator complex (Figs. 2*Q* and S21*B*). Similarly, the SASA of Met-AMP showed more variability in the initiator complex (Figs. 2*R* and S21*C*).

These results support the early release of Met-AMP from the protein’s active site in the initiator complex, leading to the variability in its size and solvent exposure. By contrast, the elongator complex maintained a comparatively stable ligand state within the protein’s binding pocket.

The observed Met-AMP instability in the initiator complex was strongly related to the early dissociation of initiator tRNA from the protein, relative to the elongator tRNA. The tRNA-protein interactions appear to trigger the opening of the protein’s active site, causing the ligand release. This complete event was observed over a shorter timescale in the initiator complex. While the elongator complex exhibited a similar tendency, the stable binding of elongator tRNA to MetRS prevented complete release of Met-AMP from the active site. These results show the essential role of tRNA in stabilizing Met-AMP binding.

Thus, the initiator tRNA reaction could be fast supported by its transient binding, which may also promote early ligand release. Conversely, the elongator tRNA reaction with MetRS could be comparatively slower, characterized by its binding for a substantial time and more ligand stability at the active site. These conclusions are consistent with the higher experimental K_m_ for the initiator tRNA (3.7 μM) compared to the elongator tRNA (2.6 μM) in *E. coli* (39).

Further, FEL-PCA of the ligands revealed higher average eigenvalues of EV1 and EV2 (of three runs) in the initiator ligand (1.4 ± 0.3 and 0.7 ± 0.3 nm^2^) than in the elongator (0.8 ± 0.5 and 0.3 ± 0.2 nm^2^) (Fig. S22*A*). Consistent with this, the EV1 *versus* EV2 plot showed a broader conformational landscape for the initiator ligand (Fig. S22*B*). RMSF analysis of ligand atoms clearly showed more movements in the initiator ligand relative to the elongator (Figs. S23 and 2*S*). These trends closely align with the dynamic landscape of tRNA, *i.e.*, EV1, EV2 and RMSF were higher for both the initiator tRNA and its ligand compared to the elongator counterparts.

In addition, FEL-PCA was performed on the complete initiator *versus* elongator complexes (*i.e.*, with protein, tRNA, and Met-AMP together). The major contribution to complex mobility was from tRNA and ligand molecules (details in Supplementary Section S4 and Fig. S24).

### Intra-molecular and solvent interactions showed distinguishing effects

The number of intra-molecular hydrogen bonds within protein and tRNA was analyzed over the final 500 ns trajectories (Fig. S25). The initiator complex exhibited a higher number of intra-protein hydrogen bonds and a lower number of intra-tRNA hydrogen bonds compared to the elongator complex. The average number (across three replicates) of intra-protein hydrogen bonds was 338 ± 11 in the initiator complex and 307 ± 7 in the elongator. Corresponding average intra-tRNA hydrogen bonds were 104 ± 2 and 116 ± 5, respectively. The initiator protein achieved a more stable conformation than the elongator due to the higher number of hydrogen bonds, consistent with its lower RMSD (discussed earlier). In contrast, initiator tRNA achieved a more open conformation due to the reduced number of intra-tRNA hydrogen bonds, correlating with its higher RMSD.

These findings imply that the increased motion and open conformation of the initiator tRNA resulted in the tRNA binding to the protein for a shorter time, causing a comparatively stable protein state due to fewer tRNA-protein interactions. In comparison, prolonged tRNA-protein contacts in the elongator complex induced more conformational changes in the protein.

Further, hydrogen bonding between water and protein, tRNA, and ligand (Met-AMP) was analyzed over the last 500 ns (Fig. S26). The number of protein-water hydrogen bonds was similar in the two complexes, with average values of 943 ± 21 and 935 ± 10 in the initiator and elongator complexes, respectively (Fig. S26*A*).

The tRNA-water hydrogen bond trajectories showed greater variability and lower average values in the initiator complex (520 ± 88) compared to the elongator (599 ± 7) (Fig. S26*B*). This suggests that the initiator tRNA may bind to distinct protein regions that reduced the tRNA-water contacts. Additionally, Met-AMP formed similar number of hydrogen bonds with water in the initiator (average = 11.7 ± 0.3) and the elongator complexes (average = 10.5 ± 1.0) (Fig. S26*C*).

### tRNA–protein interactions and plausible tRNA recognition mechanism

tRNA-protein interactions were analyzed for the equilibrated trajectories (final 500 ns; Figs. S27 and 4, *A* and *B*). The initiator complex showed fewer hydrogen bonds (average = 8 ± 2) compared to the elongator state (11 ± 2). The number of salt-bridges were similar in both states. However, minor differences were observed in other interactions (Fig. S27, *C*–*E*).

**Figure 4:**
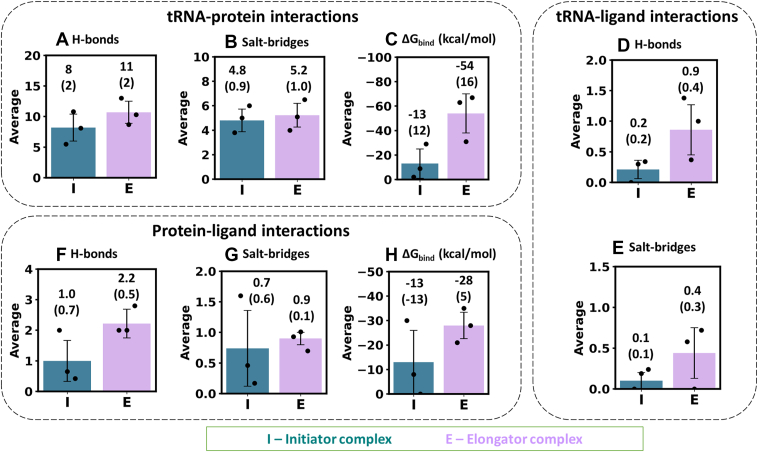
**Average binary interactions from three MD simulations for the initiator (I) and elongator (E) complexes, with their standard deviations (value in bracket; error bar).** Comparison of hydrogen bond counts, salt-bridge counts, and binding free energy (ΔG_bind_) values observed due to interactions between (*A*–*C*) tRNA and protein, (*D*–*E*) tRNA and ligand, and (*F*–*H*) protein and ligand. The statistically significant differences between the properties of the initiator and elongator complexes were analyzed using block averaging strategies and by performing t-tests (*p*-values) on the block-averaged data, as discussed in Supplementary Section 2 and shown in Tables S3 and S4.

**Figure 5:**
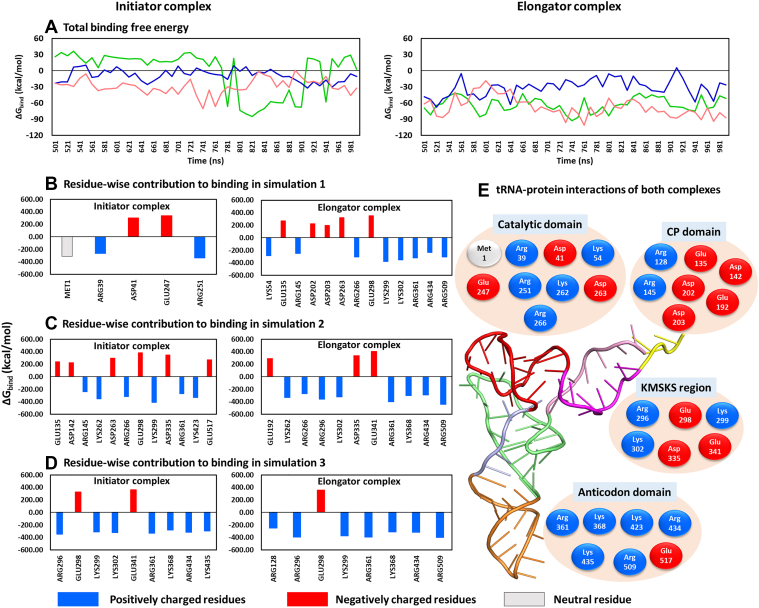
**ΔG_bind_ of tRNA-protein interactions in initiator and elongator complexes.***A*, ΔG_bind_ over three simulations. *B*–*D*, protein residue-wise impact on ΔG_bind_ in each run. A cutoff of ± 50 kcal/mol was used to identify prominent contributors. *E*, tRNA-interacting residues divided into protein regions. *Blue*, *red*, and *gray* represent positive, negative, and neutral residues.

Binding affinity (ΔG_bind_) between tRNA and MetRS was calculated over the final 500 ns simulations (Figs. 4*C* and 5*A*). Notably, the elongator tRNA exhibited a stronger binding to the protein (ΔG_bind_ = −54 ± 16 kcal/mol) compared to the initiator (ΔG_bind_ = −13 ± 12 kcal/mol). The stable binding of elongator tRNA to MetRS increased its binding affinity, whereas the transient binding of initiator tRNA reduced its affinity to the protein. The higher variability in the initiator tRNA binding was due to its greater flexibility and transient binding to multiple regions of MetRS.

These results indicate that hydrogen bonding and electrostatic interactions contribute substantially to tRNA-protein binding in both complexes, though they are reduced in the initiator state due to transient tRNA association. This reduced interaction led to weaker binding and earlier release of the initiator tRNA. In comparison, the elongator tRNA formed more stable contacts and stronger binding, increasing its residence time in the protein’s active site. These findings align well with experimental study on aminoacylation of the initiator and elongator tRNA by *E*. *coli* MetRS (39), which reported lower binding affinity of the initiator tRNA for MetRS compared to the elongator.

Analysis of protein residues contributing to tRNA binding showed that the favorable contacts were formed with arginine and lysine, while the unfavorable contacts occurred with aspartate and glutamate residues (Fig. 5, *B*–*E*). The main interactions occurred with the catalytic domain (1-115 and 226-292), the CP domain (116–225), the KMSKS loop region (293–350), and the anticodon domain (358–519) (Fig. 5*E*). The catalytic domain primarily formed electrostatic attractions (blue colored residues in Fig. 5*E*), contributing to tRNA binding to the active site. This event might help in the tRNA charging with methionine.

The CP domain contributed mainly electrostatic repulsive forces with tRNA *via* its negatively charged residues (five residues, red color in Fig. 5*E*), although two arginine residues (Arg128 and Arg145) formed favorable contacts. The KMSKS loop (residues 299–303) is conserved in MetRS (22) and is located at the entrance of the active site (Fig. 1*A*), forming an anchor that might stabilize the ligand Met-AMP. This loop primarily enhanced tRNA binding *via* Lys299 and Lys302. The region surrounding this loop also showed repulsive binding to tRNA *via* Glu298, Asp335, and Glu341. The attractive and repulsive forces between tRNA and the protein’s active site led to the higher fluctuation in the CP domain and KMSKS region (shown in RMSF analysis) potentially opening the active site. This may be required to accommodate the tRNA’s 3′ CCA end (acceptor stem) for productive interaction with Met-AMP and subsequent product release.

During these events, tRNA remained strongly bound (electrostatic attractions) *via* its anticodon region to the anticodon binding domain of the protein (Fig. 5*E*). The tRNA anticodon interacted favorably with arginine (Arg361, Arg434 and Arg509) and lysine (Lys368, Lys423 and Lys435) residues. This strong anticodon binding could facilitate the tRNA charging at the active site by maintaining the productive tRNA conformation.

Literature evidence shows that KMSKS loop flips upon amino acid activation and reaches the closed conformation after Met-AMP formation (41). It has been proposed that binding of the tRNA to the anticodon domain facilitates the access of 3′ CCA to the active site by opening the KMSKS loop (42). In *E. coli* MetRS, a mutation at residue Arg533 in the anticodon domain affects the aminoacylation rate (43). This residue was presumed to cause productive 3′ CCA binding to the KMSKS and CP domain (43). We observed that the equivalent *Mtb* MetRS residue Arg509 interacts with tRNA, supporting this mechanism. Further, mutagenesis in *E. coli* MetRS have demonstrated that the CP domain plays a central role in methionine activation and transfer to the tRNA’s 3′ CCA end (44, 45). The CP domain mobility has been linked to these catalytic steps (39, 46, 47), it has been proposed to guide the tRNA’s acceptor arm into the active site (42). Collectively, our observations are well supported by experimental findings in literature.

### Elongator complex displayed more tRNA-ligand and protein-ligand interactions

The initiator tRNA interacted with Met-AMP for a shorter duration, while the elongator tRNA maintained hydrogen bonds and salt-bridges with the ligand for a longer period (Fig. S28). The average number of hydrogen bonds and salt-bridges in the initiator complex was 0.2 ± 0.2 and 0.1 ± 0.1, respectively, compared to 0.9 ± 0.4 and 0.4 ± 0.3 in the elongator complex (Fig. 4, *D* and *E*).

These results suggest that, while maintaining a stable state in the protein’s active site, Met-AMP forms favorable contacts with tRNA. These attractive tRNA-ligand contacts work simultaneously with repulsive forces between the tRNA and the protein’s active site. The interplay between these opposite forces, acting in parallel, likely influences the ligand binding to tRNA and active site opening, facilitating the reaction and product release.

The protein-Met-AMP complex exhibited more hydrogen bonds in the elongator complex (2.2 ± 0.5) than in the initiator (1.0 ± 0.7) (Figs. S29 and 4*F*). Minor variations in other interactions were observed (Fig. S29, *B*–*E* and 4*G*). Importantly, stronger protein-ligand binding occurred in the elongator state (ΔG_bind_ = −28 ± 5 kcal/mol) than in the initiator (−13 ± 13 kcal/mol) (Figs. S30*A* and 4*H*). In the elongator complex, Tyr12, Asn14, His21, Glu24, Lys54, Phe292, Lys302, Ser 303 and Val304 mainly contributed to ligand binding (Fig. S30, *B*–*E*). In contrast, the initiator complex showed fewer contributing residues, consistent with the ligand dissociation (Fig. S30, *B*–*D*). Additionally, interactions involving Mg^2+^ were analyzed and are detailed in Figure S31. Mg^2+^ primarily formed ionic contacts with the tRNA.

### Initiator *versus* elongator tRNA features

We analyzed characteristic features of tRNA reported in the literature and checked their presence in our simulations. Two typical features of initiator tRNA include the absence of a hydrogen bond between nucleotides C1 and U73, and the presence of two hydrogen bonds between A11 and U25 (28). Our simulations showed a similar trend, with an average of 0.02 ± 0.03 hydrogen bonds between C1 and U73, and 1.76 ± 0.06 between A11 and U25 (Fig. S32, *A* and *B*, and S33, *A* and *B*). Similarly, elongator tRNA is known to form two hydrogen bonds between nucleotides G1 and U73 (48, 49). We found a consistent bonding pattern of mainly two hydrogen bonds (Fig. S32*C*), with an average of 1.1 ± 0.1 across simulations (Fig. S33*C*).

The absence of a hydrogen bond between C1 and U73 in the initiator tRNA may confer conformational freedom, facilitating faster access of the 3′ CCA end to the protein’s active site and enabling aminoacylation on a shorter timescale. In contrast, the presence of G1-U73 hydrogen bonds in elongator tRNA likely stabilizes the acceptor stem and restricts its conformational unwinding, resulting in a longer timescale for tRNA charging. This validation is also strengthened by another experimental fact, which reported a shorter lifetime for the initiator complex and a longer one for the elongator in *E. coli* (39).

The methionine tRNA anticodon sequence CAU (positions 35–37) is conserved and serves as a main recognition element for interaction with the MetRS anticodon domain (residues 358–519) during aminoacylation (14, 50). Therefore, we analyzed hydrogen bonding between the CAU bases and the protein (Figs. S34 and S33, *D*–*F*). All three bases formed hydrogen bonds in both the initiator and elongator complexes. The protein formed an average of 1.6 ± 0.8 hydrogen bonds with C35, 0.6 ± 0.7 with A36, and 0.3 ± 0.3 with U37 in the initiator complex, and 0.1 ± 0.1, 0.4 ± 0.5, and 0.6 ± 0.3 hydrogen bonds with the respective bases in the elongator complex. Therefore, CAU bases exhibited a tendency to form hydrogen bonds with protein in both initiator and elongator complexes.

### tRNA recognition by anticodon and active site of MetRS

The anticodon binding domain of MetRS performs an essential function of tRNA anticodon binding, primarily through interactions with three conserved residues, Asn357, Arg361, and Trp431 (Fig. S35) (14, 51, 52). Hydrogen bonding between these residues and the tRNA was analyzed (Figs. S36 and S33, *G*–*I*). The average number of hydrogen bonds formed by initiator and elongator tRNA with Asn357 were 0.9 ± 0.8 and 0.4 ± 0.3, respectively, with Arg361 were 0.4 ± 0.4 and 0.7 ± 0.1, and with Trp431 were 0.04 ± 0.03 and 0.08 ± 0.04. While all three residues engaged in meaningful interactions with tRNA, prominent contacts were observed with Asn357 and Arg361.

The CCA terminal (75–77 position) of tRNA is conserved and plays a key functional role in the aminoacylation of the correct amino acid (53, 54). Its interaction analysis with both protein and ligand (Met-AMP) revealed that the CCA region of the elongator tRNA typically formed more hydrogen bonds than the initiator (Figs. S37, S38 and S33, *J*–*O*). The protein formed average hydrogen bonds of 0.07 ± 0.05 and 1.36 ± 0.61 with C75, 0.3 ± 0.1 and 0.9 ± 0.2 with C76, and 1.1 ± 0.4 and 1.1 ± 0.7 with A77, of initiator and elongator tRNA, respectively. Interestingly, the ligand did not form any hydrogen bonds with CCA bases in the initiator state, possibly due to its release from the binding site. In contrast, the ligand in the elongator complex formed average hydrogen bonds of 0.3 ± 0.2, 0.1 ± 0.1, and 0.1 ± 0.1 with C75, C76, and A77, respectively (Figs. S38, S33, *M*–*O*).

**Figure 6:**
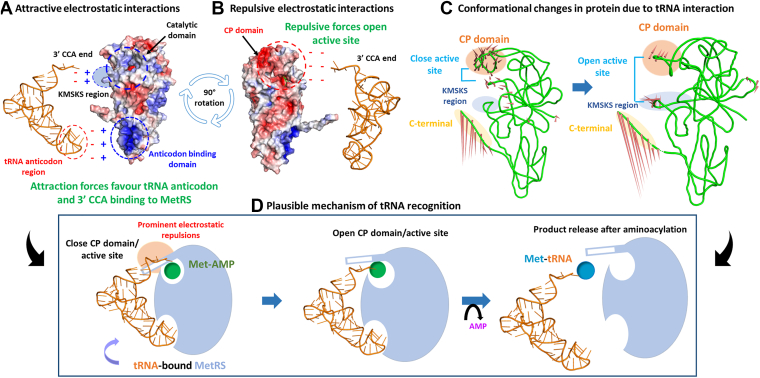
**Plausible mechanism of tRNA recognition by *Mtb MetRS*.***A*, attractive interactions favor tRNA binding *via* its anticodon and acceptor arm to the MetRS anticodon domain and active site, respectively. *B*, the negatively charged CP domain forms repulsive contacts with the acceptor arm, inducing active site opening. *C*, MetRS achieves close to open conformation during tRNA interactions, an event favorable for the tRNA entry into the site and product release. *D*, the CP knuckle opens, allowing tRNA to enter the active site and interact with Met-AMP, leading to product formation.

These findings highlight that the CCA terminal of the tRNA forms hydrogen bonds with both the ligand and the protein, which may enable the progression of the aminoacylation reaction. Meanwhile, electrostatic interactions between the tRNA and the protein’s active site may facilitate tRNA accommodation and product release by attaining an open conformation of the active site. The overall plausible mechanism of tRNA recognition by *Mtb* MetRS is summarized in Figure 6 (details in Supplementary Sections S5, S6 and Figs. S39, S40).

### Hairpin conformation of the tRNA acceptor stem

**Figure 7:**
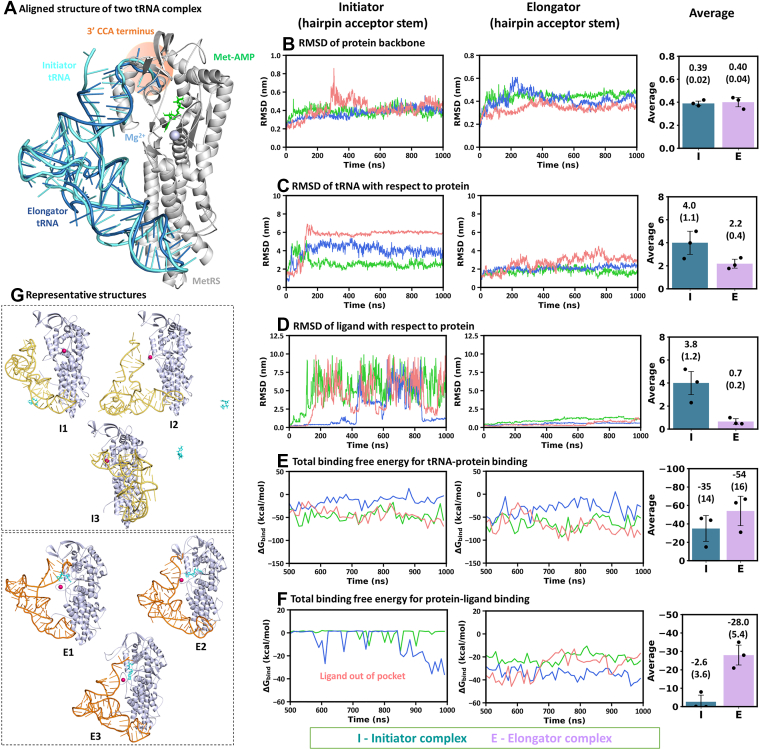
**Comparison of tRNA-complexes in “hairpin” acceptor stem.***A*, aligned structure of initiator and elongator tRNA complex with “hairpin” acceptor stem conformation. RMSD of (*B*) protein backbone, (*C*) tRNA, and (*D*) ligand. Binding free energy between (*E*) tRNA and protein and (*F*) ligand and protein. *G*, representative structures of initiator (I1, I2, and I3) and elongator (E1, E2, and E3) complexes from three simulations. The statistically significant differences between the properties of the initiator and elongator complexes were analyzed using block averaging strategies and by performing t-tests (*p*-values) on the block-averaged data, as discussed in Supplementary Section 2 and shown in Tables S5 and S6.

We observed differential behavior between the initiator and elongator states of MetRS, tRNA, and Met-AMP. We further examined how much the results depended on the choice of input conformations. The major difference in the input geometry was in the 3′ CCA terminus of the tRNA acceptor stem (Fig. 1*E*), which was present in an extended conformation in the initiator tRNA, while it exhibited a hairpin structure in the elongator tRNA. The hairpin conformation in the elongator tRNA might contribute to its higher stability compared to the extended CCA terminus of the initiator tRNA. The acceptor stem of the elongator tRNA was modeled using the PDB structure 4AQ7 as a template (Fig. S41). Therefore, we prepared the hairpin structure of the initiator tRNA acceptor stem using the same template, 4AQ7 (Fig. 7*A*). In this hairpin geometry, the 3′ terminal ribose ring oxygen atoms of initiator and elongator tRNAs were within 0.28 nm, both approaching the active site to the same extent. Some conformational differences were evident, primarily due to the differential composition of the two tRNAs and their intramolecular bonding patterns.

MD simulations of the initiator complex with the hairpin acceptor stem of tRNA were performed in three replicates for 1 μs each (1 μs × 3 runs = 3 μs) and analyzed in the same way as earlier. These simulations were compared with the elongator state simulations (hairpin tRNA acceptor stem) (Figs. 7, *B*–*G* and S42–S56). Interestingly, the distinctive pattern between the initiator and elongator states remained the same as observed earlier. The acceptor stem of the initiator tRNA in all three simulations left the MetRS active site and attained distinct conformations (Fig. 7*C*). The average RMSD of the initiator tRNA was higher (4.0 ± 1.1 nm) than that of the elongator tRNA (2.2 ± 0.4 nm). The Met-AMP ligand also showed similar behavior, *i.e.*, it moved away from the active site in all three simulations of the initiator state (average RMSD = 3.8 ± 1.2 nm; Fig. 7*D*), while it stayed consistently at the active site in the elongator state (0.7 ± 0.2 nm). These differences in tRNA and Met-AMP binding to the MetRS active site also led to their differential binding affinity between the initiator and elongator states (Fig. 7, *E* and *F*). The initiator tRNA showed decreased affinity (−35 ± 14 kcal/mol) to the protein compared to the elongator tRNA (−54 ± 16 kcal/mol). Likewise, Met-AMP exhibited decreased affinity to MetRS in the initiator state (−2.6 ± 3.6 kcal/mol) than in the elongator state (−28.0 ± 5.4 kcal/mol). These results signify that the observed differential behavior between the initiator and elongator state dynamics was robust.

The representative structures from MD simulations were compared between the initiator and elongator complexes (Fig. 7*G*). In all three representatives of the initiator state, the tRNA moved away from the MetRS active site and attained multiple conformations, suggesting transient binding of the initiator tRNA. In contrast, the elongator tRNA consistently formed interactions with the active site, indicating its long lifetime. The major cause of the transient initiator tRNA binding may be the lack of a hydrogen bond between the C1 and U73 nucleotides in the acceptor stem of the initiator tRNA, which is present in the elongator tRNA (G1 and U73) (28). This differential feature may confer greater stability to the elongator tRNA at the MetRS active site. In addition, our observations on the short and long lifetimes of the initiator and elongator tRNAs, respectively, are well supported by experimental evidence indicating the same distinct behavior (39, 40, 42, 44, 45, 46, 47). Experimental data suggest a higher K_m_ for the initiator tRNA (3.7 μM) compared to the elongator tRNA (2.6 μM) in *E. coli* (39), which aligns well with our observations. Thus, our results provide strong evidence for the distinctive behaviors of the initiator and elongator tRNAs, consistent with previous literature reports.

## Conclusion

Charging of tRNA by MetRS is a critical step in protein synthesis. MetRS, which participates in both the initiation and elongation phases of translation, is an essential target for anti-tubercular drug design. The two methionine tRNAs (each 77 bases in length) corresponding to the initiation and elongation processes share a sequence identity of 67.9%. Due to their sequential and functional differences, these tRNAs are expected to bind differentially to MetRS. In this study, we identified distinct conformational and binding characteristics of the tRNA-MetRS complexes (in the presence of Met-AMP) in the two functional states. The initiator tRNA exhibited higher conformational changes and transient binding to the protein, while the elongator tRNA displayed more stable binding to MetRS. However, both tRNAs maintained the intra-tRNA interactions necessary for the typical cloverleaf structure. These results conclude that initiator tRNA charging with methionine may occur on a shorter timescale than that of the elongator tRNA. This shorter timescale of the initiator tRNA could be linked to its necessity for initiating protein synthesis. The tRNA interacts with two protein pockets: the tRNA CCA terminal engages with the protein’s active site (aminoacylation site), while the tRNA anticodon (CAU, positions 35–37) binds to the protein’s anticodon domain. The tRNA charging at these two protein sites can be divided into three mechanistic parts: (a) Electrostatic attractions between the tRNA and the protein’s catalytic domain could help tRNA charging with methionine; (b) repulsive forces of tRNA with the protein’s connective peptide, along with both repulsive and attractive contacts with the KMSKS region could open the active site to accommodate the tRNA and cause product release; (c) and strong electrostatic binding of the tRNA anticodon to the protein’s anticodon domain could facilitate the reaction progression by maintaining the productive tRNA conformation. These events indicate a structural and electrostatic coupling between the two tRNA binding sites on MetRS. Our study provides a mechanistic framework for how *Mtb* MetRS distinguishes and processes two sequentially divergent tRNAs and the plausible tRNA charging mechanism through conformational flexibility and electrostatic complementarity.

## Method

### tRNA-protein complex building

The tRNA-bound structure for *Mtb* MetRS was absent in the Protein Data Bank (PDB) (55). However, a 3D structure of initiator tRNA complexed with the ribosome was available for *Mycobacterium smegmatis* (PDB structure: 8FR8), a close homolog of *Mtb* (56). Both species shares 100% sequence identity for the initiator tRNA (57). Therefore, we used the 3D coordinates of *M. smegmatis* initiator tRNA to build the corresponding *Mtb* initiator tRNA (tRNA^fMet^). For the elongator tRNA, no experimental structure of *Mtb* tRNA^Met^ or a homolog was available. Thus, we modeled its secondary structure using RNAstructure (58) and generated the 3D coordinates using RNAComposer (Fig. 1*E*) (59).

The structure of *Mtb* MetRS was retrieved from the PDB (PDB ID: 5XET) (22) (Fig. 1*A*). We used the protein bound to the intermediate (Met-AMP) and one magnesium ion at the active site. Missing protein residues were added using CHARMM-GUI (60).

To build the starting geometries of the protein-tRNA complexes for both initiator and elongator states of *Mtb* MetRS, we used the MetRS-tRNA structure from *Aquifex aeolicus* in the intermediate state (containing Met-AMP; PDB code: 2CT8) (Fig. S2) (14). The MetRS sequence identity between *Mtb* and *A. aeolicus* is 41.3%. The *A. aeolicus* tRNA (in PDB 2CT8) shares 72.5% sequence identity with the *Mtb* elongator tRNA and 66.7% with *Mtb* initiator tRNA (based on EMBOSS Needle alignment). Therefore, we structurally aligned the *Mtb* MetRS and its associated tRNAs to the *A. aeolicus* MetRS-tRNA complex to construct the starting geometries.

### MD simulations of tRNA-protein complexes

The two modeled *Mtb* tRNA-MetRS complexes were prepared at pH 7 in a rectangular box using CHARMM-GUI (60). The total charge of each system was neutralized, and a salt concentration of 0.15 M was maintained by adding the required Na^+^ and Cl^-^ ions. The CHARMM36 force field was applied (61), and MD simulations were performed using GROMACS (62) at 300 K and 1 bar. The systems were subjected to two-step equilibrations (NPT and NVT) for 100 picoseconds each. The V-rescale thermostat and C-rescale barostat were used for temperature and pressure coupling, respectively (63). Three replica simulations of each system were conducted using NPT for 1000 nanoseconds (ns), with a time-step of 2 femtoseconds.

### Statistical analysis and molecular interactions

Primary analysis was performed over the entire MD trajectory (1000 ns). This involved computing the protein backbone root mean square deviation (RMSD), radius of gyration (R_g_), and total solvent-accessible surface area (SASA), as well as the hydrophilic and hydrophobic components of SASA. Similarly, RMSD, R_g_, and SASA were computed for the tRNA and the ligand (Met-AMP).

Secondary analysis was conducted on the final 500 ns of the MD trajectory. Free energy landscape principal component analysis (FEL-PCA) was performed on individual molecules, *i.e.*, protein, tRNA, and ligand, and additionally on their complex form. RMSF and the number of inter- and intra-molecular interactions were also computed. The ratio of intra-molecular contact for protein and tRNA residues was determined as the total number of contacts divided by their respective mean value. Protein secondary structures were analyzed using the VMD-SS plugin (64). The binding free energy (ΔG_bind_) was calculated for protein-tRNA and protein-ligand binding using gmx_MMPBSA (65) at 10 ns intervals. In addition, representative structures for the six simulated trajectories were extracted from the final 500 ns runs. Clustering was performed using GROMOS (66), with an RMSD cutoff of 0.4 nm. From the most populated cluster, the central structure was selected as representative.

Data were represented as mean ± standard deviation in the text and bar plots. The statistically significant differences between the properties of the initiator and elongator complexes were evaluated using block averaging strategies and by performing two-tailed paired t-tests (*p*-value < 0.05) on the block-averaged data, as detailed in Supplementary Section S2. The time-dependent properties from MD simulations were divided into non-overlapping blocks of equal size. The mean value of each block was calculated, and the averages of these block means were compared between the initiator and elongator complexes using t-tests. The normal distribution of the data was tested using Shapiro-Wilk and D′Agostino-Pearson tests.

## Data availability

All data are provided in the manuscript and supplementary files. The simulation input and output data, along with trajectory videos are deposited in the Zenodo repository (https://doi.org/10.5281/zenodo.17358357).

## Supporting information

This article contains supporting information (14, 18, 51, 67, 68, 69, 70) (https://www.blopig.com/blog/2025/05/estimating-uncertainty-in-md-observables-using-block-averaging/#more-12609) (71, 72, 73, 74).

## Conflict of interest

The authors declare that they have no conflicts of interest with the contents of this article.
