# Supplementary material for: Initiator and elongator tRNA recognition mechanism in Mycobacterium tuberculosis methionyl-tRNA synthetase
Source: J Biol Chem. 2025 Nov 14;302(1):110939. doi: 10.1016/j.jbc.2025.110939 (PMC12757634; doi:10.1016/j.jbc.2025.110939)
Supplement: Supporting information [file mmc1.pdf]

Supplementary information

Initiator and elongator tRNA recognition mechanism in *Mycobacterium tuberculosis* methionyl-tRNA synthetase

Shivani Thakur[1], Rukmankesh Mehra[1,2,*]

[1]*Department of Chemistry, Indian Institute of Technology Bhilai, Durg 491002, Chhattisgarh, India.*

[2]*Department of Bioscience and Biomedical Engineering, Indian Institute of Technology Bhilai, Durg 491002, Chhattisgarh, India.*

[*]Correspondence: rukmankesh@iitbhilai.ac.in

ORCID

Rukmankesh Mehra: 0000-0001-6010-1514

Shivani Thakur: 0000-0002-7869-6648

Table of contents

Supplementary sections

Section S1. Nucleotide sequence alignment of tRNAs

Section S2. Statistical analysis using block averaging of MD simulations

Section S3. Free energy landscape principal component analysis of protein showed similar conformational landscapes

Section S4. Differential motion of the complete initiator versus elongator complexes

Section S5. Electrostatic surface charge analysis of protein

Section S6. Plausible mechanism of tRNA interaction and recognition

Supplementary tables

Table S1. Nucleotide sequence alignment of tRNA sequences of *Mtb.*

Table S2. The average and standard deviation values of each property.

Table S3. The t-test results for comparison of protein, tRNA, and ligand properties between the initiator and elongator complexes.

Table S4. The t-test results for comparison of protein, tRNA, and ligand properties between the initiator and elongator complex at fixed block size.

Table S5. The t-test results for comparison of protein, tRNA, and ligand properties between the initiator (hairpin acceptor stem) and elongator complex (hairpin acceptor stem).

Table S6. The t-test results for comparison of protein, tRNA, and ligand properties between the initiator (hairpin acceptor stem) and elongator complex (hairpin acceptor stem) at fixed block size.

Supplementary figures

Figure S1. Nucleotide sequence alignment of tRNAs.

Figure S2. Steps involved in tRNA-complex building.

Figure S3. The simulated properties of MetRS protein.

Figure S4. Hydrophobic and hydrophilic solvent-accessible surface area (SASA) analysis of *Mtb* MetRS protein.

Figure S5. Free energy landscape principal component analysis (FEL-PCA) of protein.

Figure S6. Mobility of protein in initiator complex.

Figure S7. Mobility of protein in elongator complex.

Figure S8. Residue-wise RMSF of MetRS protein.

Figure S9. Residue-wise analysis of contacts in MetRS upon tRNA binding.

Figure S10. Protein secondary structure elements.

Figure S11. Residue-wise secondary structure assignment of MetRS.

Figure S12. Representative structures from each simulation run of initiator and elongator tRNA.

Figure S13. Trajectory snapshots from simulation 1 of the initiator complex.

Figure S14. Trajectory snapshots from simulation 2 of the initiator complex.

Figure S15. Trajectory snapshots from simulation 3 of the initiator complex.

Figure S16. Trajectory snapshots from simulation 1 of the elongator complex.

Figure S17. Trajectory snapshots from simulation 2 of the elongator complex.

Figure S18. Trajectory snapshots from simulation 3 of the elongator complex.

Figure S19. Nucleotide-wise RMSF analysis of tRNA.

Figure S20. Nucleotide-wise contact analysis of tRNA.

Figure S21. Structural properties of the ligand (Met-AMP) in the initiator and elongator complexes.

Figure S22. PCA of the ligand (Met-AMP) in initiator and elongator complexes.

Figure S23. RMSF analysis of heavy atoms in the ligand molecule.

Figure S24. PCA of complex (containing protein, ligand and tRNA) in initiator and elongator complexes.

Figure S25. Intramolecular hydrogen bonding of protein and tRNA.

Figure S26. Hydrogen bonds with water in initiator (I) and elongator (E) complexes.

Figure S27. Intermolecular interactions between tRNA and protein in initiator and elongator complexes.

Figure S28. Intermolecular interactions between tRNA and ligand in initiator (I) and elongator (E) complexes.

Figure S29. Intermolecular interactions between protein and ligand in initiator (I) and elongator (E) complexes.

Figure S30. Binding energy for protein-ligand binding in initiator and elongator complexes.

Figure S31. Interactions of the Mg^{2+} ion in the initiator (I) and elongator (E) complexes.

Figure S32. Base-specific hydrogen bonding patterns in initiator and elongator tRNAs.

Figure S33. Average and standard value (value in bracket; error bar) comparison of H-bond counts for tRNA centric interactions.

Figure S34. Hydrogen bonding interactions formed between CAU (35-37) anticodon and protein residues during the simulations.

Figure S35. Conserved residues in anticodon domain of MetRS (shown in purple) from 26 different species.

Figure S36. Hydrogen bonding between three conserved protein residues of anticodon domain and tRNA in initiator and elongator complexes.

Figure S37. Hydrogen bond interactions formed by CCA (75-77) end of tRNA with protein residues.

Figure S38. Hydrogen bonding interactions formed by CCA end (75-77) with ligand (Met-AMP).

Figure S39. Electrostatic potential surface of representative structures.

Figure S40. Interactions observed between protein residues, ligand and tRNA nucleotides in representative structures.

Figure S41. The details of templates used by RNAComposer to model elongator tRNA based on sequence.

Figure S42. Comparisons of simulated average properties and standard deviations (value in bracket; error bar) from three independent MD runs for initiator (I) versus elongator (E) complexes with hairpin acceptor stem.

Figure S43. The simulated properties of MetRS protein.

Figure S44. Free energy landscape principal component analysis (FEL-PCA) of protein.

Figure S45. Residue-wise RMSF of MetRS protein.

Figure S46. Residue-wise analysis of contacts in MetRS upon tRNA binding for (a) initiator and (b) elongator complexes over three simulations.

Figure S47. Comparisons of simulated properties of initiator and elongator tRNAs.

Figure S48. Principal component analysis of tRNA.

Figure S49. Nucleotide-wise RMSF analysis of tRNA.

Figure S50. Nucleotide-wise contact analysis of tRNA.

Figure S51. Structural properties of the ligand (Met-AMP) in the initiator and elongator complexes.

Figure S52. RMSF analysis of heavy atoms in the ligand molecule.

Figure S53. Representative structures from each simulation run of initiator and elongator tRNA.

Figure S54. Trajectory snapshots from simulation 1 of the initiator complex (hairpin acceptor stem).

Figure S55. Trajectory snapshots from simulation 2 of the initiator complex (hairpin acceptor stem).

Figure S56. Trajectory snapshots from simulation 3 of the initiator complex (hairpin acceptor stem).

Section S1. tRNA sequence analysis

Mtb contains three genes, *metU*, *metT,* and *metV*, that encode tRNAs with the CAU anticodon (67). The *metU* and *metT* genes encode the initiator tRNA ($tRNA^{fMet}$) and the elongator tRNA ($tRNA^{Met}$), respectively, to process methionine (**Figure 1c-f** and **S1a**). In contrast, the post-transcriptional modification of tRNA encoded by *metV* leads to an LAU anticodon (where L is lysidine) that processes isoleucine ($tRNA^{Ile2}$) instead of methionine (14,18).

The nucleotide sequences of $tRNA^{fMet}$ and $tRNA^{Met}$ were retrieved from RNAcentral (68) (**Figure S1a**). Each tRNA is 77 nucleotides in length. Pairwise local and global sequence alignments of $tRNA^{fMet}$ and $tRNA^{Met}$ were performed using BLASTN (69) and EMBOSS Needle, respectively (70). $tRNA^{fMet}$ (initiator tRNA) showed no significant local similarity to $tRNA^{Met}$ (elongator tRNA) in BLASTN analysis (**Table S1**). However, EMBOSS revealed moderate global identity between $tRNA^{fMet}$ and $tRNA^{Met}$ (67.9%), suggesting partial conservation across the complete sequences (**Table S1**). Notably, the anticodon loop ($_{33}$CUCAUAA$_{39}$; **Figure S1b**) was conserved across two tRNAs, showing its importance in tRNA recognition

Section S2. Statistical analysis using block averaging of MD simulations

To test the statistically significant differences between the properties of the initiator and elongator complexes, we represented the simulation trajectories in blocks and computed averages and p-values using two-tailed paired t-tests. The time-dependent properties from MD simulations were divided into non-overlapping blocks of equal size. The mean value of each block was calculated, and the averages of these block means were compared between the initiator and elongator complexes using t-tests. This was done because the sample size of three MD simulation replicates was not sufficient for a meaningful t-test due to the inherent variability associated with random velocity seeds. In contrast, the sample size of 3003 (1001 frames × 3 MD replicas) or 1503 (501 frames × 3 replicas), corresponding to the full 1 μs or last 500 ns trajectories, may show highly significant effects even with a small average difference. Additionally, the MD trajectories exhibit autocorrelation, which further restricts treating all frames as independent samples for comparison. Therefore, we used block averaging in two ways that possibly account for autocorrelation (71-74).

In the first strategy, block size was determined using "the standard error of the block means" referred to as "block standard error (BSE)." The block sizes were varied from 10 to 200 with an interval of 5 and plotted against BSE to determine the appropriate block size. On increasing the block size, BSE increases till certain point and then achieves a plateau. The optimal block size was the smallest block size for which the relative change in BSE on further increasing the block size was less than the convergence threshold (5%).

BSE was determined using the formula:

$$BSE = \frac{\sigma_{\bar{x}}}{\sqrt{n}}$$

where $\sigma_{\bar{x}}$ is the standard deviation of block means, and n is number of blocks.

Relative change in BSE was determined using the formula:

$$Relative\ change\ in\ BSE = \frac{|BSE_i - BSE_{i-1}|}{BSE_{i-1}}$$

Using this strategy, the block size for each property of both the initiator and elongator complexes was obtained and the larger block size among initiator and elongator was considered as final block size for a particular property. The mean value of each block was calculated, and the average of the block means was compared between the initiator and elongator complexes using t-tests. This approach ensures that block sizes are sufficiently large to eliminate autocorrelation while retaining an adequate number of blocks for reliable statistical averaging.

The second approach used a constant block size of 100 frames for t-tests, thereby leading to a sample size of 30 (from 3003 frames for three 1 μs simulations) or 15 (from 1503 frames for three last 500 ns). Both of these approaches represent approximate ways of dealing with MD trajectories while analyzing statistically significant differences, as reported in the literature (71-74). These block averaging analyses revealed statistically significant differences (p-value < 0.05) between the properties of the initiator and elongator complexes, based on which conclusions were drawn. The p-values for the time-dependent properties, along with the block size and average, are shown in **Supplementary Tables S3-S6.**

Section S3. Free energy landscape principal component analysis of protein showed similar conformational landscapes

Free energy landscape principal component analysis (FEL-PCA) was performed to investigate the dominant motions of the protein in the initiator and elongator tRNA complexes (**Figure S5**). The first two eigenvectors (EV1 and EV2) accounted for a significant portion of the total motion. The plots of eigenvalues versus eigenvectors and EV1 versus EV2 showed that both initiator and elongator complexes exhibited a similar behavior. The initiator complex displayed the average of three runs for EV1 and EV2 as 12 ± 6 nm^2 and 6 ± 3 nm^2, and the elongator complex showed 12 ± 5 nm^2 and 5 ± 2 nm^2, respectively. This indicates relatively similar conformational landscapes of protein in both systems. Additionally, the rapid decay of eigenvalues after the first few components in both systems suggests that a limited number of principal components capture the majority of essential motions.

Section S4. Differential motion of the complete initiator versus elongator complexes

We analyzed the FEL-PCA analysis of the complete initiator versus elongator complexes, i.e., collectively for protein, ligand, and tRNA (**Figure S24**). We observed a higher motion for the initiator complex. The average EV1 and EV2 values for three runs were 4605 ± 5719 and 2662 ± 3218 nm^2 for the initiator complex, whereas the corresponding values were 149 ± 22 and 63 ± 22 nm^2 for the elongator. These values indicate the higher motion of the initiator complex, which was consistent with the above observations of higher tRNA and ligand mobility in the initiator complex. This means that the major contribution to complex mobility was from tRNA and ligand molecules, while protein contributed to it to a smaller extent.

Section S5. Electrostatic surface charge analysis of protein

Electrostatic potential surface analysis of representative protein structures (**Figure S12 and S39**) revealed key differences in electrostatic patterns across three major tRNA interaction regions: (i) the CP1 domain, (ii) the anticodon-binding domain (ABD), and (iii) the KMSKS loop. These differences are closely associated with the conformational and energetic adaptations necessary for tRNA binding and ligand coordination.

In the CP1 domain, the negatively charged phosphate backbone of the tRNA interacts with the acidic residues in the CP knuckle region, generating repulsive forces. These repulsions appear to trigger conformational rearrangements in the CP knuckle loop, eventually opening the pocket to accommodate the CCA end of the tRNA for productive interaction with the Met-AMP ligand. This structural reorganization is energetically favorable, as it reduces unfavorable electrostatic interactions and enables precise positioning of the tRNA's 3′ end in the active site, facilitating efficient methionine charging.

In the ABD, a cluster of basic residues particularly Arg361 (conserved), Lys368, Arg373, Arg434, Lys435, and Arg444 forms a positively charged electrostatic surface. These residues, located within positions 357-373 and 430-445, enable direct and stable recognition of the anticodon loop through long-range electrostatic attractions, without inducing major conformational changes in the domain, as confirmed by the low RMSF values observed in this region (**Figure S8**). These interactions stabilize tRNA binding and contribute favorably to the overall binding free energy through persistent hydrogen bonding and electrostatic complementarity.

In the KMSKS loop region, interactions between the negatively charged tRNA backbone and positively charged residues (e.g., lysine's and arginine's) create an attractive electrostatic field that brings the loop closer to the tRNA (**Figure S39**). This movement is supported by increased flexibility of the KMSKS loop, as indicated by high RMSF values (**Figure S8**). This conformational adjustment partially opens the ligand-binding pocket, weakening ligand-protein interactions as reflected in the increased ligand flexibility and exposure observed in the initiator complex. This shift may enhance the possibility of ligand-tRNA interactions, thereby altering the structural dynamics and binding energetics within the active site.

In summary, these findings reinforce the pivotal role of electrostatic complementarity in governing tRNA recognition, ligand coordination, and dynamic rearrangements within *Mtb* MetRS complexes, contributing to the functional specificity and stability of initiator and elongator tRNA interactions.

Section S6. Plausible mechanism of tRNA interaction and recognition

The molecular recognition of tRNAs by *Mtb* MetRS involves a coordinated interplay of electrostatic complementarity and structural adaptation. Electrostatic surface analysis of representative structures revealed three primary tRNA-binding regions on the protein: the CP1 domain, ABD, and the KMSKS loop. The phosphate backbone of the tRNA, being negatively charged, is electrostatically attracted to positively charged lysine and arginine residues in the ABD and KMSKS regions. Importantly, positively charged residues such as Arg361, Lys368, Arg373, and Arg444 within the ABD stabilize tRNA binding by favoring long-range electrostatic interactions without requiring significant conformational rearrangements. In contrast, the interaction with the CP1 domain (acidic residues) triggers repulsion, prompting a shift from a closed to an open conformation in the CP knuckle loop (residues 127-154; **Figure S8**) (51). tRNA binding in elongator complex induced secondary structural rearrangements in the CP domain of *Mtb* MetRS, including β-sheet-to-coil transition and loss of a 3_{10}-helix. This conformational and secondary structure transitions suggest remodeling of active site which might be essential for accommodating the 3′ CCA end of the tRNA in the catalytic pocket. These initial recognition events may contribute to specificity and secure the tRNA in a favorable orientation for subsequent steps of aminoacylation.

Upon binding, elongator and initiator tRNAs demonstrate distinct interaction dynamics. The elongator tRNA adopts a more stable conformation, consistently engaging both the CP domain and the anticodon-binding region, which is reflected in its strong binding affinity and lower structural fluctuations. In contrast, the initiator tRNA shows flexible binding modes, including back-face interaction via the TΨC loop - a feature reminiscent of class II aaRS recognition (73). This divergence in binding behavior highlights the idiosyncratic recognition strategy of the initiator tRNA and emphasizes its specialized function in initiating the translation process.

Simulation data further suggest that tRNA binding directly instigates conformational changes in the catalytic domain that weaken ligand-protein interactions, particularly in the initiator tRNA-bound complex (**Figure S30 and S40**). In multiple simulations, initiator tRNA engagement led to repositioning of the CP1 loop, thereby opening the ligand-binding site and destabilizing the Met-AMP ligand. Interestingly, this destabilization was not uniform across all simulations. The tRNA deviated from the active site (green trajectory) in one trajectory, which stabilized ligand

positioning. However, in two other simulations (blue and light brown), the 3′ CCA end persistently interacted with the CP1 domain, reshaping the pocket and weakening ligand retention. This ligand displacement was unique to the initiator tRNA complex and did not occur in the elongator complex, where the ligand remained stably associated. These findings suggest that the structural flexibility and distinct base-pairing at the acceptor stem of the initiator tRNA, including the unpaired C1:U73 base pair, may facilitate an interaction with Met-AMP on a shorter timescale, enhancing aminoacylation efficiency.

The interaction of tRNA with KMSKS loop region of MetRS also modulates ligand stability within the catalytic pocket. Particularly in the initiator complex, electrostatic pulling of the KMSKS loop induced by phosphate-backbone attraction leads to partial opening of the ligand-binding site, thereby weakening ligand-protein interactions and increasing the likelihood of ligand-tRNA contact. In contrast, the elongator complex maintains tight ligand coordination due to structural rigidity and reduced loop mobility.

Table S1. Nucleotide sequence alignment of tRNA sequences of *Mtb.*

Sequence name 1	Sequence name 2	BLASTN local alignment		EMBOSS global alignment	
		% identity	Query coverage	% identity	% similarity
$tRNA^{fMet}$	$tRNA^{Met}$	NA	NA	67.9	67.9

*NA – No significant similarity found.

Table S2. The average and standard deviation values of each property. The average of 3 replica averages and standard deviations (represents within replicates standard deviation) are provided.

S. No.	Property	Run	Average (Initiator complex)	Standard deviation (Initiator complex)	Average (Elongator complex)	Standard deviation (Elongator complex)
**Protein properties**						
1.	Protein RMSD (nm)	**Run1**	0.28	0.04	0.44	0.04
		**Run2**	0.37	0.04	0.42	0.06
		**Run3**	0.42	0.05	0.34	0.04
		**Average of Runs**	**0.36**	**0.04**	**0.40**	**0.05**
2.	Protein R_g (nm)	**Run1**	2.72	0.02	2.77	0.02
		**Run2**	2.76	0.02	2.75	0.03
		**Run3**	2.79	0.02	2.74	0.02
		**Average of Runs**	**2.76**	**0.02**	**2.75**	**0.02**
3.	Protein total SASA (nm^2)	**Run1**	238.0	4.8	238.7	3.4
		**Run2**	235.3	3.0	239.5	5.4
		**Run3**	243.1	3.6	245.4	3.2
		**Average of Runs**	**238.8**	**3.8**	**241.2**	**4.0**
4.	Protein hydrophobic SASA (nm^2)	**Run1**	61.5	1.4	61.9	1.3
		**Run2**	63.9	1.5	62.9	1.8
		**Run3**	62.5	1.6	63.0	1.4
		**Average of Runs**	**62.6**	**1.5**	**62.6**	**1.5**
5.	Protein hydrophilic SASA (nm^2)	**Run1**	177	4	177	3
		**Run2**	171	3	177	4
		**Run3**	181	3	182	3
		**Average of Runs**	**176**	**3**	**179**	**3**
6.	Protein RMSF (nm)	**Run1**	0.12	0.14	0.10	0.10
		**Run2**	0.10	0.05	0.10	0.08
		**Run3**	0.12	0.11	0.12	0.14
		**Average of Runs**	**0.11**	**0.10**	**0.11**	**0.11**
7.	% Turn	**Run1**	16.7	1.5	16.2	1.2
		**Run2**	16.8	1.5	16.4	1.4
		**Run3**	16.1	1.3	16.6	1.4
		**Average of Runs**	**16.5**	**1.4**	**16.4**	**1.3**
8.	% Beta sheet	**Run1**	13.99	0.62	13.78	0.54
		**Run2**	14.43	0.51	12.66	0.66
		**Run3**	14.37	0.76	11.50	0.63
		**Average of Runs**	**14.26**	**0.63**	**12.65**	**0.61**
9.	% Isolated beta bridge	**Run1**	0.71	0.26	0.91	0.19
		**Run2**	0.58	0.17	0.62	0.23
		**Run3**	0.59	0.18	1.01	0.29

		**Average of Runs**	**0.63**	**0.20**	**0.85**	**0.24**
10.	% α - helix	**Run1**	47.88	0.77	48.95	0.67
		**Run2**	49.02	0.52	48.84	0.71
		**Run3**	48.73	0.75	49.30	0.70
		**Average of Runs**	**48.54**	**0.68**	**49.03**	**0.69**
11.	% 3_{10}-helix	**Run1**	1.66	0.70	1.74	0.68
		**Run2**	1.60	0.64	1.50	0.63
		**Run3**	1.60	0.66	1.05	0.59
		**Average of Runs**	**1.62**	**0.67**	**1.43**	**0.63**
12.	% π-helix	**Run1**	0.01	0.07	0.09	0.28
		**Run2**	0.00	0.04	0.07	0.25
		**Run3**	0.0	0.1	0.0	0.1
		**Average of Runs**	**0.00**	**0.07**	**0.05**	**0.21**
13.	% Coil	**Run1**	19.1	1.2	18.3	0.9
		**Run2**	17.6	1.0	19.9	1.0
		**Run3**	18.6	1.1	20.5	1.1
		**Average of Runs**	**18.4**	**1.1**	**19.6**	**1.0**
**tRNA properties**						
14.	tRNA RMSD (nm) with respect to protein	**Run1**	5.7	2.4	1.8	0.3
		**Run2**	1.6	0.3	2.1	0.3
		**Run3**	3.1	0.9	2.7	0.8
		**Average of Runs**	**3.5**	**1.2**	**2.2**	**0.5**
15.	tRNA R_g (nm)	**Run1**	2.48	0.12	2.42	0.05
		**Run2**	2.32	0.04	2.39	0.04
		**Run3**	2.17	0.10	2.36	0.07
		**Average of Runs**	**2.32**	**0.09**	**2.39**	**0.05**
16.	tRNA SASA (nm^2)	**Run1**	135.1	3.6	138.5	2.3
		**Run2**	134.6	3.5	137.4	2.8
		**Run3**	135.1	3.7	137.8	3.4
		**Average of Runs**	**134.9**	**3.6**	**137.9**	**2.8**
17.	tRNA RMSF (nm)	**Run1**	0.41	0.19	0.23	0.07
		**Run2**	0.25	0.09	0.31	0.15
		**Run3**	0.33	0.21	0.29	0.12
		**Average of Runs**	**0.33**	**0.16**	**0.28**	**0.11**
**Ligand properties**						
18.	Ligand RMSD (nm) with respect to protein	**Run1**	0.7	0.2	1.0	0.3
		**Run2**	2.3	2.1	0.5	0.1
		**Run3**	4.5	2.1	0.5	0.3
		**Average of Runs**	**2.5**	**1.5**	**0.7**	**0.2**
19.	Ligand R_g (nm)	**Run1**	0.48	0.02	0.45	0.02
		**Run2**	0.43	0.04	0.48	0.02
		**Run3**	0.42	0.04	0.47	0.03

		**Average of Runs**	**0.44**	**0.03**	**0.47**	**0.02**
20.	Ligand SASA (nm^2)	**Run1**	7.05	0.24	6.89	0.25
		**Run2**	6.73	0.43	6.95	0.23
		**Run3**	6.66	0.41	6.92	0.30
		**Average of Runs**	**6.81**	**0.36**	**6.92**	**0.26**
21.	Ligand RMSF (nm)	**Run1**	0.19	0.07	0.18	0.07
		**Run2**	0.26	0.09	0.10	0.05
		**Run3**	0.25	0.08	0.19	0.08
		**Average of Runs**	**0.23**	**0.08**	**0.16**	**0.07**
**Intramolecular hydrogen bonds**						
22.	Intra-protein hydrogen bonds	**Run1**	334	21	316	19
		**Run2**	352	22	299	19
		**Run3**	327	21	306	19
		**Average of Runs**	**338**	**21**	**307**	**19**
23.	Intra-tRNA hydrogen bonds	**Run1**	101	11	118	12
		**Run2**	106	12	120	12
		**Run3**	105	12	109	12
		**Average of Runs**	**104**	**12**	**116**	**12**
**Hydrogen bond interactions with water**						
24.	Hydrogen bonds between protein and water	**Run1**	935	22	921	23
		**Run2**	924	22	943	26
		**Run3**	972	25	940	27
		**Average of Runs**	**944**	**23**	**935**	**25**
25.	Hydrogen bonds between tRNA and water	**Run1**	398	49	594	23
		**Run2**	602	27	608	20
		**Run3**	559	43	595	27
		**Average of Runs**	**520**	**40**	**599**	**23**
26.	Hydrogen bonds between ligand and water	**Run1**	11.2	2.5	10.3	2.5
		**Run2**	12.0	2.7	11.7	2.3
		**Run3**	11.9	3.1	9.4	2.3
		**Average of Runs**	**11.7**	**2.8**	**10.5**	**2.4**
**tRNA-protein interactions**						
27.	Hydrogen bonds between tRNA and protein	**Run1**	6	3	13	3
		**Run2**	11	3	9	3
		**Run3**	8	3	10	3
		**Average of Runs**	**8**	**3**	**11**	**3**
28.	Salt bridge between tRNA and protein	**Run1**	3.8	1.6	4.1	1.5
		**Run2**	4.6	1.1	5.1	1.6
		**Run3**	6.0	1.5	6.5	1.9
		**Average of Runs**	**4.8**	**1.4**	**5.2**	**1.7**

29.	π- π interactions between tRNA and protein	**Run1**	0.28	0.58	0.11	0.32
		**Run2**	0.37	0.51	0.05	0.21
		**Run3**	0.13	0.38	0.23	0.53
		**Average of Runs**	**0.26**	**0.49**	**0.13**	**0.35**
30.	π-cation interactions between tRNA and protein	**Run1**	0.42	0.67	0.31	0.49
		**Run2**	0.94	0.37	0.01	0.11
		**Run3**	0.11	0.32	0.11	0.32
		**Average of Runs**	**0.49**	**0.45**	**0.14**	**0.31**
31.	π-anion interactions between tRNA and protein	**Run1**	0.03	0.18	0.12	0.33
		**Run2**	0.05	0.22	0.06	0.24
		**Run3**	0.00	0.00	0.20	0.40
		**Average of Runs**	**0.03**	**0.13**	**0.13**	**0.32**
32.	ΔG_{bind} (kcal/mole) for tRNA and protein binding	**Run1**	-2	39	-63	14
		**Run2**	-9	11	-31	16
		**Run3**	-29	14	-67	19
		**Average of Runs**	**-13**	**21**	**-54**	**16**
**tRNA-ligand interactions**						
33.	Hydrogen bonds between tRNA and ligand	**Run1**	0.0	0.0	1.4	1.0
		**Run2**	0.3	0.8	0.4	0.5
		**Run3**	0.3	0.9	0.8	1.1
		**Average of Runs**	**0.2**	**0.6**	**0.9**	**0.9**
34.	Salt bridge between tRNA and ligand	**Run1**	0.0	0.0	0.6	0.8
		**Run2**	0.2	0.6	0.0	0.0
		**Run3**	0.2	0.8	0.7	1.0
		**Average of Runs**	**0.1**	**0.5**	**0.4**	**0.6**
35.	π- π interactions between tRNA and ligand	**Run1**	0.00	0.00	0.03	0.17
		**Run2**	0.00	0.04	0.00	0.00
		**Run3**	0.06	0.24	0.00	0.04
		**Average of Runs**	**0.02**	**0.09**	**0.01**	**0.07**
36.	π-cation interactions between tRNA and ligand	**Run1**	0.00	0.00	0.09	0.28
		**Run2**	0.00	0.00	0.00	0.00
		**Run3**	0.01	0.12	0.00	0.05
		**Average of Runs**	**0.00**	**0.04**	**0.03**	**0.11**
37.	π-anion interactions between tRNA and ligand	**Run1**	0.00	0.00	0.04	0.19
		**Run2**	0.00	0.05	0.00	0.00
		**Run3**	0.02	0.13	0.00	0.00
		**Average of Runs**	**0.01**	**0.06**	**0.01**	**0.06**
**Protein-ligand interactions**						
38.	Hydrogen bonds between protein and ligand	**Run1**	1.9	1.4	1.6	1.1
		**Run2**	0.7	1.0	2.3	1.5
		**Run3**	0.4	0.9	2.8	2.1
		**Average of Runs**	**1.0**	**1.1**	**2.2**	**1.6**

39.	Salt bridge between protein and ligand	**Run1**	1.6	0.7	0.9	0.8
		**Run2**	0.5	0.7	1.0	0.3
		**Run3**	0.2	0.4	0.7	0.7
		**Average of Runs**	**0.7**	**0.6**	**0.9**	**0.6**
40.	π- π interactions between protein and ligand	**Run1**	0.05	0.23	0.00	0.00
		**Run2**	0.04	0.19	1.43	0.53
		**Run3**	0.03	0.17	0.48	0.58
		**Average of Runs**	**0.04**	**0.20**	**0.64**	**0.37**
41.	π-cation interactions between protein and ligand	**Run1**	0.15	0.36	0.03	0.18
		**Run2**	0.03	0.17	0.01	0.09
		**Run3**	0.00	0.06	0.05	0.24
		**Average of Runs**	**0.06**	**0.20**	**0.03**	**0.17**
42.	π-anion interactions between protein and ligand	**Run1**	0.37	0.48	0.02	0.19
		**Run2**	0.01	0.11	0.01	0.08
		**Run3**	0.02	0.14	0.02	0.14
		**Average of Runs**	**0.13**	**0.24**	**0.02**	**0.14**
43.	ΔG_{bind} (kcal/mole) for protein and ligand binding	**Run1**	-30	5	-21	4
		**Run2**	-8	9	-35	5
		**Run3**	0	0	-28	9
		**Average of Runs**	**-13**	**5**	**-28**	**6**
**Interactions with Mg^{2+} ion**						
44.	Electrostatic interactions between Mg^{2+} and protein	**Run1**	0.01	0.11	0.01	0.08
		**Run2**	0.02	0.13	0.00	0.00
		**Run3**	0.00	0.00	0.00	0.00
		**Average of Runs**	**0.01**	**0.08**	**0.00**	**0.03**
45.	Electrostatic interactions between Mg^{2+} and tRNA	**Run1**	0.00	0.00	0.00	0.00
		**Run2**	0.00	0.00	2.02	0.17
		**Run3**	0.74	0.63	0.02	0.15
		**Average of Runs**	**0.25**	**0.21**	**0.68**	**0.11**
46.	Electrostatic interactions between Mg^{2+} and ligand	**Run1**	0.0	0.0	0.0	0.0
		**Run2**	0.0	0.0	0.0	0.0
		**Run3**	0.0	0.0	0.0	0.0
		**Average of Runs**	**0.0**	**0.0**	**0.0**	**0.0**
47.	Number of contacts between Mg^{2+} and protein	**Run1**	29.3	4.0	29.6	2.7
		**Run2**	35.8	2.5	33.6	3.9
		**Run3**	35.1	2.2	37.8	3.3
		**Average of Runs**	**33.4**	**2.9**	**33.7**	**3.3**
48.	Number of contacts between Mg^{2+} and tRNA	**Run1**	0	0	0	0
		**Run2**	0	0	7.22	1.21
		**Run3**	5.12	1.49	0.04	0.22
		**Average of Runs**	**1.71**	**0.50**	**2.42**	**0.48**
49.	Number of contacts between Mg^{2+} and ligand	**Run1**	0	0	0	0
		**Run2**	0	0	0	0

		**Run3**	0	0	1.8	2.0
		**Average of Runs**	**0**	**0**	**0.6**	**0.7**
**Initiator tRNA specific features**						
50.	Hydrogen bonds between C1 and U73	**Run1**	0.06	0.23		
		**Run2**	0.00	0.00		
		**Run3**	0.00	0.06		
		**Average of Runs**	**0.02**	**0.10**		
51.	Hydrogen bonds between A11 and U25	**Run1**	1.82	0.61		
		**Run2**	1.68	0.54		
		**Run3**	1.79	0.58		
		**Average of Runs**	**1.76**	**0.58**		
**Elongator tRNA specific feature**						
52.	Hydrogen bonds between G1 and U73	**Run1**			1.24	0.66
		**Run2**			1.00	0.72
		**Run3**			0.97	0.64
		**Average of Runs**			**1.07**	**0.67**
**tRNA anticodon ($_{35}$CAU$_{37}$) interactions with protein**						
53.	Hydrogen bonds between C35 and protein	**Run1**	0.49	1.02	0.31	0.55
		**Run2**	2.03	0.57	0.00	0.04
		**Run3**	2.19	1.11	0.11	0.33
		**Average of Runs**	**1.57**	**0.90**	**0.14**	**0.31**
54.	Hydrogen bonds between A36 and protein	**Run1**	0.16	0.42	1.08	1.19
		**Run2**	1.54	0.97	0.09	0.31
		**Run3**	0.06	0.27	0.06	0.25
		**Average of Runs**	**0.59**	**0.55**	**0.41**	**0.58**
55.	Hydrogen bonds between U37 and protein	**Run1**	0.03	0.17	0.67	0.96
		**Run2**	0.66	0.53	0.79	0.66
		**Run3**	0.07	0.26	0.21	0.46
		**Average of Runs**	**0.25**	**0.32**	**0.56**	**0.69**
**tRNA interactions with conserved residues of protein's ABD**						
56.	Hydrogen bonds between Asn357 and tRNA	**Run1**	0.31	0.71	0.58	0.62
		**Run2**	2.01	0.54	0.70	0.82
		**Run3**	0.50	0.69	0.01	0.08
		**Average of Runs**	**0.94**	**0.65**	**0.43**	**0.51**
57.	Hydrogen bonds between Arg361 and tRNA	**Run1**	0.22	0.48	0.50	0.74
		**Run2**	0.01	0.09	0.68	0.73
		**Run3**	0.83	0.82	0.80	0.89
		**Average of Runs**	**0.35**	**0.46**	**0.66**	**0.79**
58.	Hydrogen bonds between Trp431 and tRNA	**Run1**	0.09	0.34	0.12	0.32
		**Run2**	0.0	0.13	0.03	0.20
		**Run3**	0.03	0.17	0.10	0.35

		**Average of Runs**	**0.04**	**0.21**	**0.08**	**0.29**
**tRNA CCA end interactions with protein**						
59.	Hydrogen bonds between C75 and protein	**Run1**	0.08	0.35	2.05	0.80
		**Run2**	0.00	0.00	0.57	0.92
		**Run3**	0.13	0.39	1.47	1.14
		**Average of Runs**	**0.07**	**0.25**	**1.36**	**0.95**
60.	Hydrogen bonds between C76 and protein	**Run1**	0.28	0.52	0.69	0.85
		**Run2**	0.51	0.96	0.80	1.11
		**Run3**	0.16	0.42	1.22	1.07
		**Average of Runs**	**0.32**	**0.63**	**0.90**	**1.01**
61.	Hydrogen bonds between A77 and protein	**Run1**	0.53	0.75	2.07	1.80
		**Run2**	1.16	1.33	0.36	0.66
		**Run3**	1.54	0.79	0.87	0.89
		**Average of Runs**	**1.08**	**0.96**	**1.10**	**1.12**
**tRNA CCA end interactions with ligand (Met-AMP)**						
62.	Hydrogen bonds between C75 and ligand	**Run1**	0.00	0.00	0.37	0.61
		**Run2**	0.00	0.00	0.00	0.00
		**Run3**	0.00	0.00	0.58	0.89
		**Average of Runs**	**0.00**	**0.00**	**0.32**	**0.50**
63.	Hydrogen bonds between C76 and ligand	**Run1**	0.00	0.00	0.23	0.58
		**Run2**	0.00	0.00	0.00	0.00
		**Run3**	0.00	0.00	0.03	0.18
		**Average of Runs**	**0.00**	**0.00**	**0.09**	**0.25**
64	Hydrogen bonds between A77 and ligand	**Run1**	0.00	0.00	0.00	0.00
		**Run2**	0.00	0.00	0.00	0.00
		**Run3**	0.00	0.00	0.20	0.44
		**Average of Runs**	**0.00**	**0.00**	**0.07**	**0.15**

Table S3. The t-test results for comparison of protein, tRNA, and ligand properties between the initiator and elongator complexes. The total data points for each property were divided into blocks. The block size was calculated based on the standard error of the block means, known as the block standard error (BSE). The resulting number of blocks was used to determine the significant differences (p-values) between the means of the properties of the initiator and elongator complexes. The mean of the property represented in the table is the average of the block means.

Property	Total points in each dataset	Block size	Total number of blocks	p-value (paired, two-tail)	p < 0.05	Mean of initiator property	BSE of initiator property	Mean of elongator property	BSE of elongator property
Protein RMSD	3003	50	60	4.32e-03	Yes	0.36	0.01	0.40	0.01
Protein R_g	3003	45	66	6.77e-01	No	2.76	0.00	2.75	0.00
Protein total SASA	3003	45	66	3.35e-05	Yes	238.79	0.53	241.19	0.51
% Turn	1503	40	36	2.00e-01	No	16.58	0.12	16.37	0.08
% Beta sheet	1503	45	33	4.31e-09	Yes	14.27	0.07	12.65	0.17
% Isolated bridge	1503	50	30	1.65e-06	Yes	0.62	0.02	0.85	0.04
% α-helix	1503	45	33	5.13e-05	Yes	48.54	0.09	49.03	0.05
% 3_{10} -helix	1503	45	33	4.26e-03	Yes	1.62	0.03	1.43	0.05
% π-helix	1503	25	60	4.43e-05	Yes	0.01	0.00	0.06	0.01
% Coil	1503	45	33	2.12e-04	Yes	18.39	0.16	19.60	0.18
tRNA RMSD	3003	55	54	2.14e-04	Yes	3.44	0.30	2.18	0.08
tRNA R_g	3003	50	60	1.06e-04	Yes	2.32	0.02	2.39	0.01
tRNA SASA	3003	50	60	3.49e-12	Yes	134.93	0.35	137.91	0.29
Ligand RMSD	3003	55	54	3.05e-07	Yes	2.48	0.29	0.65	0.05
Ligand R_g	3003	50	60	1.04e-03	Yes	0.44	0.00	0.47	0.00
Ligand SASA	3003	45	66	1.95e-03	Yes	6.81	0.03	6.92	0.02
tRNA-protein hydrogen bonds	1503	45	33	3.69e-03	Yes	8.14	0.43	10.66	0.40
tRNA-protein salt bridge	1500	45	33	2.54e-02	Yes	4.79	0.20	5.22	0.24

**ΔG_{bind} protein-tRNA**	150	25	6	1.11e-02	Yes	-13	8	-54	7
**tRNA-ligand hydrogen bonds**	1503	35	42	2.21e-04	Yes	0.22	0.08	0.84	0.11
**tRNA-ligand salt bridge**	1500	35	42	2.10e-02	Yes	0.15	0.05	0.43	0.09
**Protein-ligand hydrogen bonds**	1503	45	33	2.54e-04	Yes	0.99	0.14	2.21	0.22
**Protein-ligand salt bridge**	1500	45	33	3.11e-01	No	0.74	0.12	0.88	0.08
**ΔG_{bind} protein-ligand**	150	40	3	2.97e-01	No	-12	7	-28	3

Table S4. The t-test results for comparison of protein, tRNA, and ligand properties between the initiator and elongator complex at fixed block size. The total data points for each property were divided into a fixed block size of 100. The resulting number of blocks was used to determine the significant differences (p-values) between the means of the properties of the initiator and elongator complexes. The mean of the property represented in the table is the average of the block means.

Property	Total points in each dataset	Block size	Total number of blocks	p-value (paired, two-tail)	$p < 0.05$	Mean of initiator property	BSE of initiator property	Mean of elongator property	BSE of elongator property
**Protein RMSD**	3003	100	30	4.33e-02	Yes	0.36	0.01	0.40	0.01
**Protein R_g**	3003	100	30	7.89e-01	No	2.76	0.01	2.75	0.00
**Protein total SASA**	3003	100	30	2.64e-03	Yes	238.80	0.76	241.19	0.70
**% Turn**	1503	100	15	5.45e-01	No	16.54	0.17	16.39	0.09
**% Beta sheet**	1503	100	15	1.04e-04	Yes	14.27	0.09	12.64	0.24
**% Isolated bridge**	1503	100	15	7.23e-04	Yes	0.62	0.03	0.85	0.05
**% α-helix**	1503	100	15	6.11e-03	Yes	48.54	0.13	49.03	0.07
**% 3_{10} -helix**	1503	100	15	4.14e-02	Yes	1.62	0.03	1.43	0.08
**% π-helix**	1503	100	15	1.09e-02	Yes	0.01	0.00	0.06	0.01
**% Coil**	1503	100	15	1.38e-02	Yes	18.40	0.23	19.60	0.25
**tRNA RMSD**	3003	100	30	5.66e-03	Yes	3.45	0.40	2.18	0.10
**tRNA R_g**	3003	100	30	5.77e-03	Yes	2.32	0.03	2.39	0.01
**tRNA SASA**	3003	100	30	1.19e-07	Yes	134.93	0.45	137.91	0.37
**Ligand RMSD**	3003	100	30	1.15e-04	Yes	2.48	0.38	0.66	0.06
**Ligand R_g**	3003	100	30	1.75e-02	Yes	0.44	0.01	0.47	0.00
**Ligand SASA**	3003	100	30	2.91e-02	Yes	6.81	0.04	6.92	0.02
**tRNA-protein hydrogen bonds**	1503	100	15	4.83e-02	Yes	8.15	0.61	10.66	0.55
**tRNA-protein salt bridge**	1500	100	15	7.29e-02	No	4.80	0.27	5.22	0.34

**ΔG_{bind} protein-tRNA**	150	100	0	NA	No	NA	NA	NA	NA
**tRNA-ligand hydrogen bonds**	1503	100	15	1.77e-02	Yes	0.21	0.10	0.86	0.16
**tRNA-ligand salt bridge**	1500	100	15	1.30e-01	No	0.14	0.07	0.44	0.14
**Protein-ligand hydrogen bonds**	1503	100	15	1.40e-02	Yes	1.00	0.20	2.22	0.31
**Protein-ligand salt bridge**	1500	100	15	5.00e-01	No	0.74	0.17	0.88	0.09
**ΔG_{bind} protein-ligand**	150	100	0	NA	No	NA	NA	NA	NA

NA- not applicable in this case

Table S5. The t-test results for comparison of protein, tRNA, and ligand properties between the initiator (hairpin acceptor stem) and elongator complex (hairpin acceptor stem). The total data points for each property were divided into blocks. The block size was calculated based on the standard error of the block means, known as the block standard error (BSE). The resulting number of blocks was used to determine the significant differences (p-values) between the means of the properties of the initiator and elongator complexes. The mean of the property represented in the table is the average of the block means.

Property	Total points in each dataset	Block size	Total number of blocks	p-value (paired, two-tail)	$p < 0.05$	Mean of initiator property	BSE of initiator property	Mean of elongator property	BSE of elongator property
**Protein RMSD**	3003	50	60	6.00e-01	No	0.39	0.01	0.40	0.01
**Protein R_g**	3003	45	66	4.59e-03	Yes	2.77	0.00	2.75	0.00
**Protein total SASA**	3003	45	66	5.45e-01	No	240.71	0.54	241.19	0.51
**tRNA RMSD**	3003	50	60	7.31e-20	Yes	4.02	0.19	2.18	0.08
**tRNA R_g**	3003	45	66	2.74e-13	Yes	2.31	0.01	2.39	0.01
**tRNA SASA**	3003	50	60	5.71e-06	Yes	135.00	0.62	137.91	0.29
**Ligand RMSD**	3003	55	54	6.05e-16	Yes	3.87	0.30	0.65	0.05
**Ligand R_g**	300	50	60	3.28e-17	Yes	0.42	0.00	0.47	0.00
**Ligand SASA**	3003	45	66	2.48e-13	Yes	6.70	0.02	6.92	0.02
**ΔG_{bind} protein-tRNA**	150	35	3	1.42e-02	Yes	-34	7	-53	9
**ΔG_{bind} protein-ligand**	150	50	3	6.56e-03	Yes	-3	2	-28	3

Table S6. The t-test results for comparison of protein, tRNA, and ligand properties between the initiator (hairpin acceptor stem) and elongator complex (hairpin acceptor stem) at fixed block size. The total data points for each property were divided into a fixed block size of 100. The resulting number of blocks was used to determine the significant differences (p-values) between the means of the properties of the initiator and elongator complexes. The mean of the property represented in the table is the average of the block means.

Property	Total points in each dataset	Block size	Total number of blocks	p-value (paired, two-tail)	$p < 0.05$	Mean of initiator property	BSE of initiator property	Mean of elongator property	BSE of elongator property
**Protein RMSD**	3003	100	30	7.07e-01	No	0.39	0.01	0.40	0.01
**Protein R_g**	3003	100	30	5.12e-02	No	2.77	0.01	2.75	0.00
**Protein total SASA**	3003	100	30	6.99e-01	No	240.74	0.78	241.19	0.70
**tRNA RMSD**	3003	100	30	1.22e-10	Yes	4.02	0.26	2.18	0.10
**tRNA R_g**	3003	100	30	6.41e-07	Yes	2.31	0.01	2.39	0.01
**tRNA SASA**	3003	100	30	1.18e-03	Yes	135.00	0.86	137.91	0.37
**Ligand RMSD**	3003	100	30	2.27e-10	Yes	3.88	0.37	0.66	0.06
**Ligand R_g**	3003	100	30	3.86e-10	Yes	0.42	0.00	0.47	0.00
**Ligand SASA**	3003	100	30	1.19e-07	Yes	6.70	0.03	6.92	0.02
**ΔG_{bind} protein-tRNA**	150	100	0	NA	No	NA	NA	NA	NA
**ΔG_{bind} protein-ligand**	150	100	0	NA	No	NA	NA	NA	NA

NA- not applicable in this case

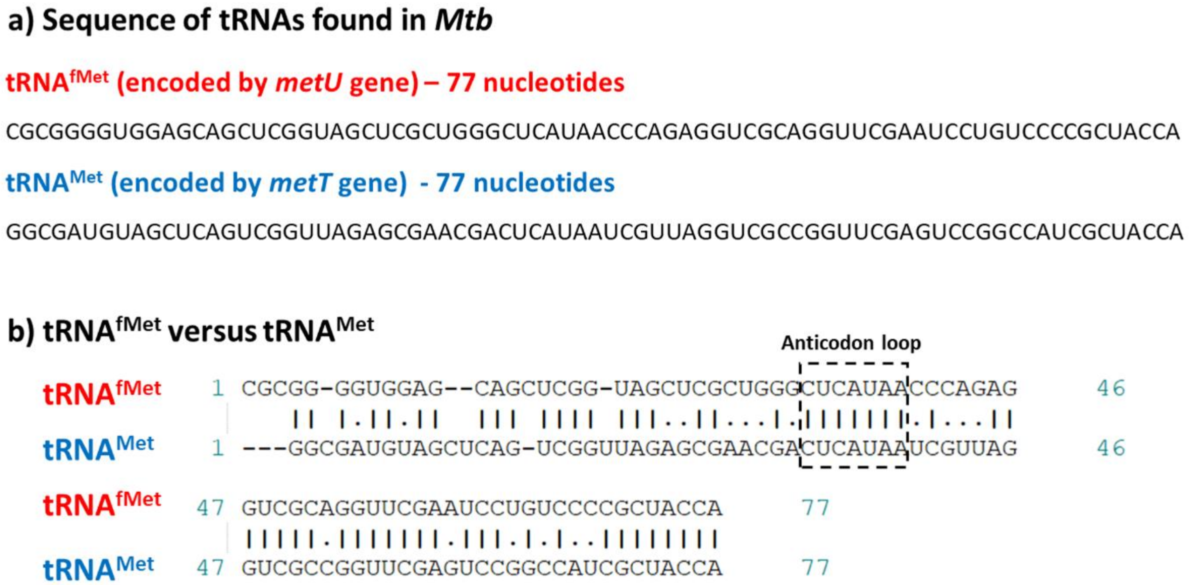

Figure S1. Nucleotide sequence alignment of tRNAs. (a) Sequences of tRNAs (tRNA^{fMet} and tRNA^{Met}) containing CAU anticodon found in *Mtb.* **(b)** Sequence alignment of tRNA^{fMet} versus tRNA^{Met}.

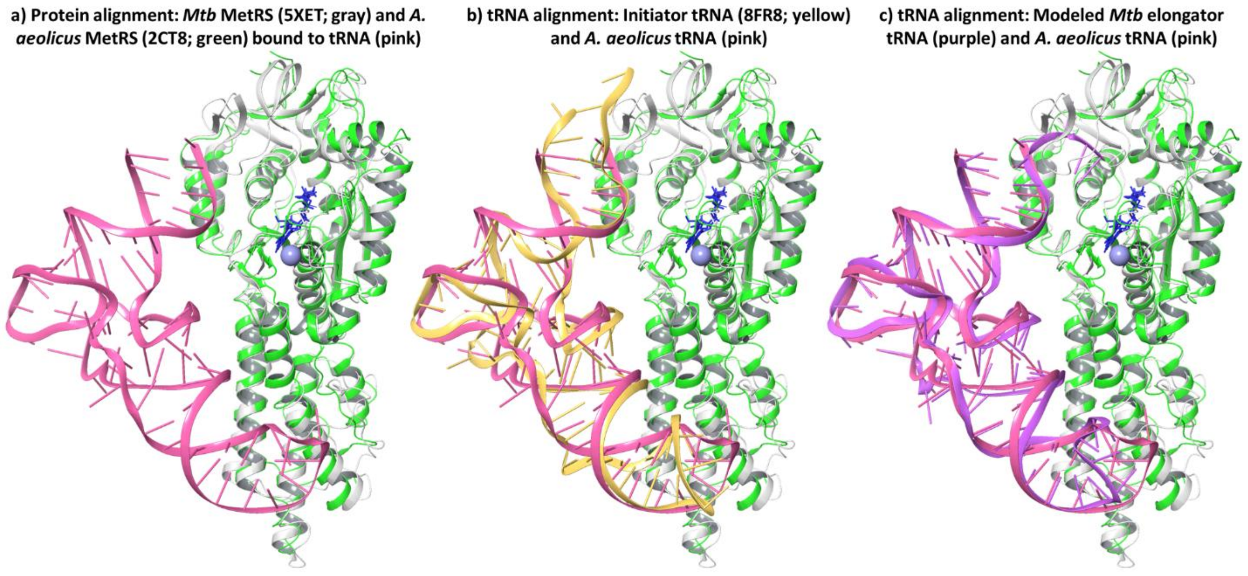

Figure S2. Steps involved in tRNA-complex building. (a) In first step, we aligned MetRS protein of *Mtb* (5XET; gray) with *A. aeolicus* (2CT8; green) bound to tRNA (pink). RMSD of MetRS protein (backbone) between *Mtb* (5XET) and *A. aeolicus* (2CT8) structures was low (i.e., 1.65 Å), suggesting high structural similarity between the two. Next step is alignment of tRNAs where we aligned **(b)** *M. smegmatis* initiator tRNA (8FR8; yellow) and *A. aeolicus* tRNA (2CT8; pink) to build initiator-tRNA complex and **(c)** modeled *Mtb* elongator tRNA (purple) with *A. aeolicus* tRNA (2CT8; pink) to build elongator-tRNA complex.

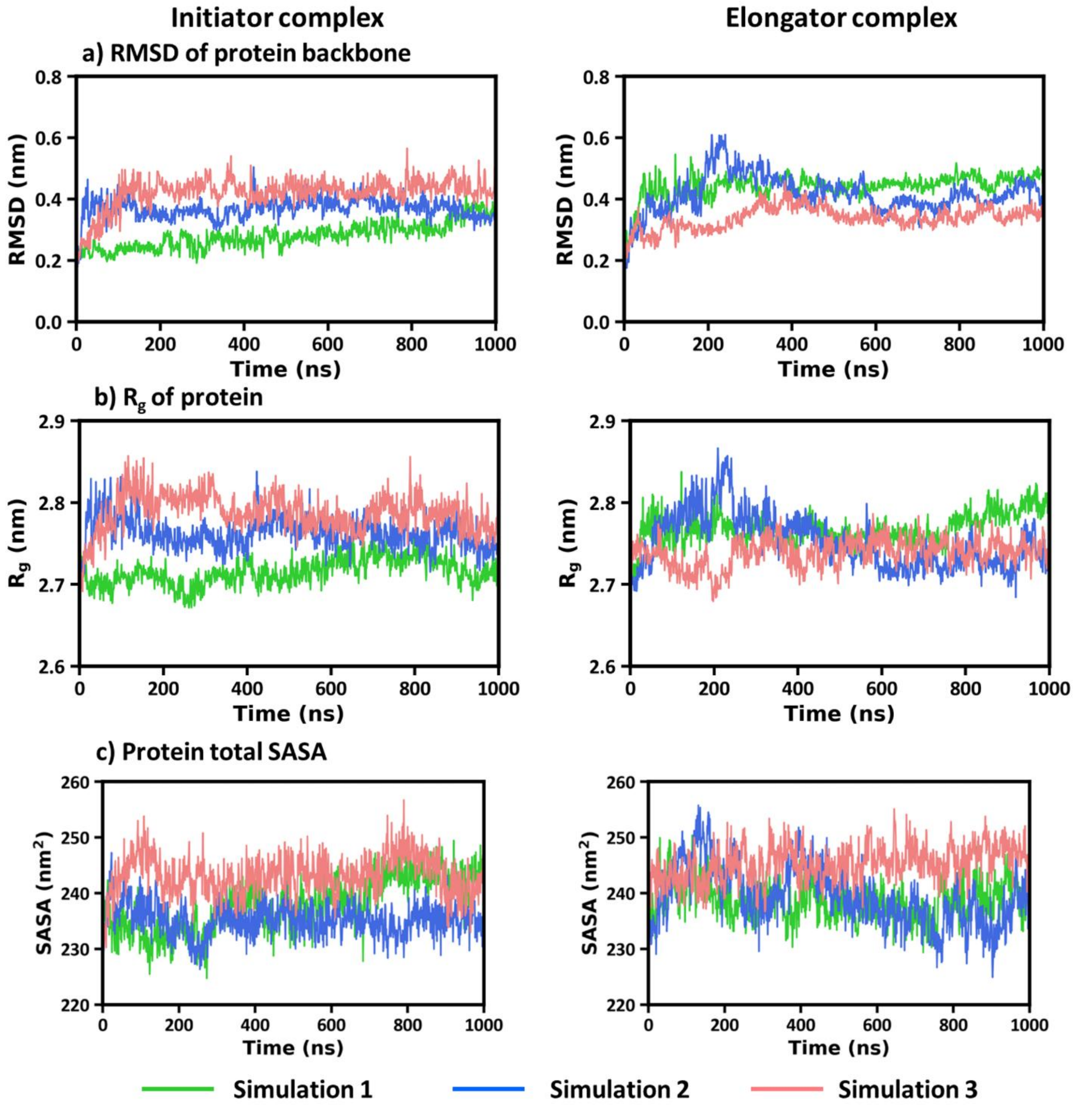

Figure S3. The simulated properties of MetRS protein. (a) The RMSD, **(b)** R_g, **(c)** total SASA of protein over three simulations (green, blue, peach). The representative colors for three simulations used in this figure are consistently followed in all subsequent plots.

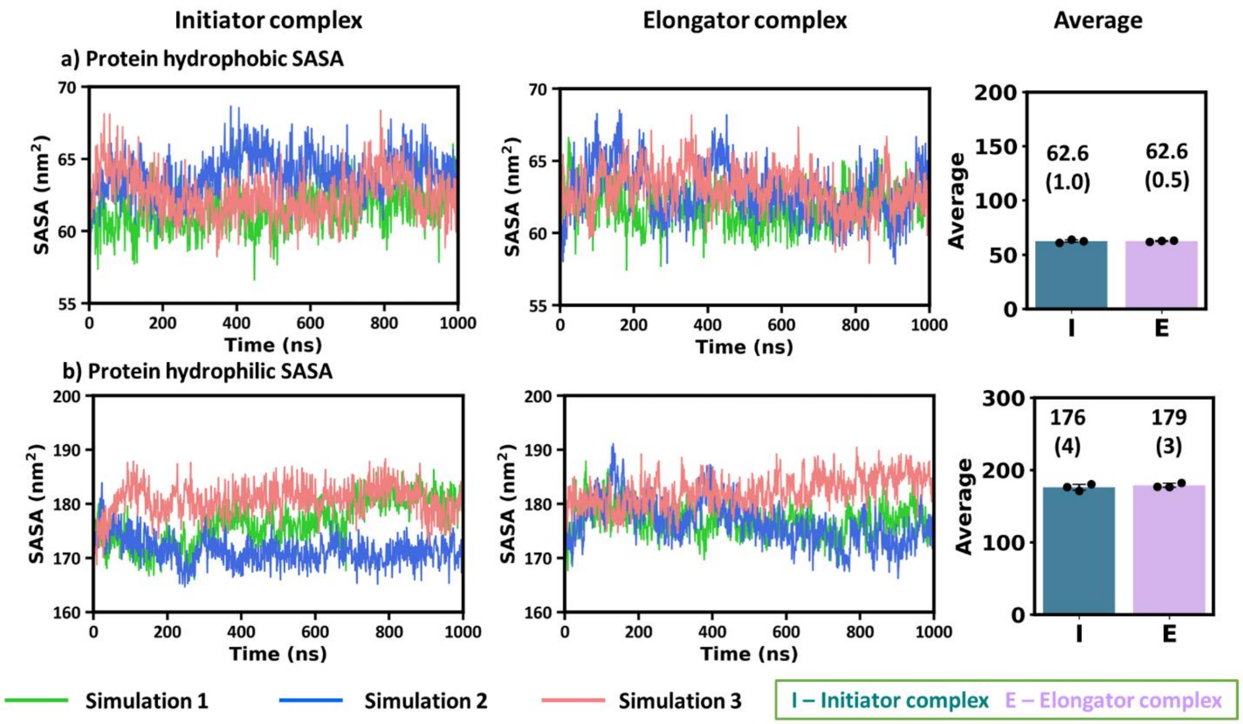

Figure S4. Hydrophobic and hydrophilic solvent-accessible surface area (SASA) analysis of *Mtb* MetRS protein. (a) Hydrophobic SASA and **(b)** hydrophilic SASA of protein over time for three simulations. Bar plots show the average and standard deviation (value in bracket; error bar) of hydrophobic and hydrophilic SASA across the three simulations.

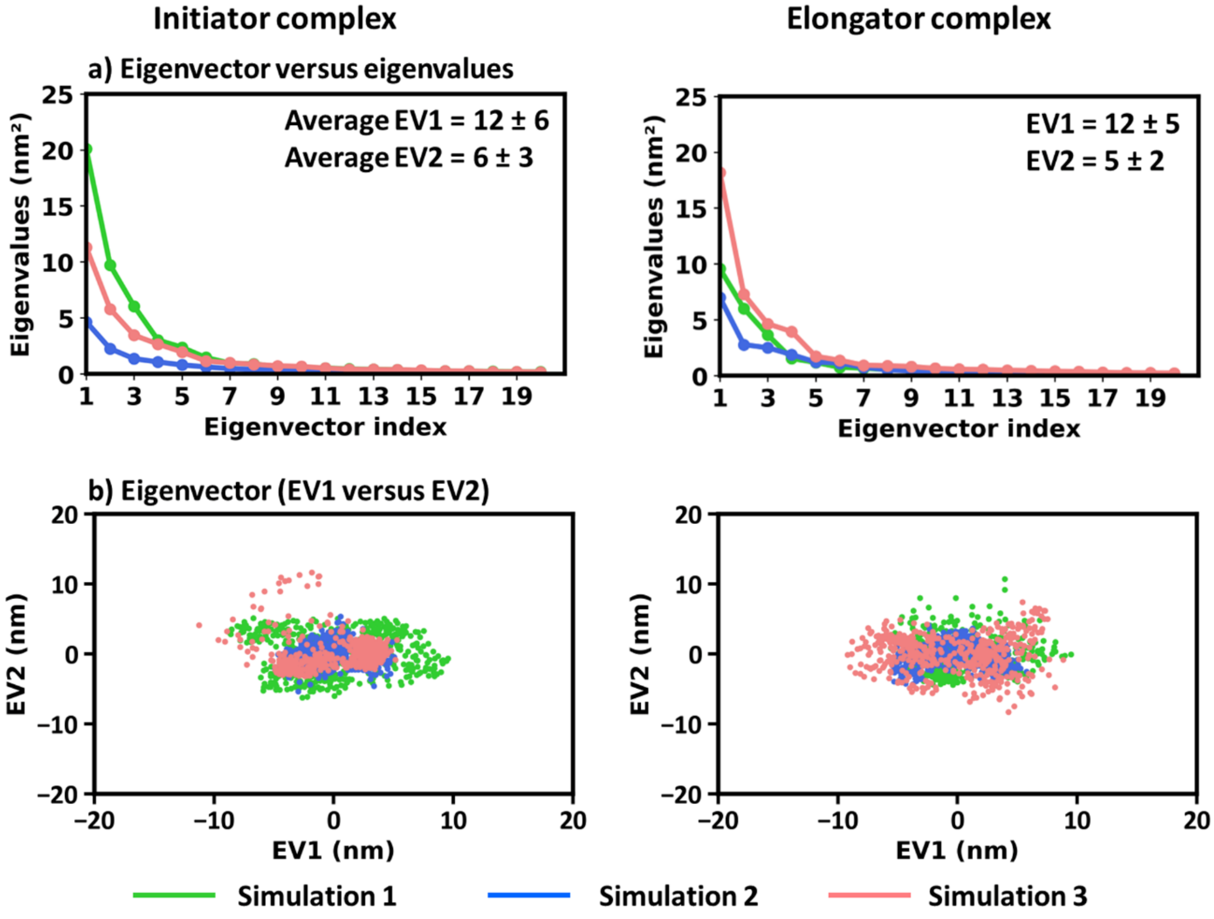

Figure S5. Free energy landscape principal component analysis (FEL-PCA) of protein. (a) First 20 eigenvectors (EV) with their eigenvalues. Average and standard deviation of eigenvalues for EV1 and EV2 over three simulations are provided. **(b)** EV1 versus EV2 projection plot for protein in initiator and elongator complex.

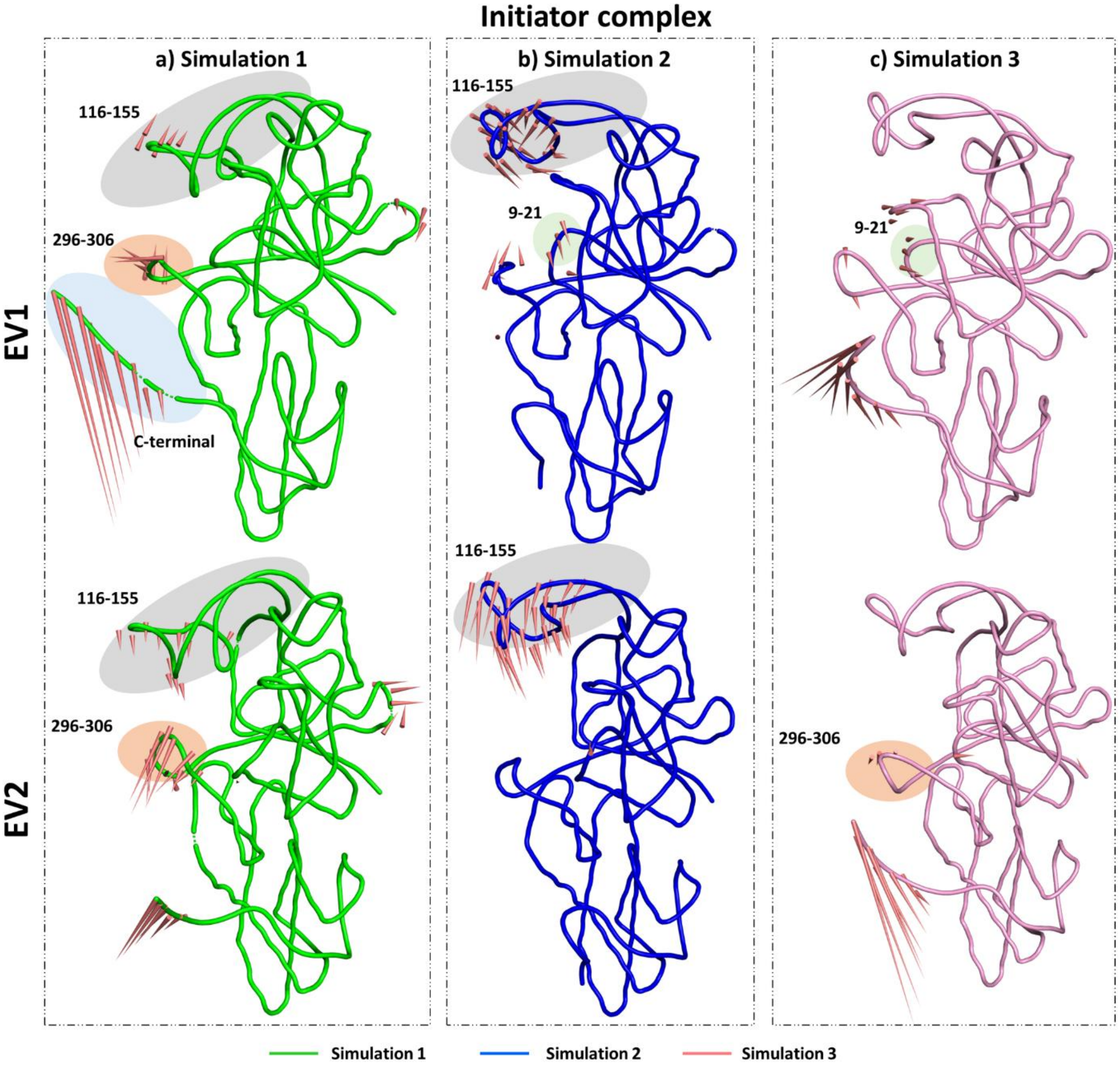

Figure S6. Mobility of protein in initiator complex. (a) – (c) Porcupine plots for first principal component (EV1) and second principal component (EV2) obtained from three simulations (represented in green, blue, peach color) of protein. The porcupine cones attached to average position of each backbone atom points towards direction of motion described by EV1 and EV2.

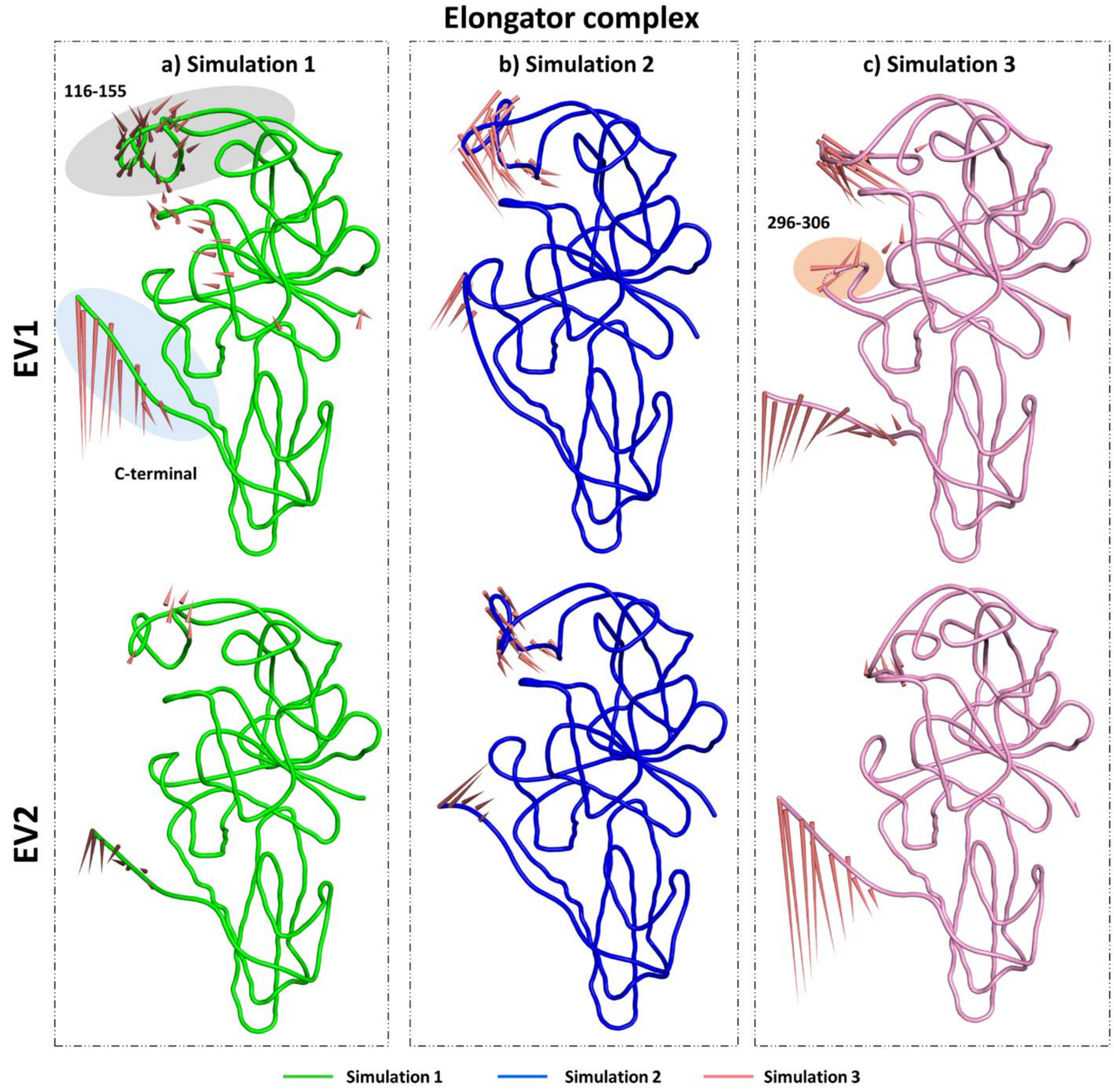

Figure S7. Mobility of protein in elongator complex. (a) – (c) Porcupine plots for first principal component (EV1) and second principal component (EV2) obtained from three simulations (represented in green, blue, peach color) of protein. The porcupine cones attached to average position of each backbone atom points towards direction of motion described by EV1 and EV2.

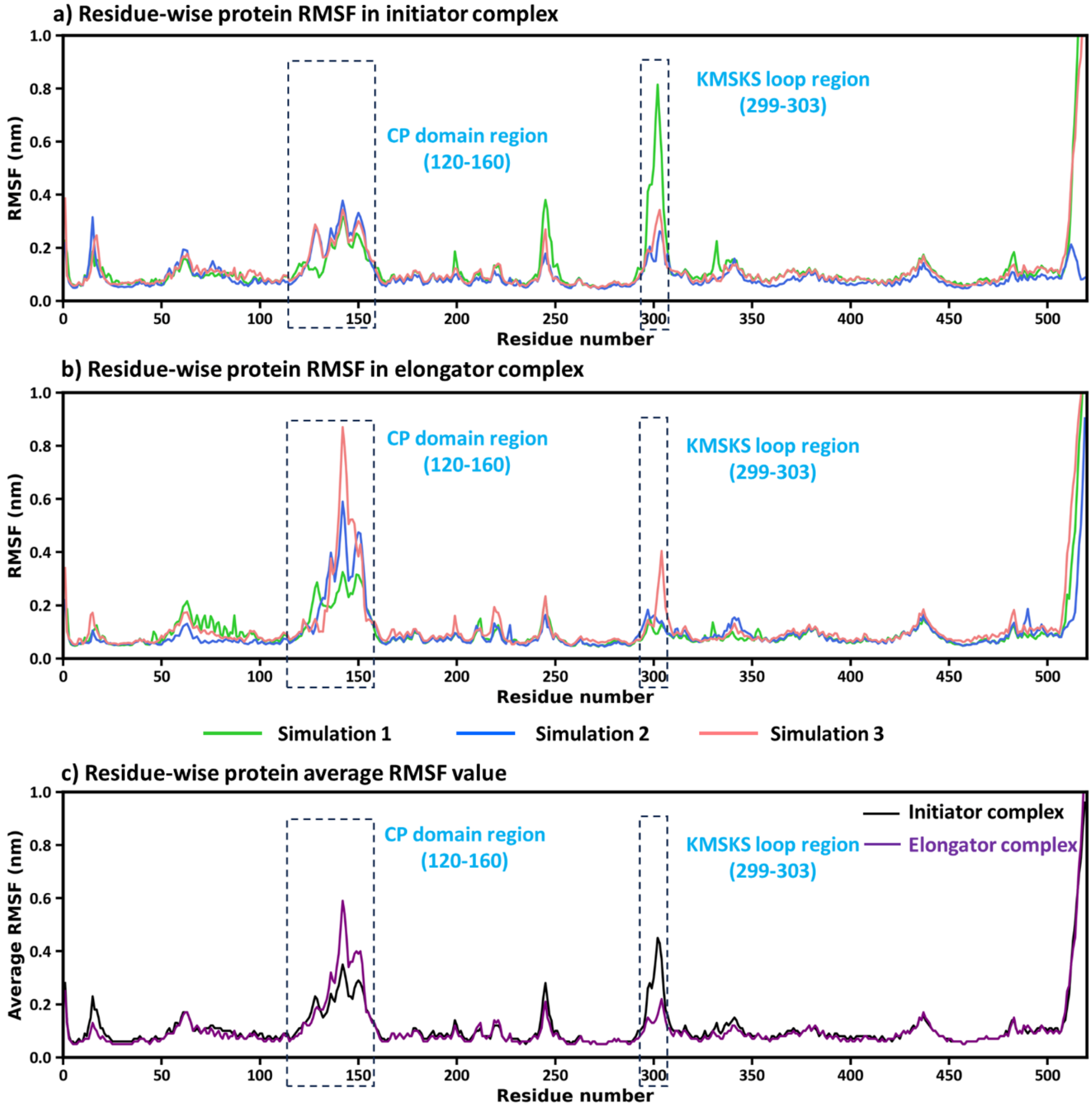

Figure S8. Residue-wise RMSF of MetRS protein in (a) initiator and **(b)** elongator tRNA-bound complexes across three simulations. **(c)** Average RMSF comparison highlights flexible regions, notably the CP domain (residues 120-160) and the KMSKS loop (299-303), showing differential mobility between complexes.

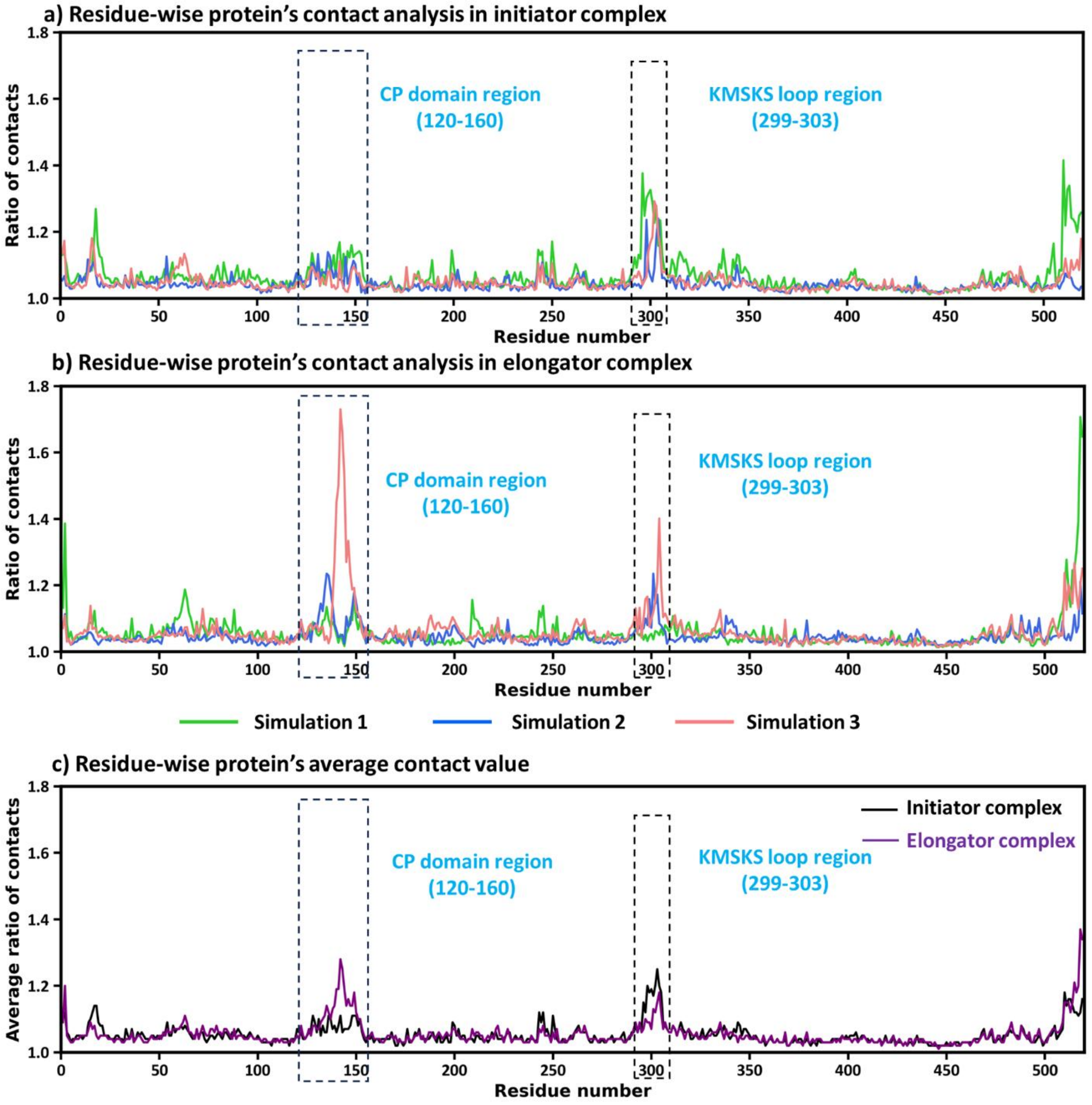

Figure S9. Residue-wise analysis of contacts in MetRS upon tRNA binding for (a) initiator and **(b)** elongator complexes over three simulations. Here "contacts" means count of number of different atomic contacts formed by each protein residue (x-axis) with atoms of other protein residues during simulations. "Ratio of contacts" is the total number of contacts divided by their mean value. **(c)** Average contact ratio comparison reveals enhanced contact formation in the CP domain (residues 120-160) and KMSKS loop (299-303).

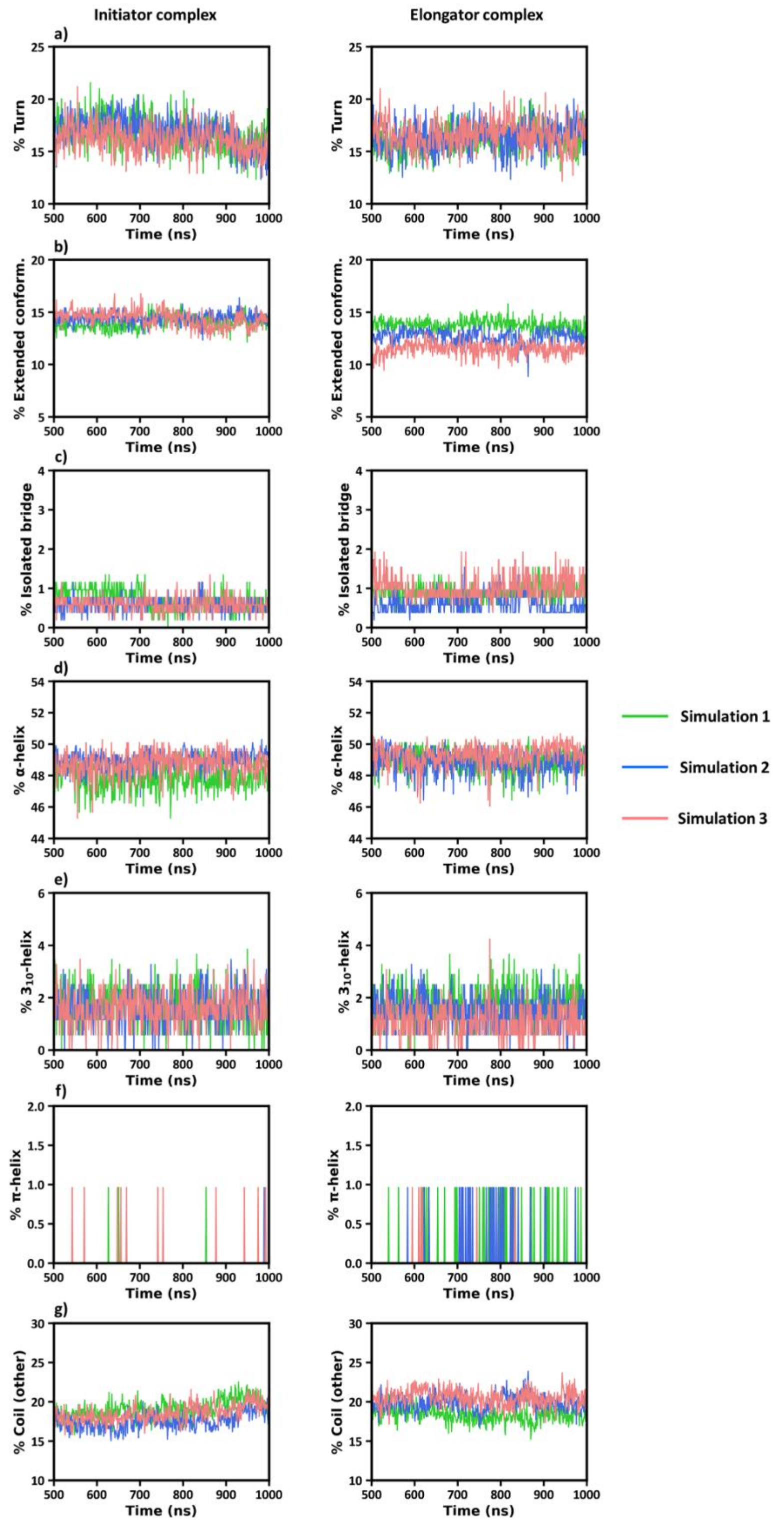

Figure S10. Protein secondary structure elements. (a) % Turn, **(b)** % Extended conformation or β-sheet, **(c)** % isolated bridge, **(d)** % α-helix, **(e)** % 3_{10}-helix, **(f)** % π-helix and **(g)** % coil (other) in initiator (left) and elongator (right) complexes over 1 μs simulations.

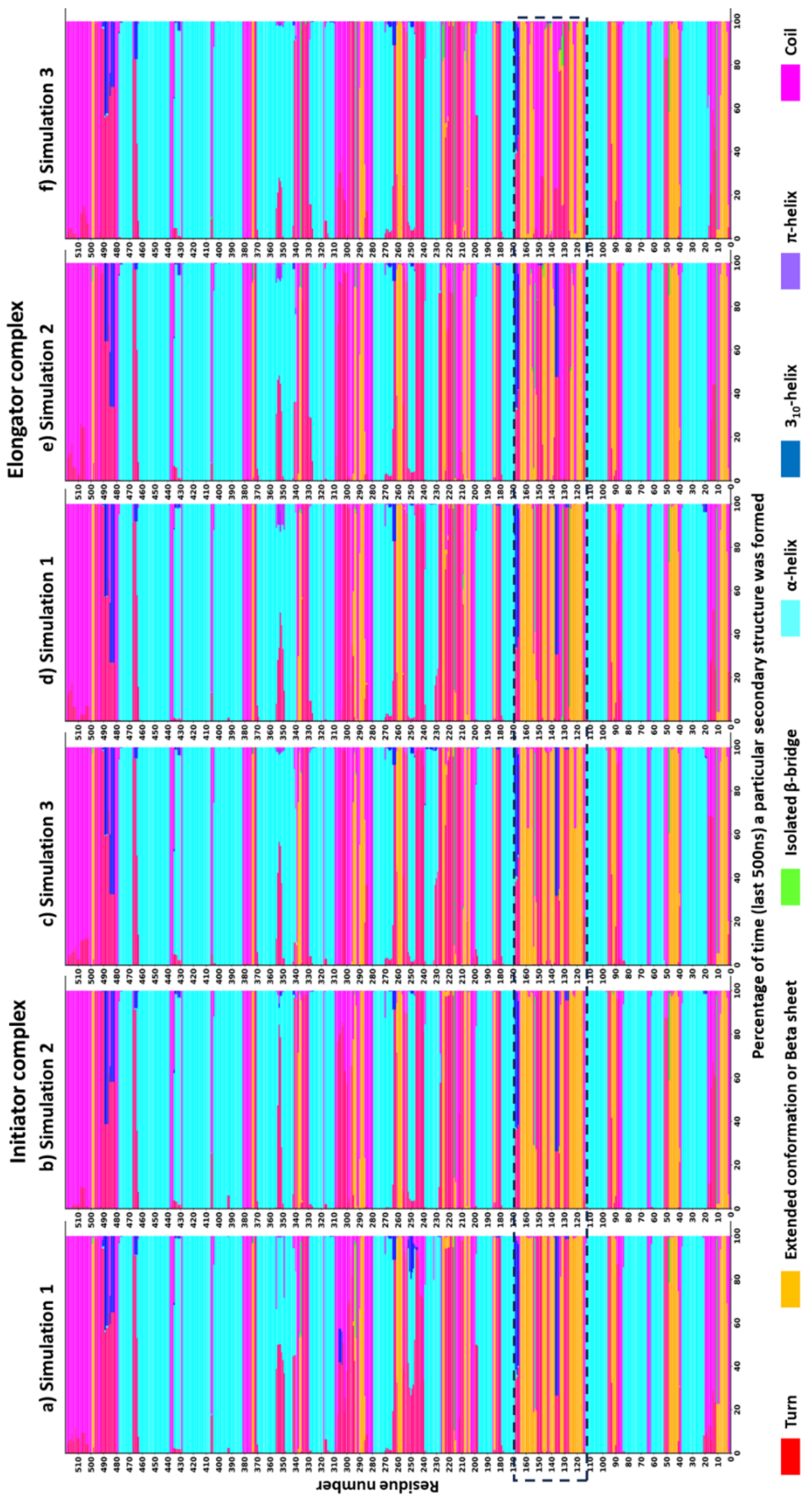

Figure S11. Residue-wise secondary structure assignment of MetRS. X-axis shows percentage of secondary structure formed by each residue of protein (Y-axis) across three simulations each for **(a-c)** initiator and **(d-f)** elongator complexes.

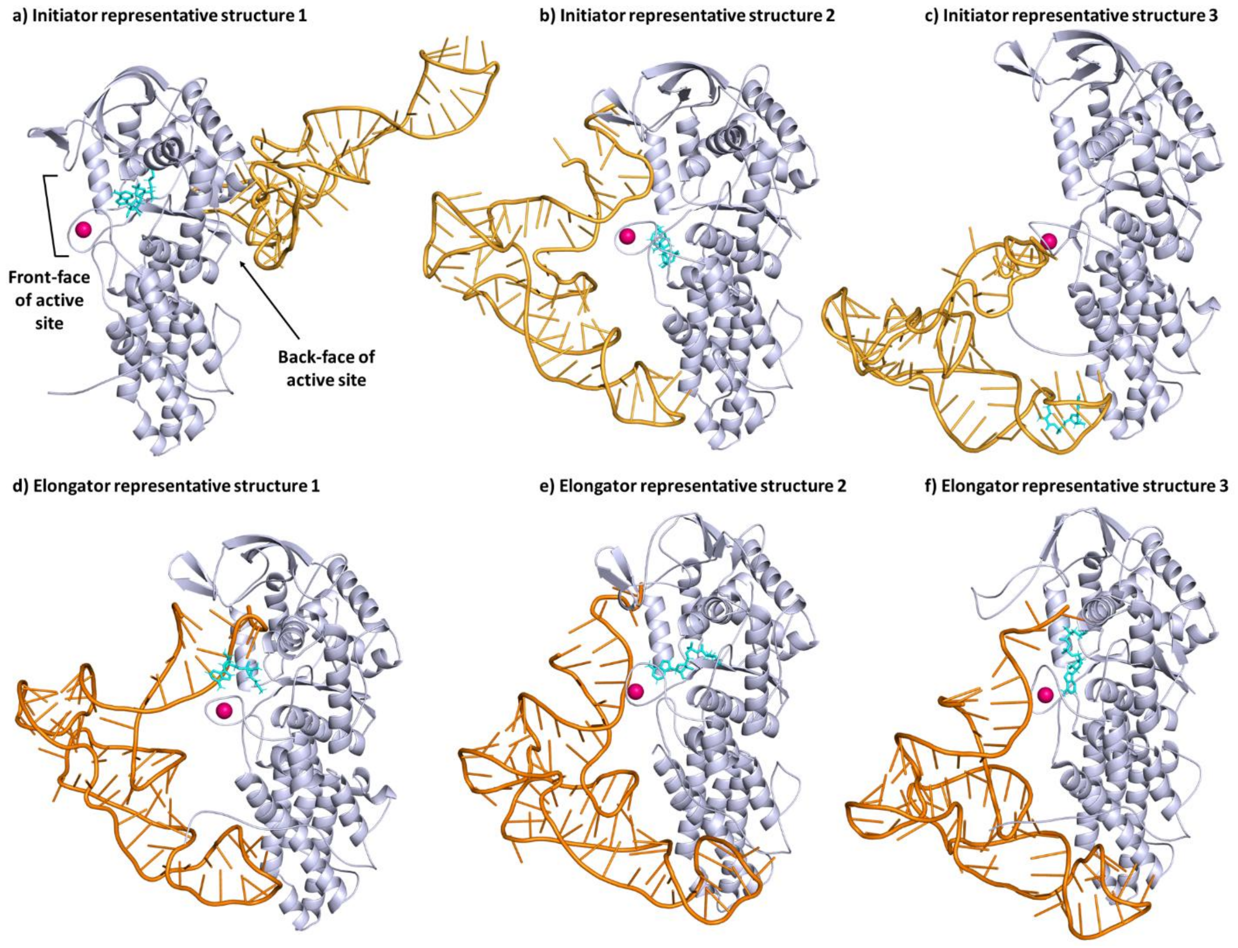

Figure S12. Representative structures from each simulation run of initiator and elongator tRNA. (a-c) initiator tRNA complex. **(d-f)** elongator tRNA complex.

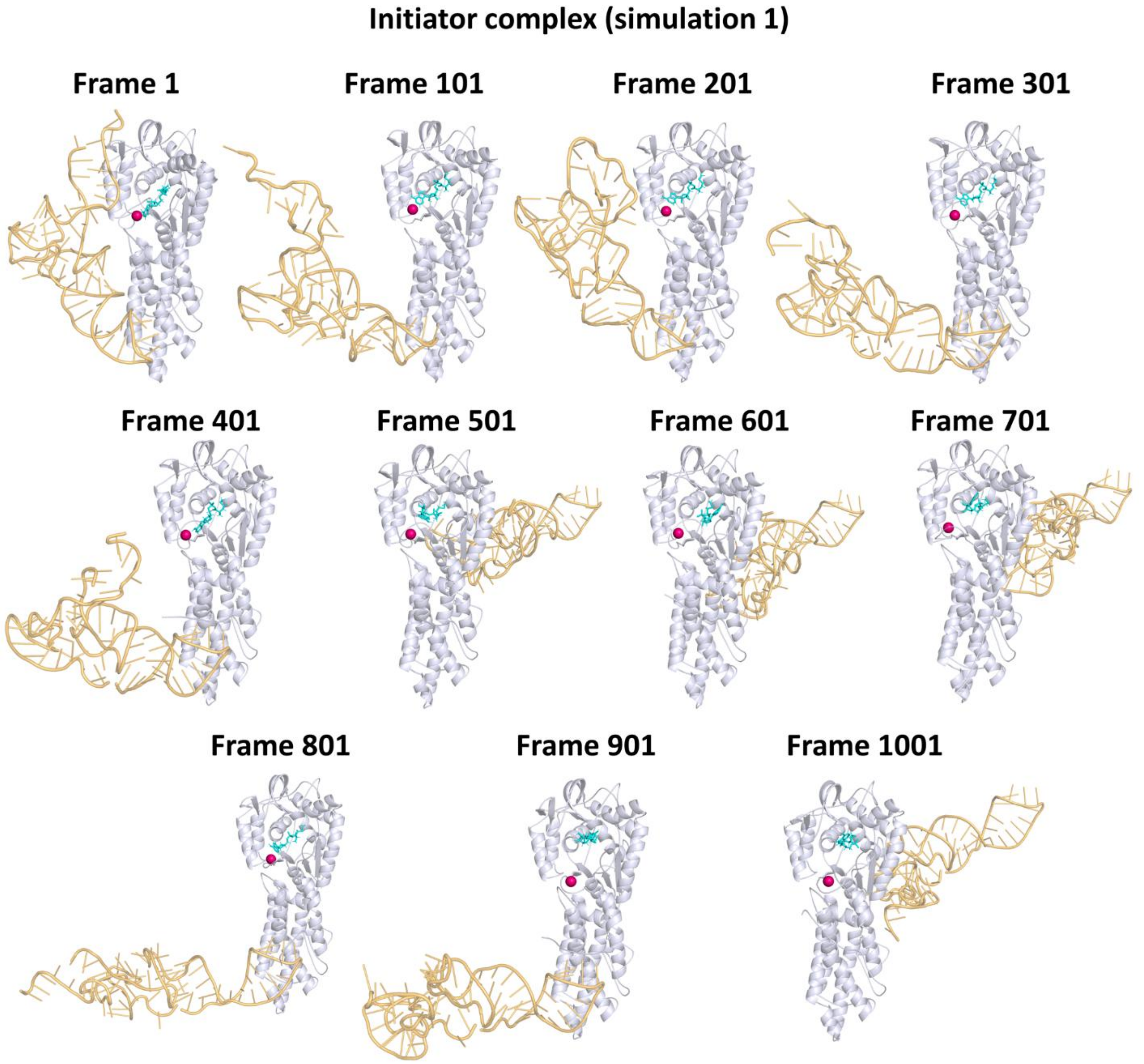

Figure S13. Trajectory snapshots from simulation 1 of the initiator complex. Frame 1 corresponds to 0 ns, frame 101 to 100 ns, and at the end frame 1001 to 1000 ns.

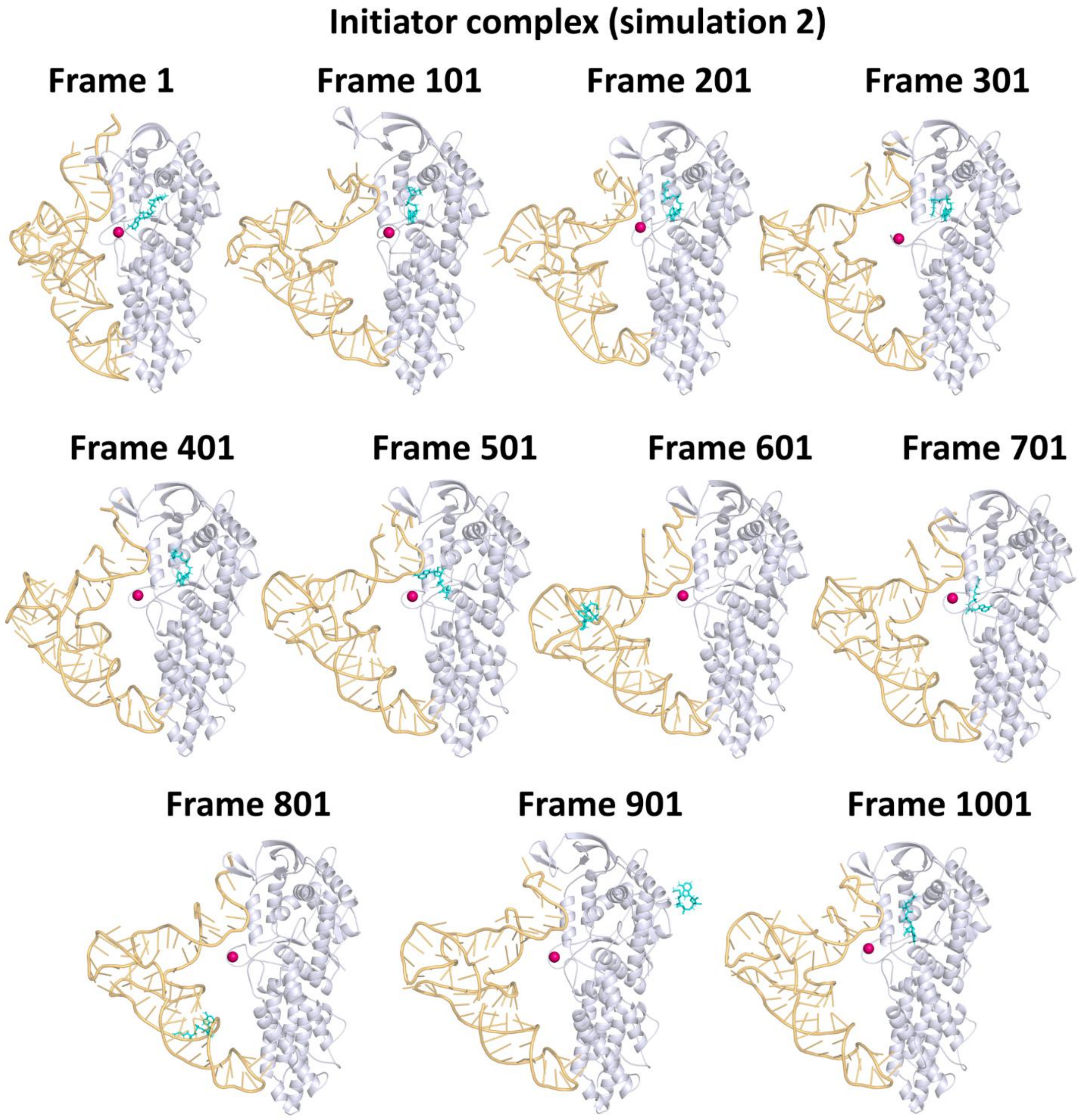

Figure S14. Trajectory snapshots from simulation 2 of the initiator complex. Frame 1 corresponds to 0 ns, frame 101 to 100 ns, and at the end frame 1001 to 1000 ns.

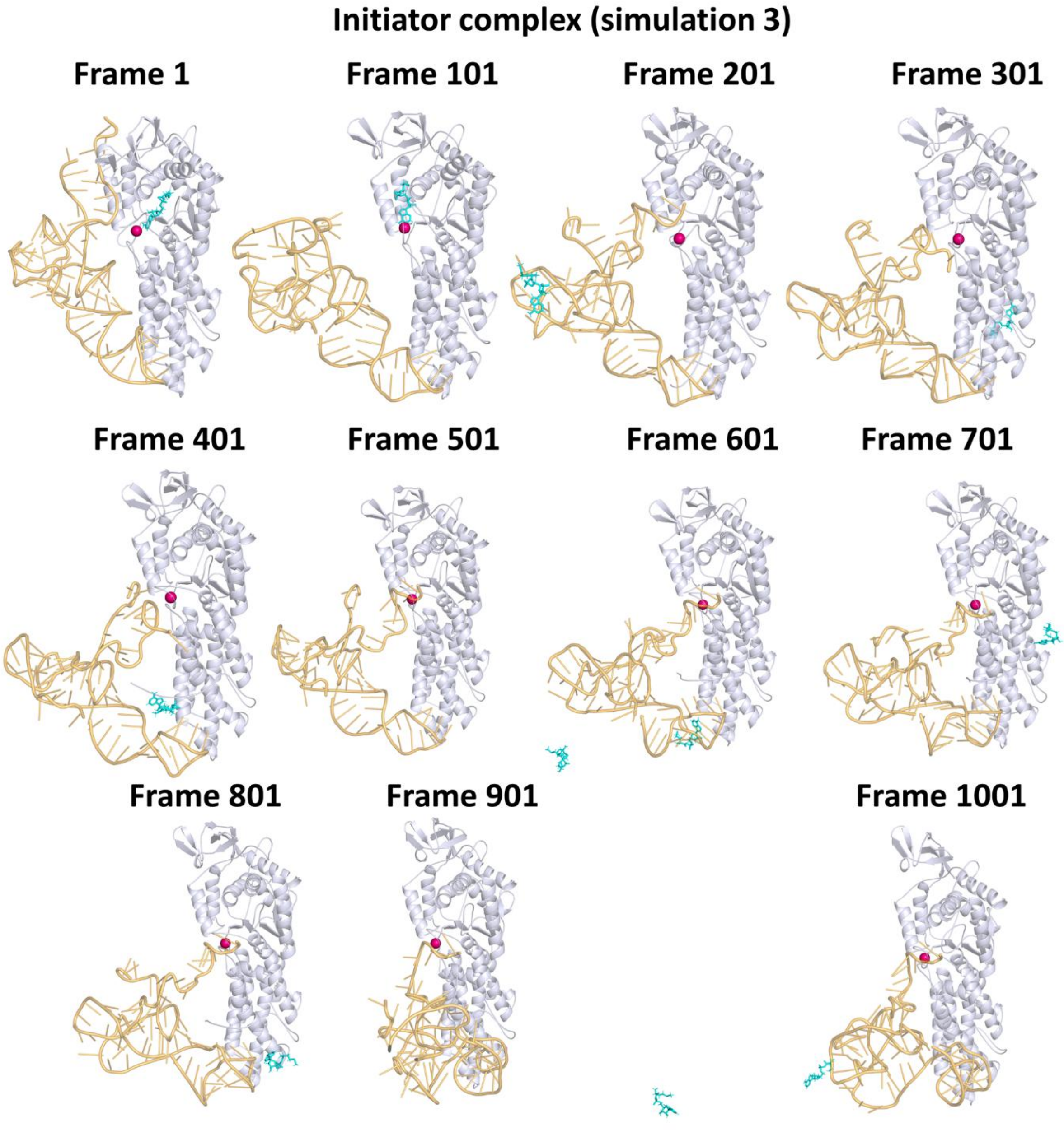

Figure S15. Trajectory snapshots from simulation 3 of the initiator complex. Frame 1 corresponds to 0 ns, frame 101 to 100 ns, and at the end frame 1001 to 1000 ns.

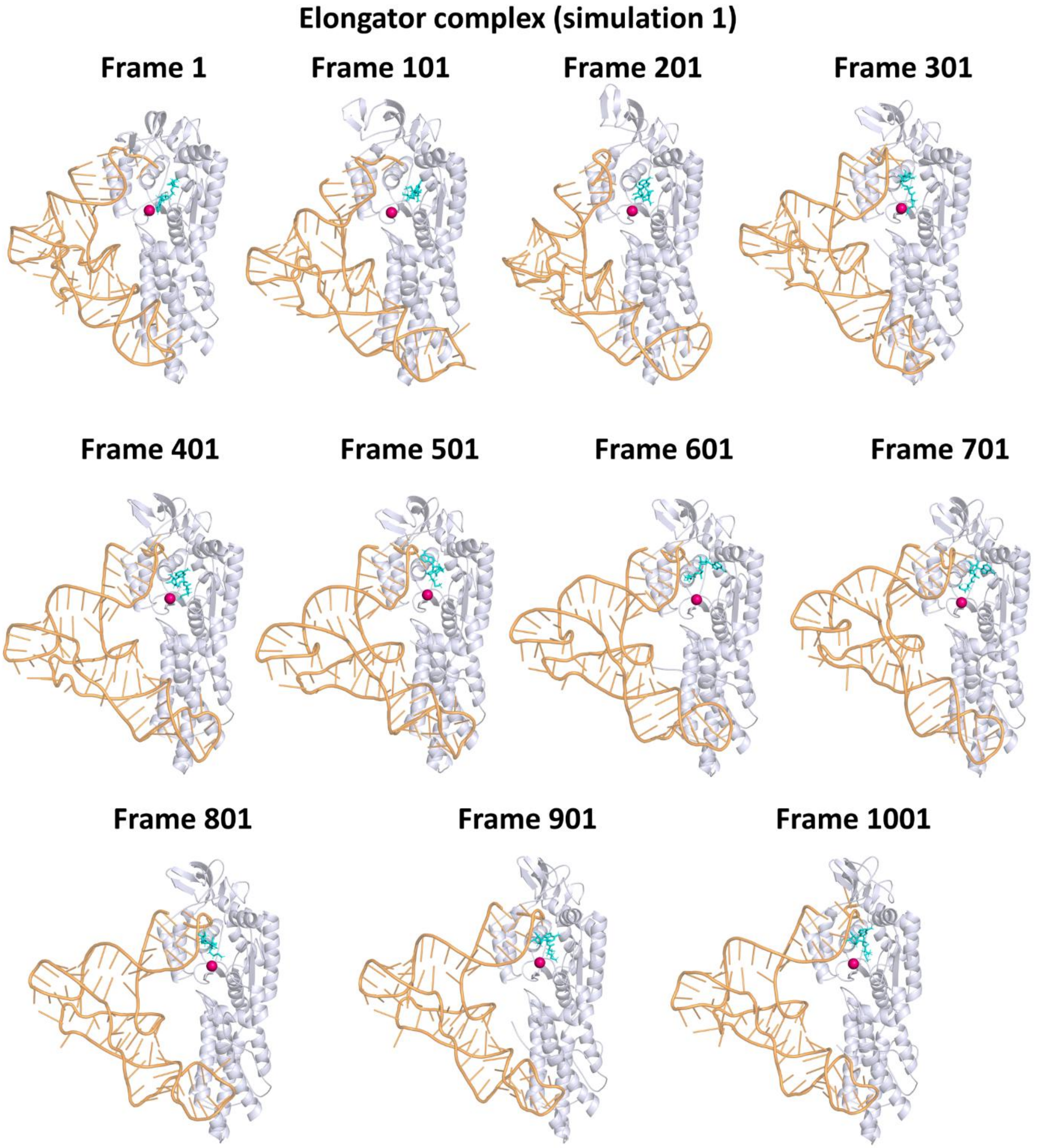

Figure S16. Trajectory snapshots from simulation 1 of the elongator complex. Frame 1 corresponds to 0 ns, frame 101 to 100 ns, and at the end frame 1001 to 1000 ns.

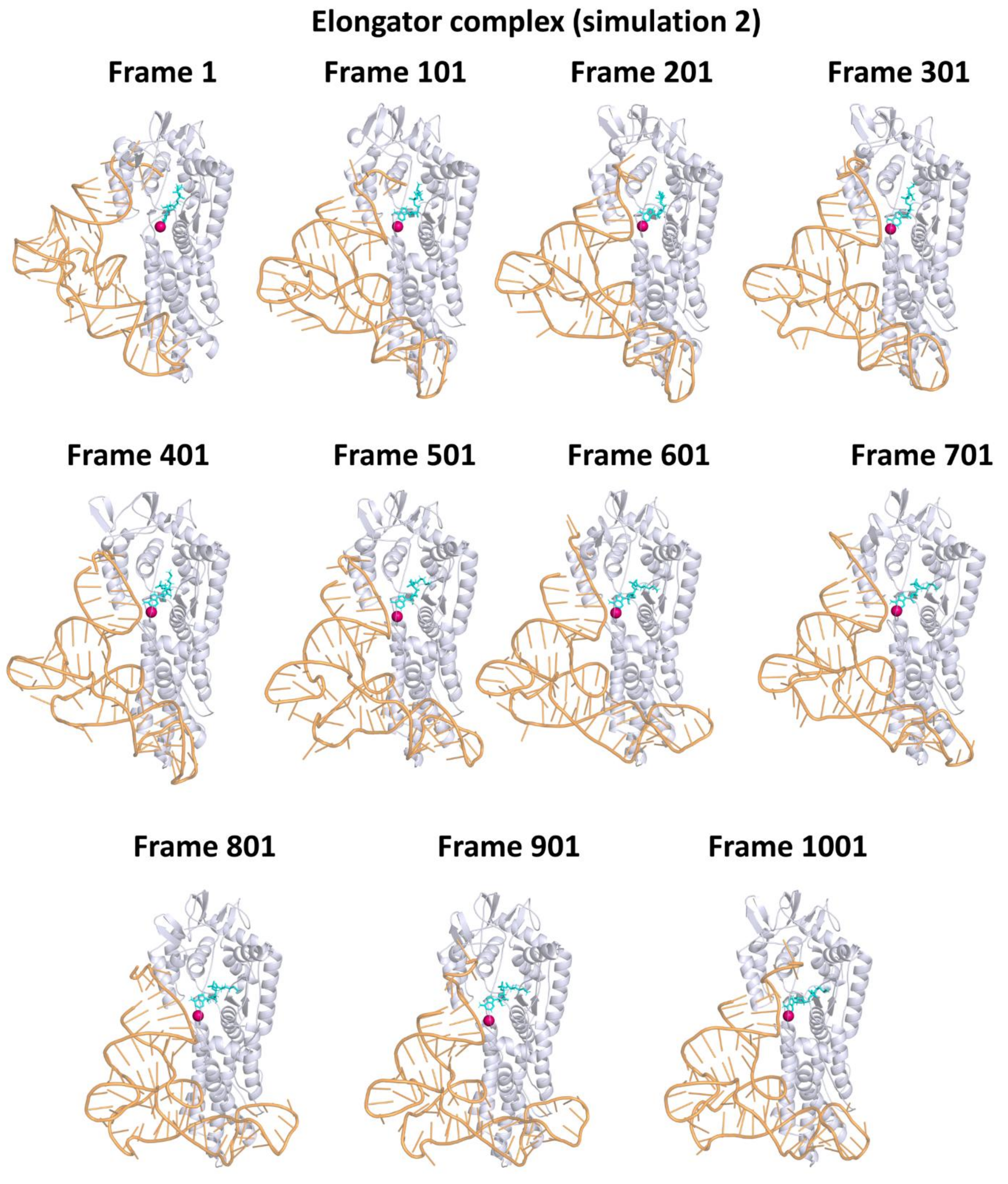

Figure S17. Trajectory snapshots from simulation 2 of the elongator complex. Frame 1 corresponds to 0 ns, frame 101 to 100 ns, and at the end frame 1001 to 1000 ns.

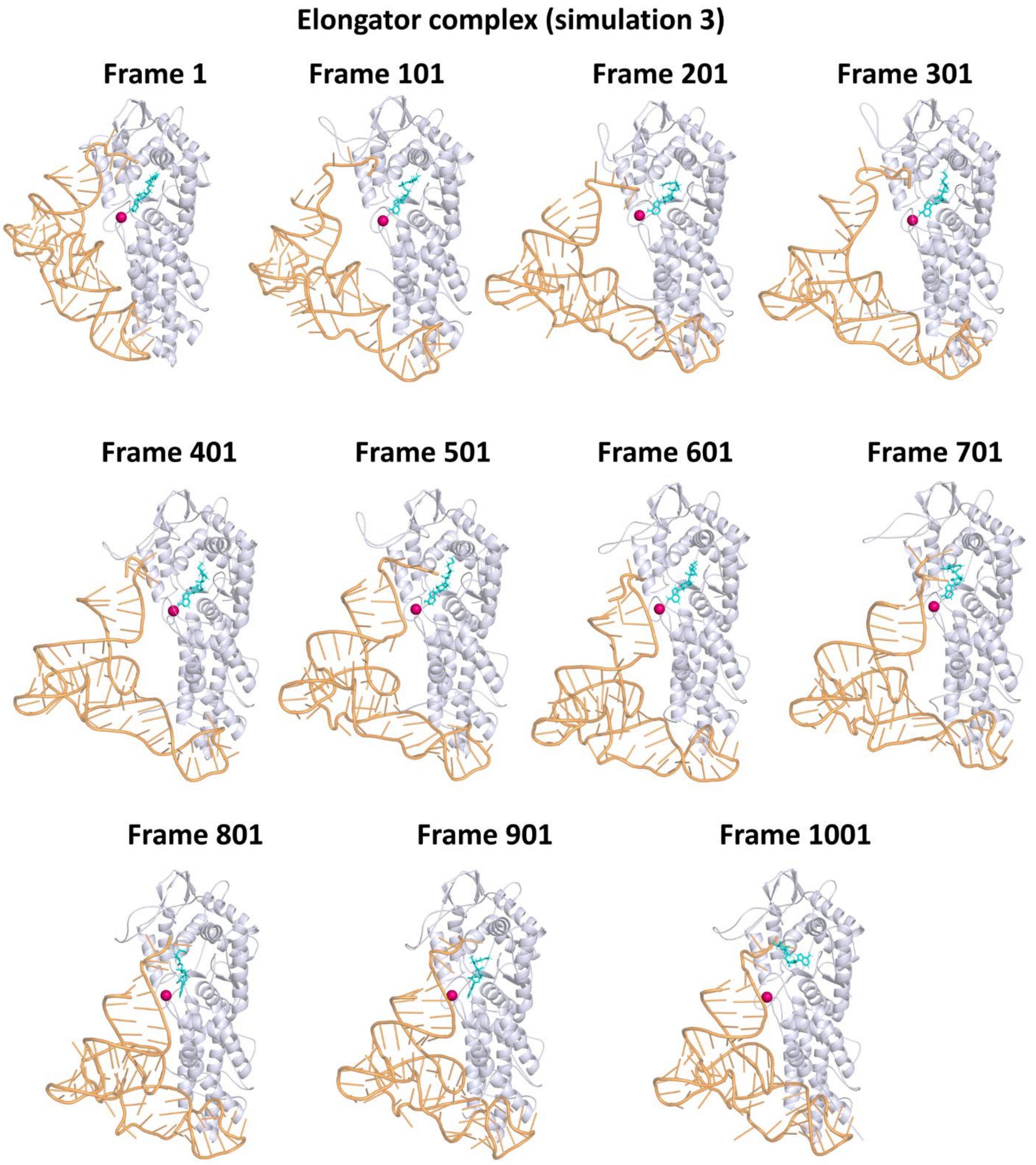

Figure S18. Trajectory snapshots from simulation 3 of the elongator complex. Frame 1 corresponds to 0 ns, frame 101 to 100 ns, and at the end frame 1001 to 1000 ns.

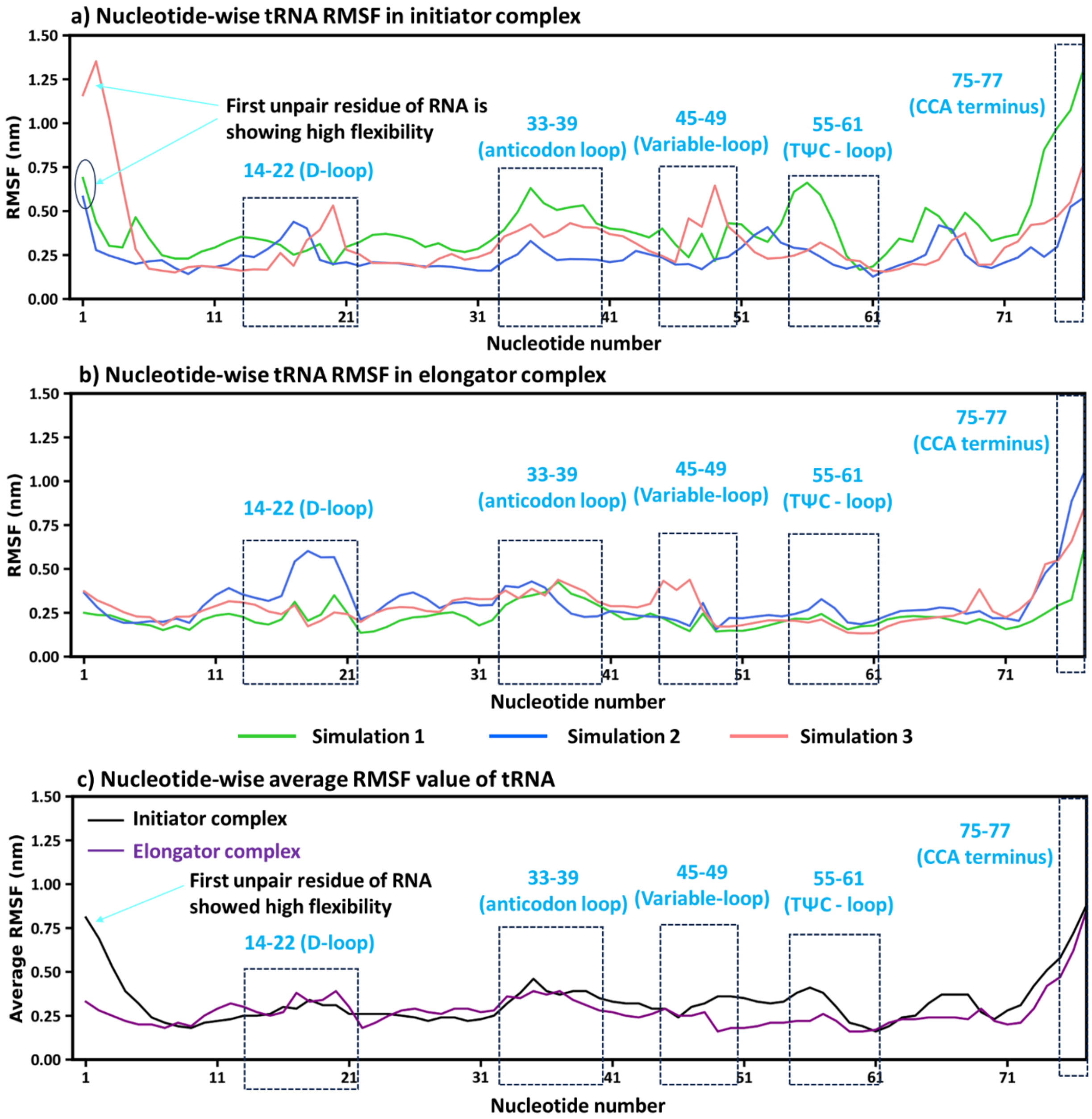

Figure S19. Nucleotide-wise RMSF analysis of tRNA in (a) initiator complex and **(b)** elongator complex. **(c)** Comparison of average RMSF values to show flexibility of nucleotides at the 5' end, D-loop (14-22), anticodon loop (33-39), variable loop (45-49), TΨC loop (55-61) and CCA terminus.

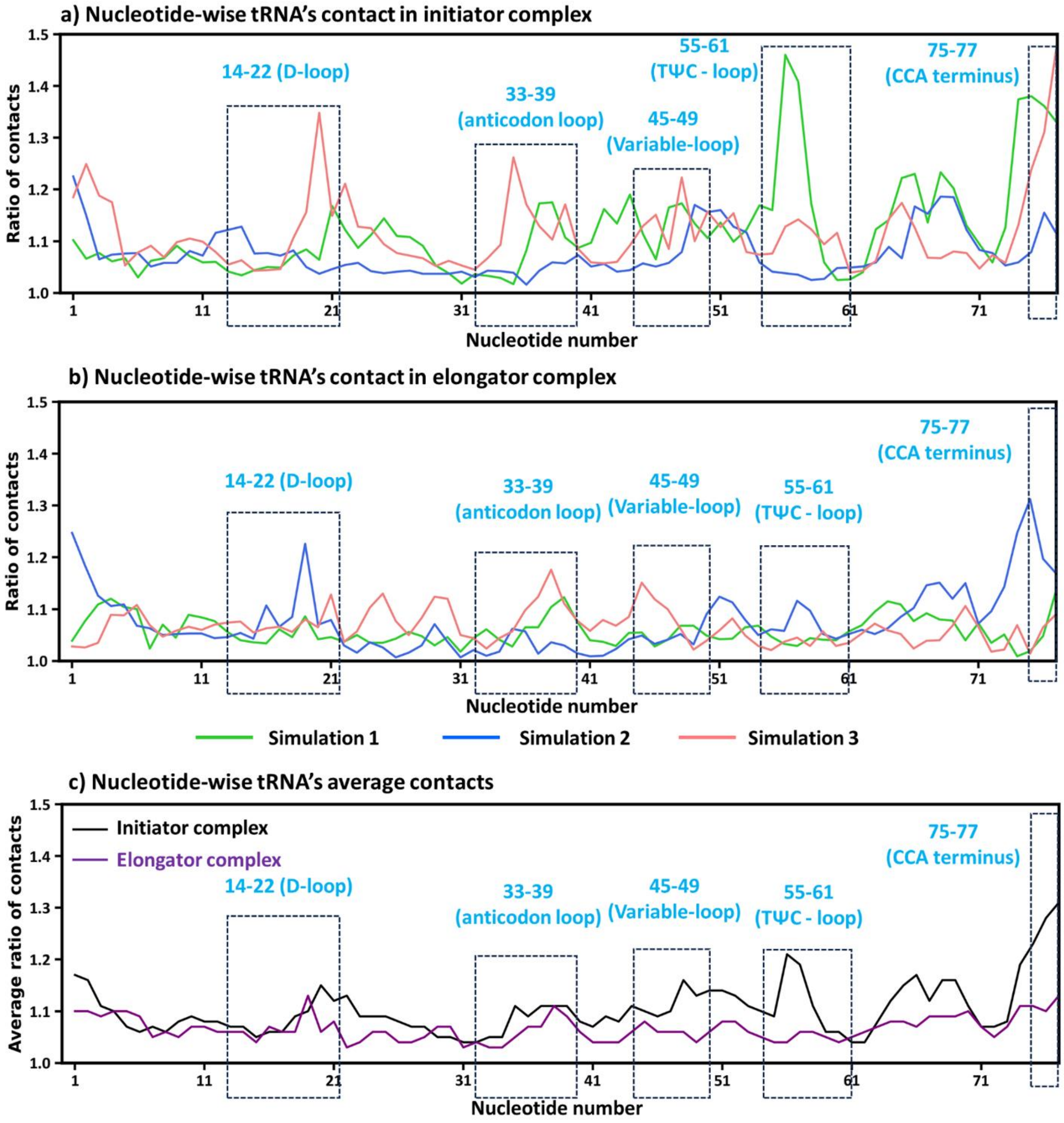

Figure S20. Nucleotide-wise contact analysis of tRNA in (a) initiator complex and **(b)** elongator complex. **(c)** Average contact ratio comparison reveals higher interaction dynamics in the initiator complex, especially at functionally important tRNA motifs. Here "contacts" means count of the number of different atomic contacts formed by each tRNA nucleotide (x-axis) with atoms of other nucleotides during simulations. "Ratio of contacts" is the total number of contacts divided by their mean value.

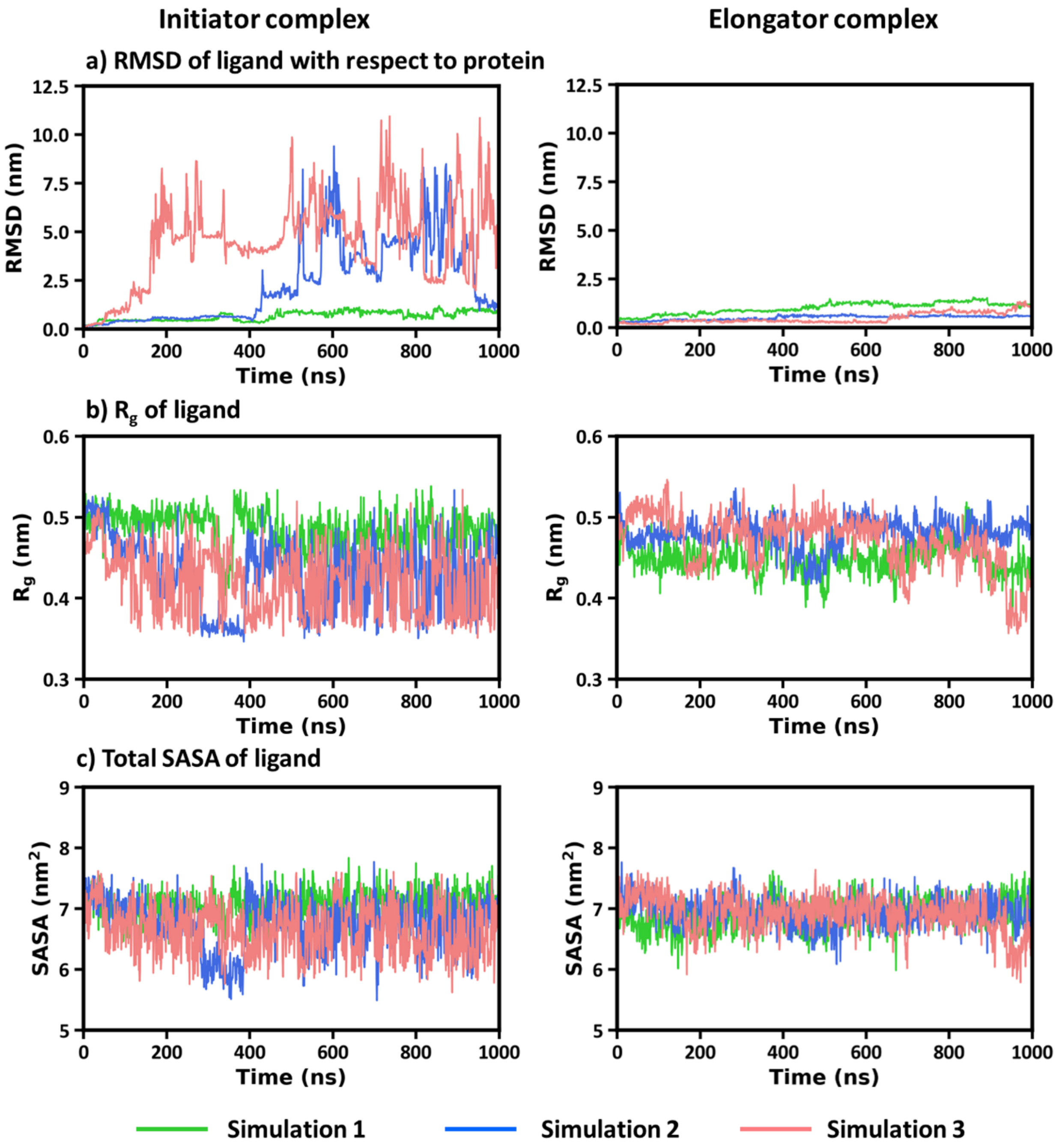

Figure S21. Structural properties of the ligand (Met-AMP) in the initiator and elongator complexes. (a) RMSD, **(b)** R_g, and **(c)** SASA of ligand.

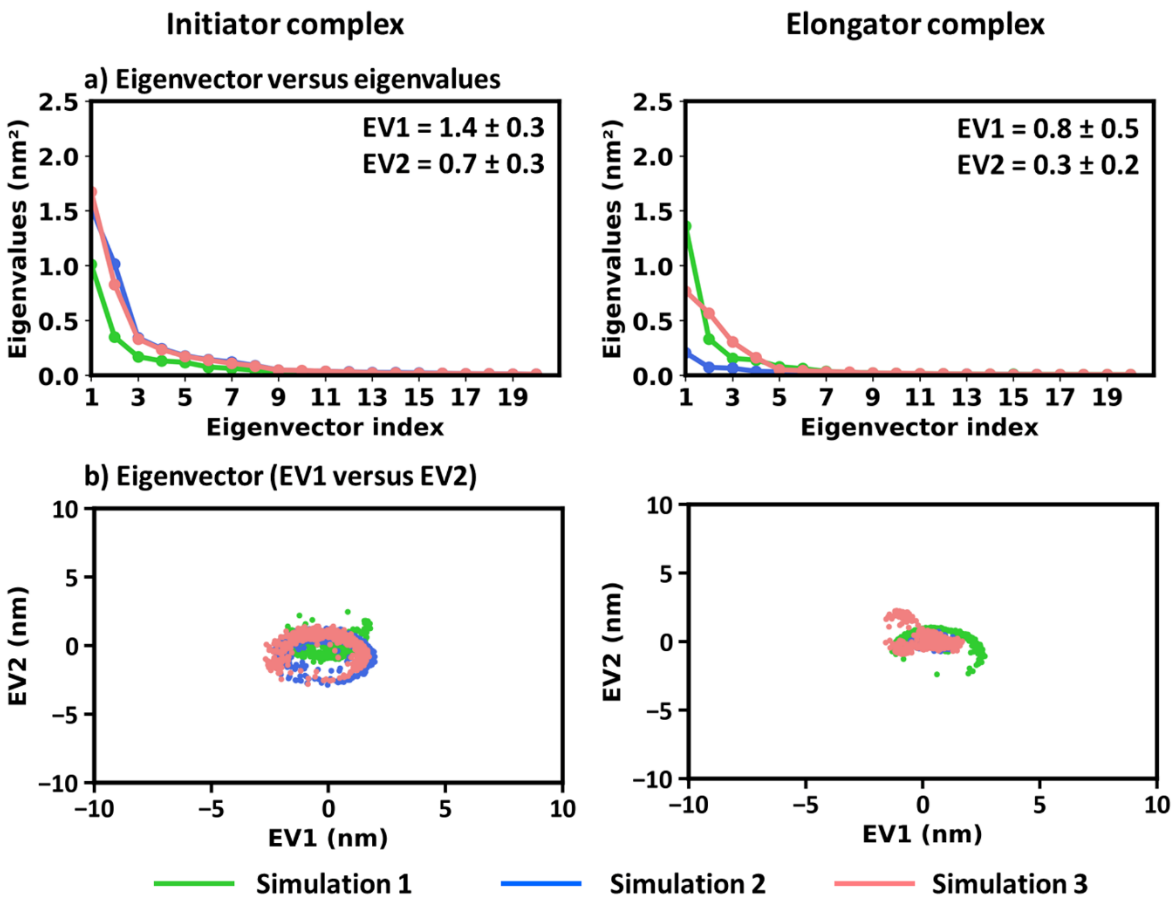

Figure S22. PCA of the ligand (Met-AMP) in initiator and elongator complexes. (a) Eigenvector versus eigenvalue plots. Average and standard deviation of eigenvalues for EV1 and EV2 over three simulations are provided. **(b)** 2D projection of eigenvectors (EV1 versus EV2).

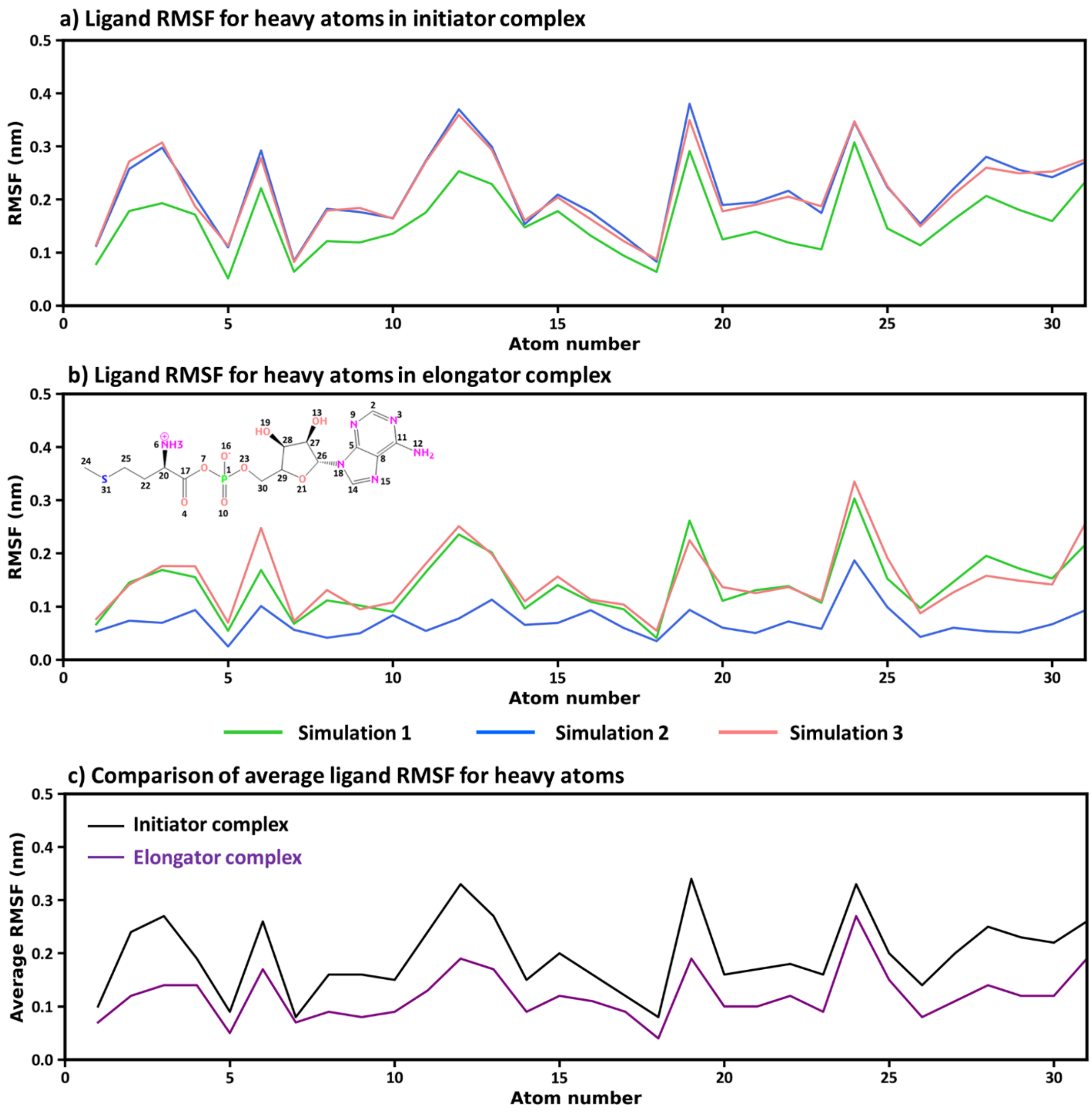

Figure S23. RMSF analysis of heavy atoms in the ligand molecule. (a) and **(b)** depict the heavy-atom RMSF of ligand in the initiator and elongator complexes, respectively. **(c)** Comparison of average RMSF values confirms reduced ligand mobility in the elongator complex.

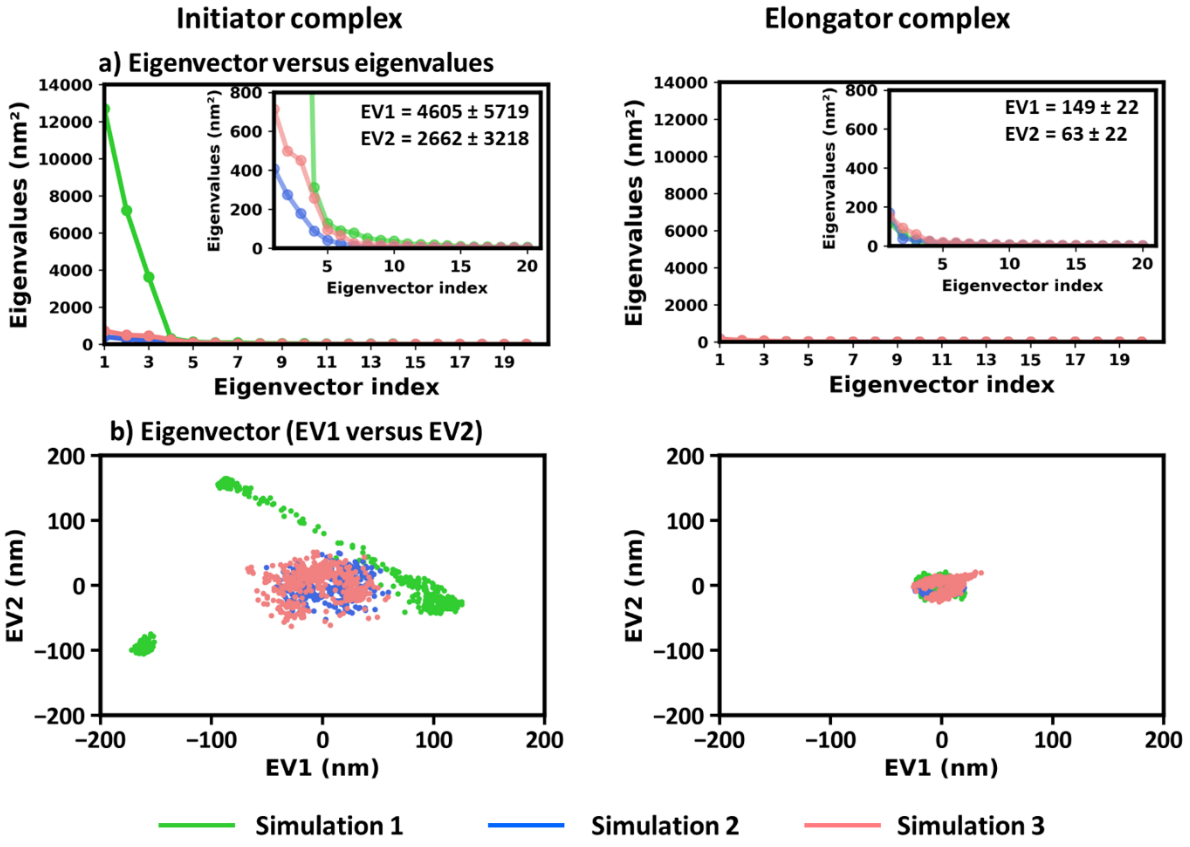

Figure S24. PCA of complex (containing protein, ligand and tRNA) in initiator and elongator complexes. **(a)** Eigenvector versus eigenvalue plots. Average and standard deviation of eigenvalues for EV1 and EV2 over three simulations are provided. **(b)** 2D projection of eigenvectors (EV1 versus EV2).

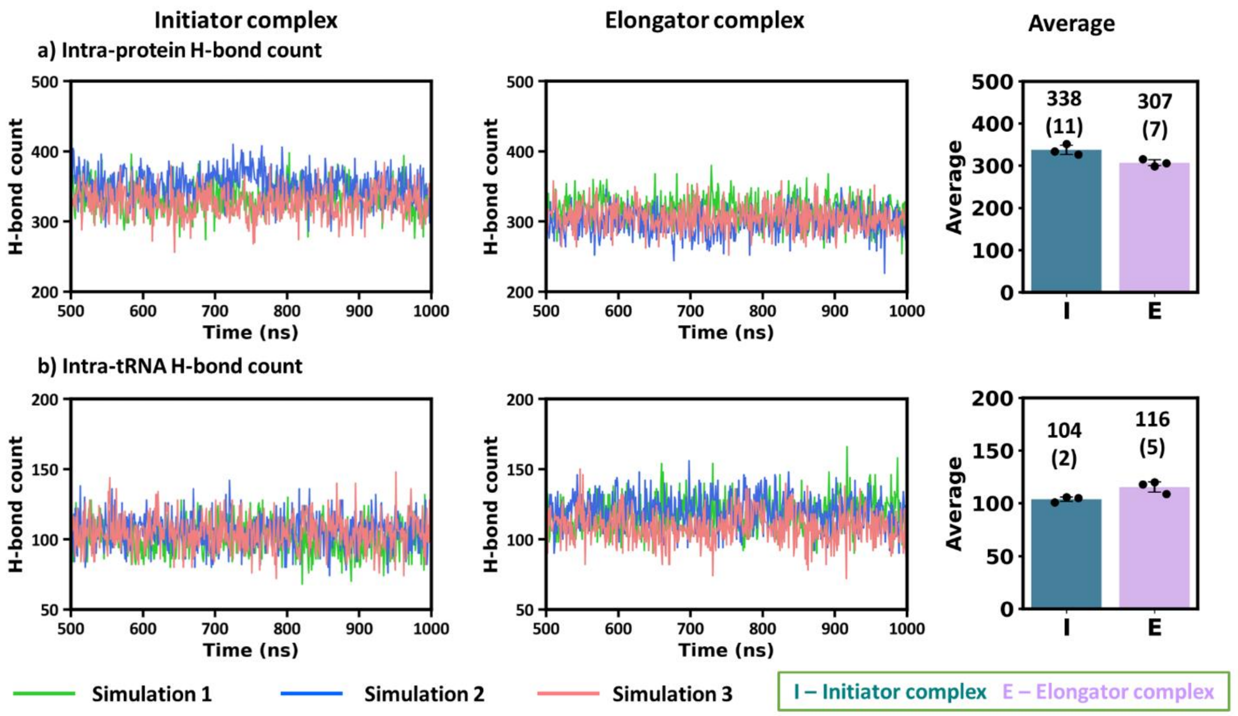

Figure S25. Intramolecular hydrogen bonding of protein and tRNA. Figure shows the H-bond count over time within **(a)** protein and **(b)** tRNA respectively, in initiator and elongator complexes. Average and standard deviation (value in bracket; error bar) plot reveal higher intra-protein H-bonds in the initiator, while intra-tRNA H-bonding is slightly greater in the elongator complex.

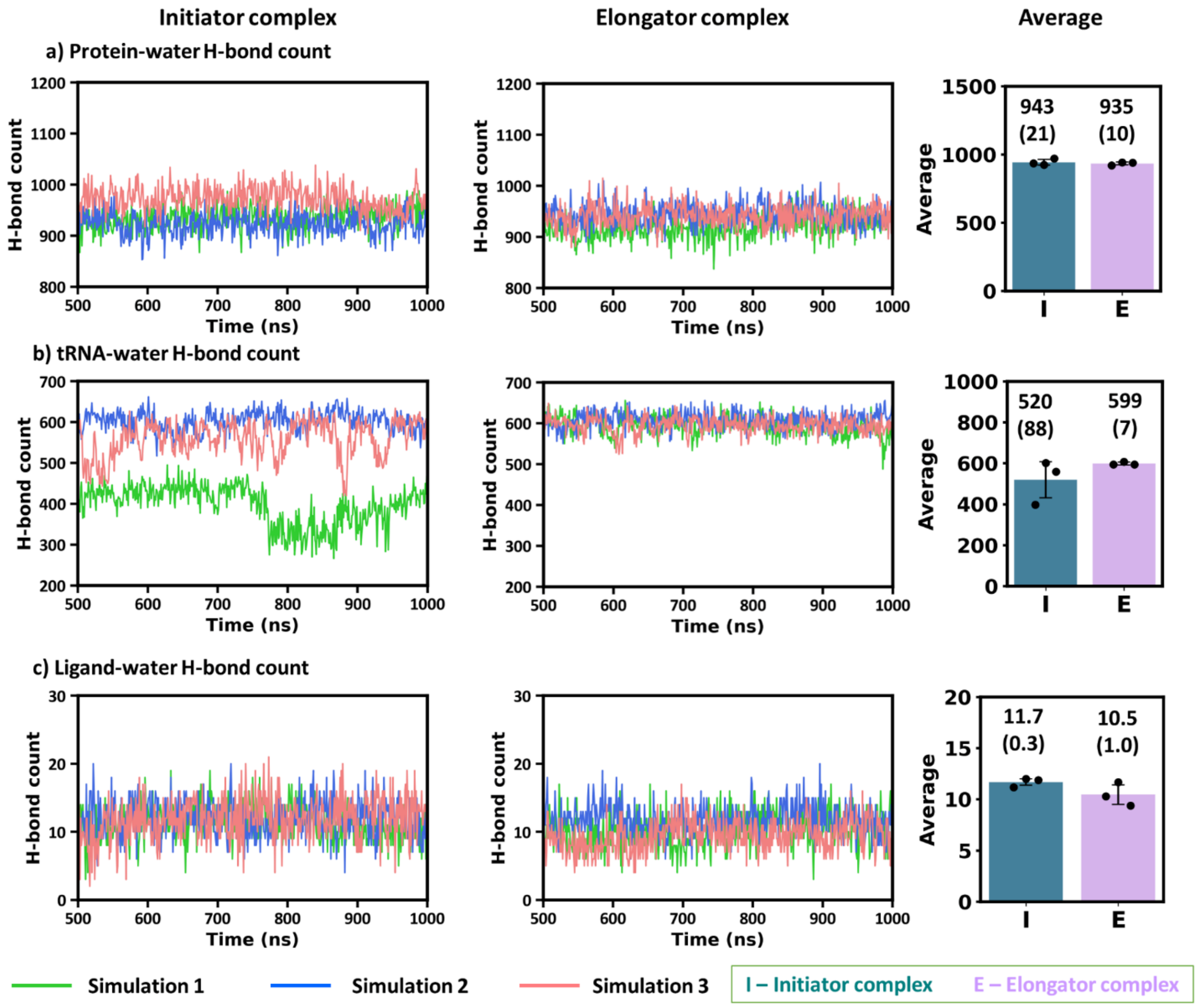

Figure S26. Hydrogen bonds with water in initiator (I) and elongator (E) complexes. H-bond counts for **(a)** protein-water, **(b)** tRNA-water, and **(c)** ligand-water interactions. Bar plots summarizing the average and standard deviation (value in bracket; error bar) of H-bond counts.

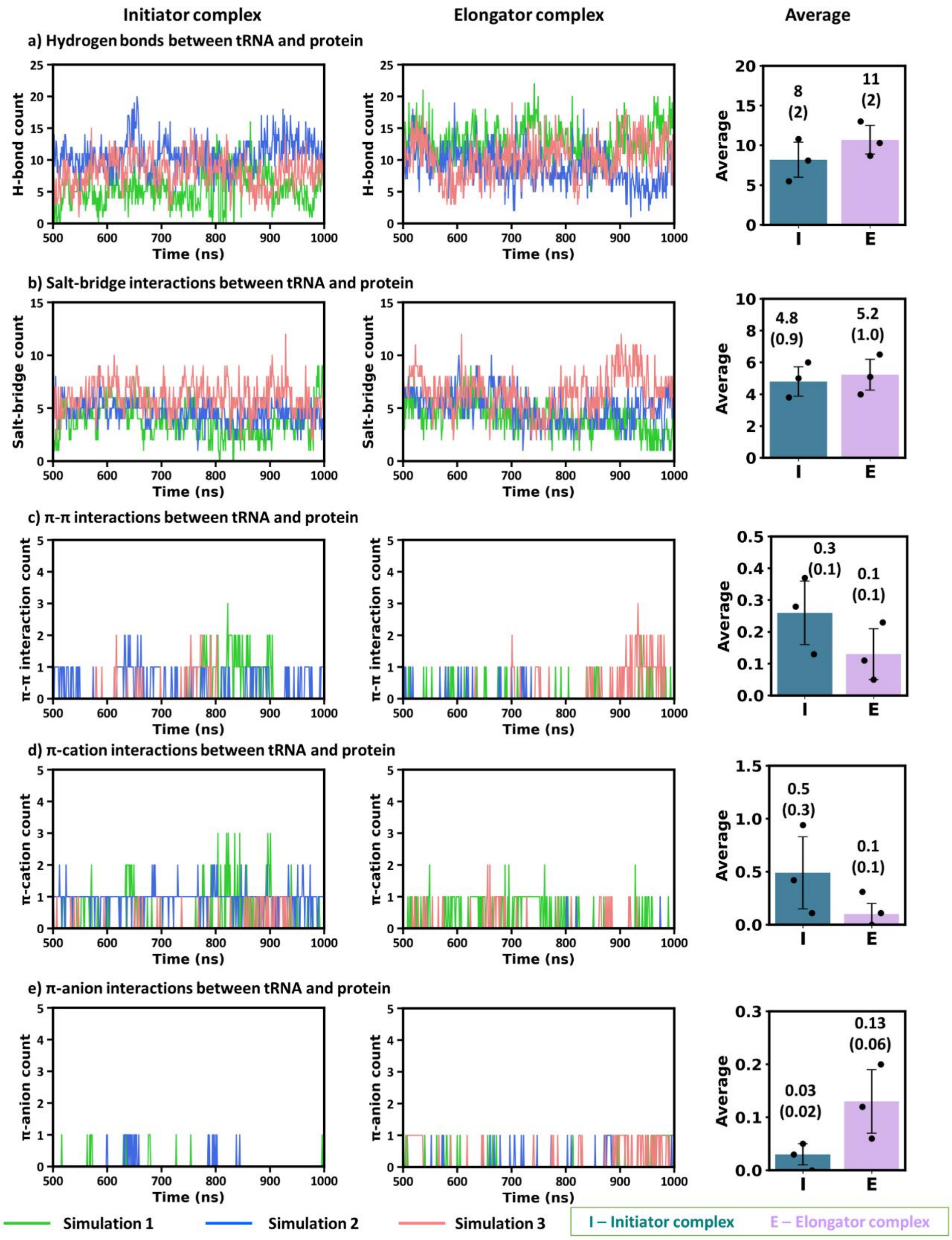

Figure S27. Intermolecular interactions between tRNA and protein in initiator and elongator complexes. (a) Hydrogen bonds. **(b)** Salt-bridge interactions. **(c-e)** Occurrence of π-π, π-cation, and π-anion interactions over time. Bar plots summarizing the average and standard deviation (value in bracket; error bar) of interaction count.

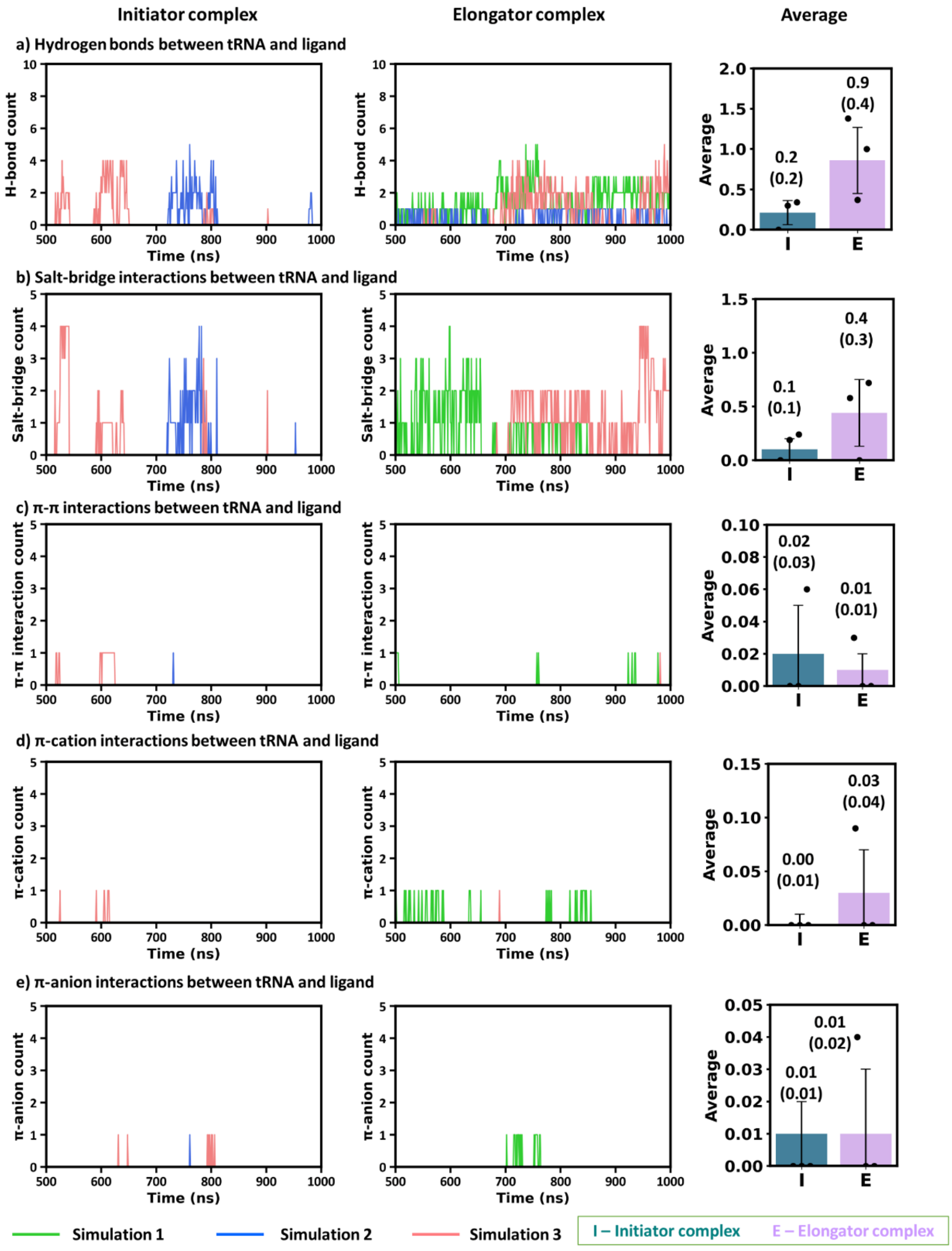

Figure S28. Intermolecular interactions between tRNA and ligand in initiator (I) and elongator (E) complexes. **(a)** Hydrogen bonds. **(b)** Salt-bridge interactions. **(c-e)** Occurrence of π-π, π-cation, and π-anion interactions over time. Bar plots summarizing the average and standard deviation (value in bracket; error bar) of interaction count.

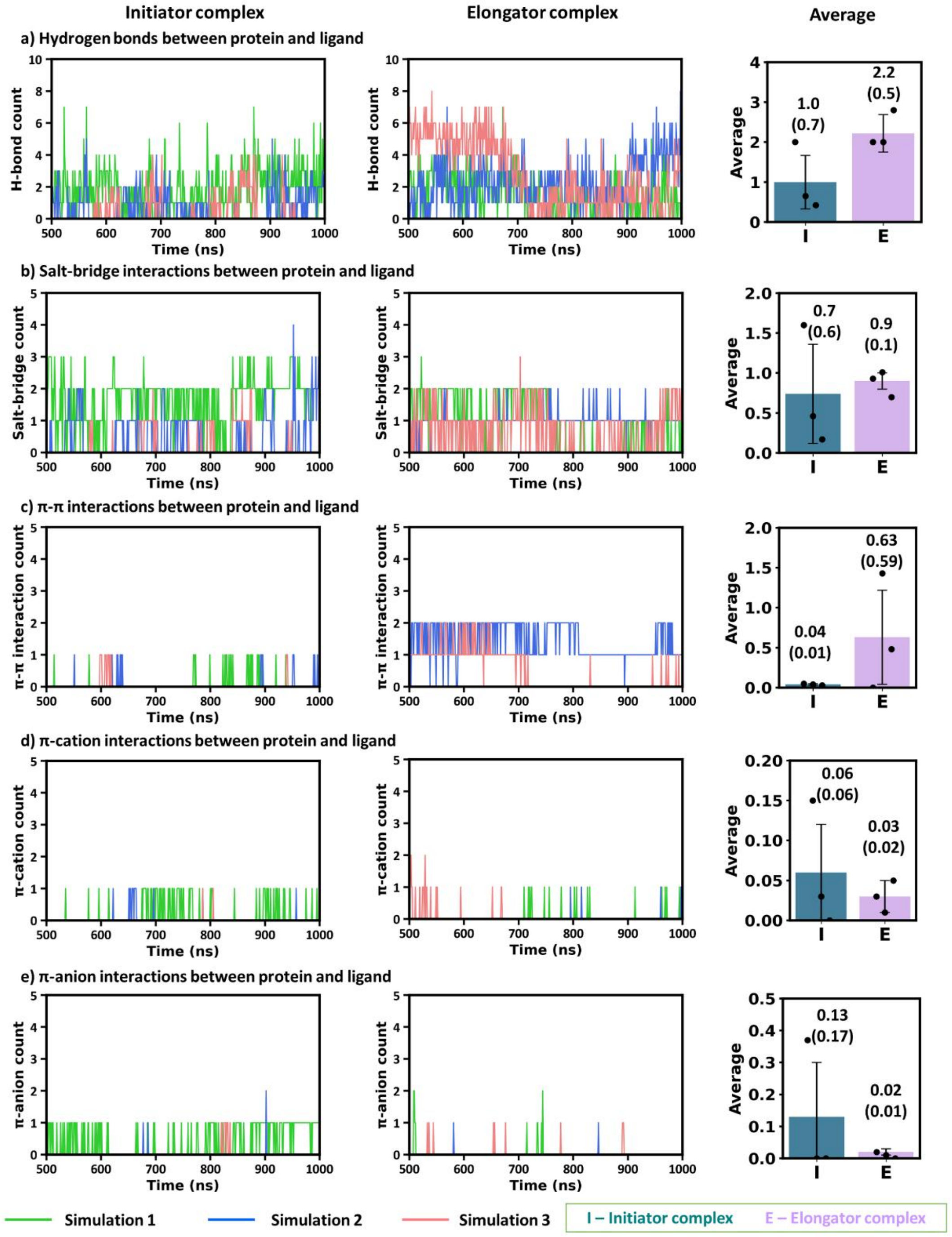

Figure S29. Intermolecular interactions between protein and ligand in initiator (I) and elongator (E) complexes. (a) Hydrogen bonds. **(b)** Salt-bridge interactions. **(c-e)** Occurrence of π-π, π-cation, and π-anion interactions over time. Bar plots summarizing the average and standard deviation (value in bracket; error bar) of interaction count.

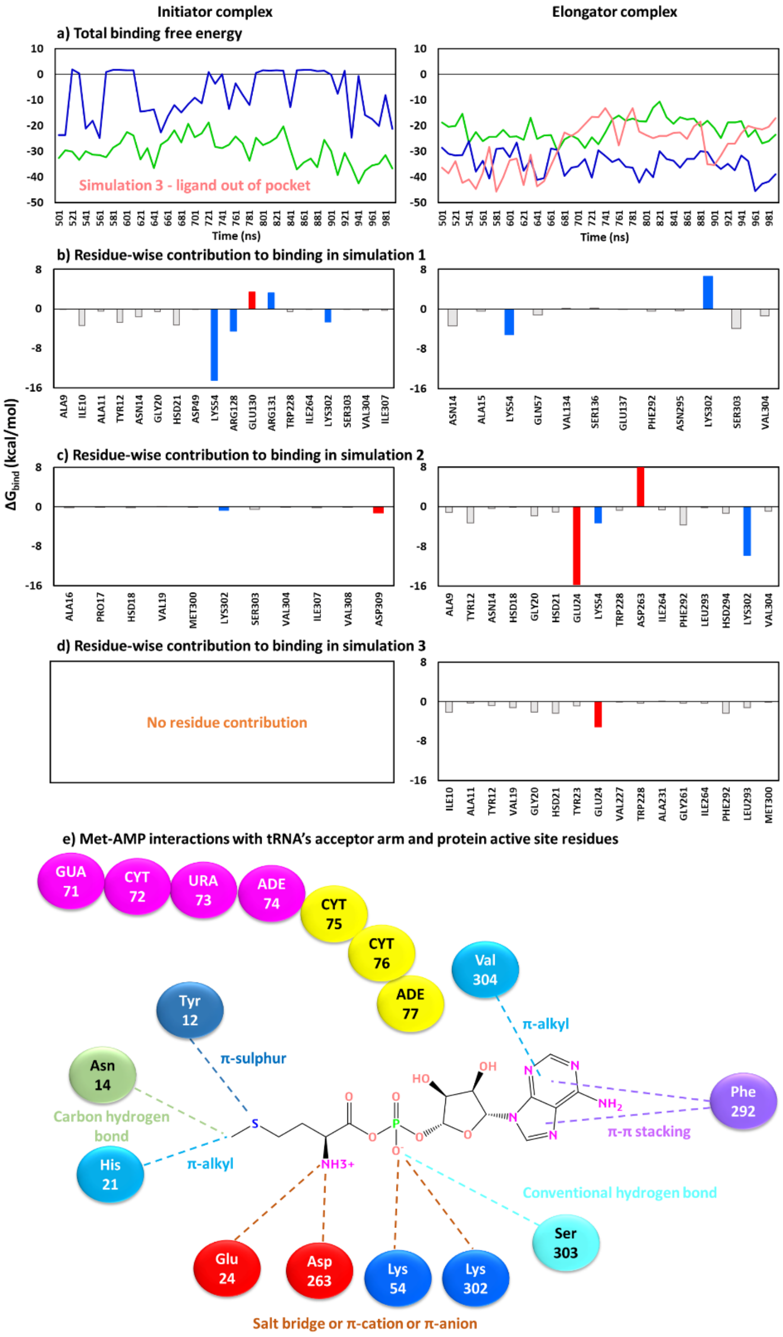

Figure S30. Binding energy for protein-ligand binding in initiator and elongator complexes. (a) Total binding free energy (ΔG_{bind}) over time for three simulations. **(b-d)** Residue-wise contribution to binding energy over three simulations, highlighting residues involved in ligand binding (positively charged residue - blue, negatively charged - red and neutral - light gray). **(e)** Important interactions of ligand (Met-AMP) observed during simulations of both complexes.

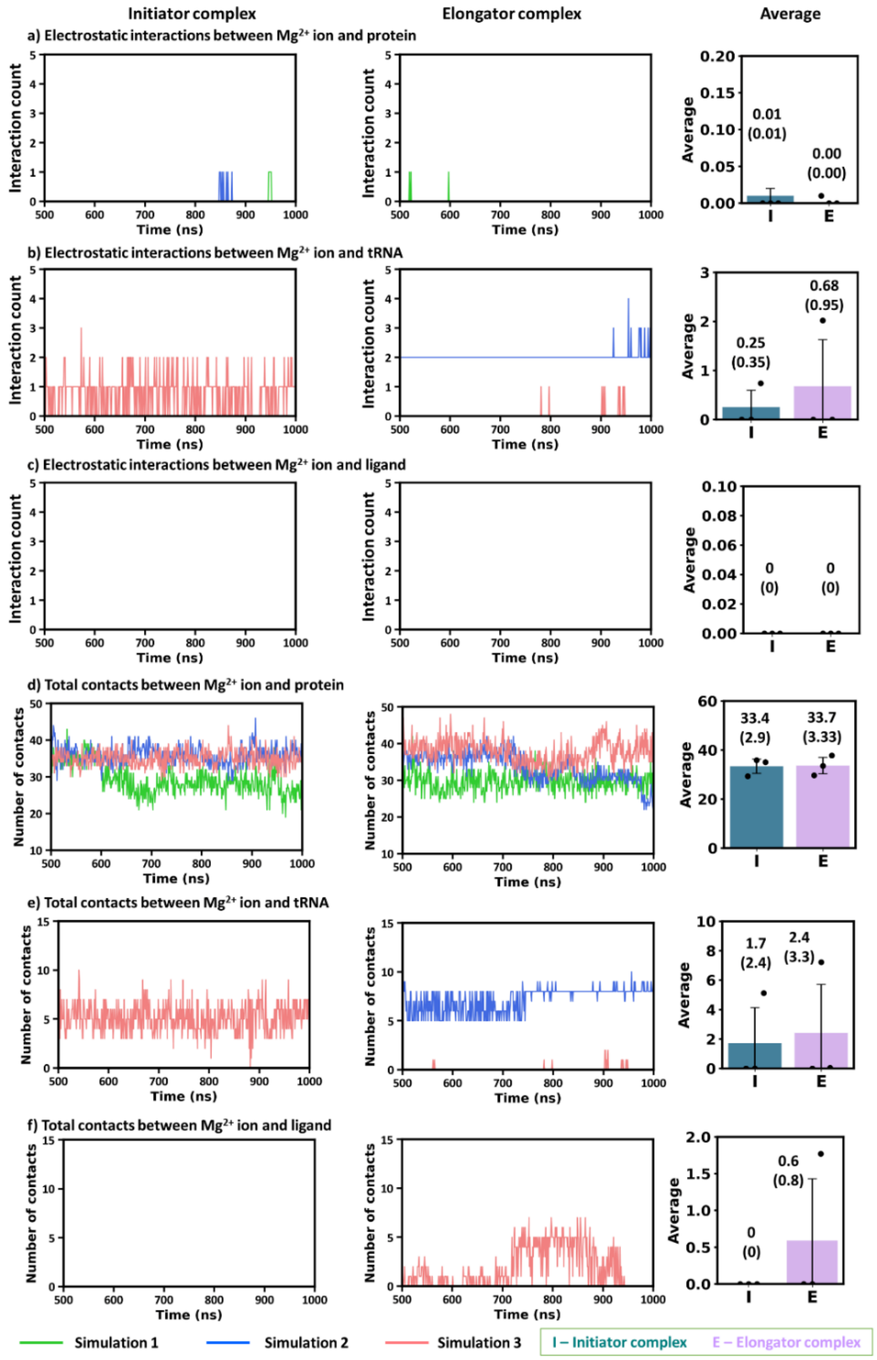

Figure S31. Interactions of the Mg^{2+} ion in the initiator (I) and elongator (E) complexes. Ionic interactions formed by Mg^{2+} with the **(a)** protein, **(b)** tRNA, and **(c)** ligand. Total atomic contacts formed within 5 Å of Mg^{2+} with the **(d)** protein, **(e)** tRNA, and **(f)** ligand. The data are represented as line plots showing variations along the trajectories and as bar plots showing average interaction counts with standard deviations (values in bracket; error bar). Approximately 33 atomic contacts were present between Mg^{2+} and protein, indicating that Mg^{2+} was present at the MetRS active site.

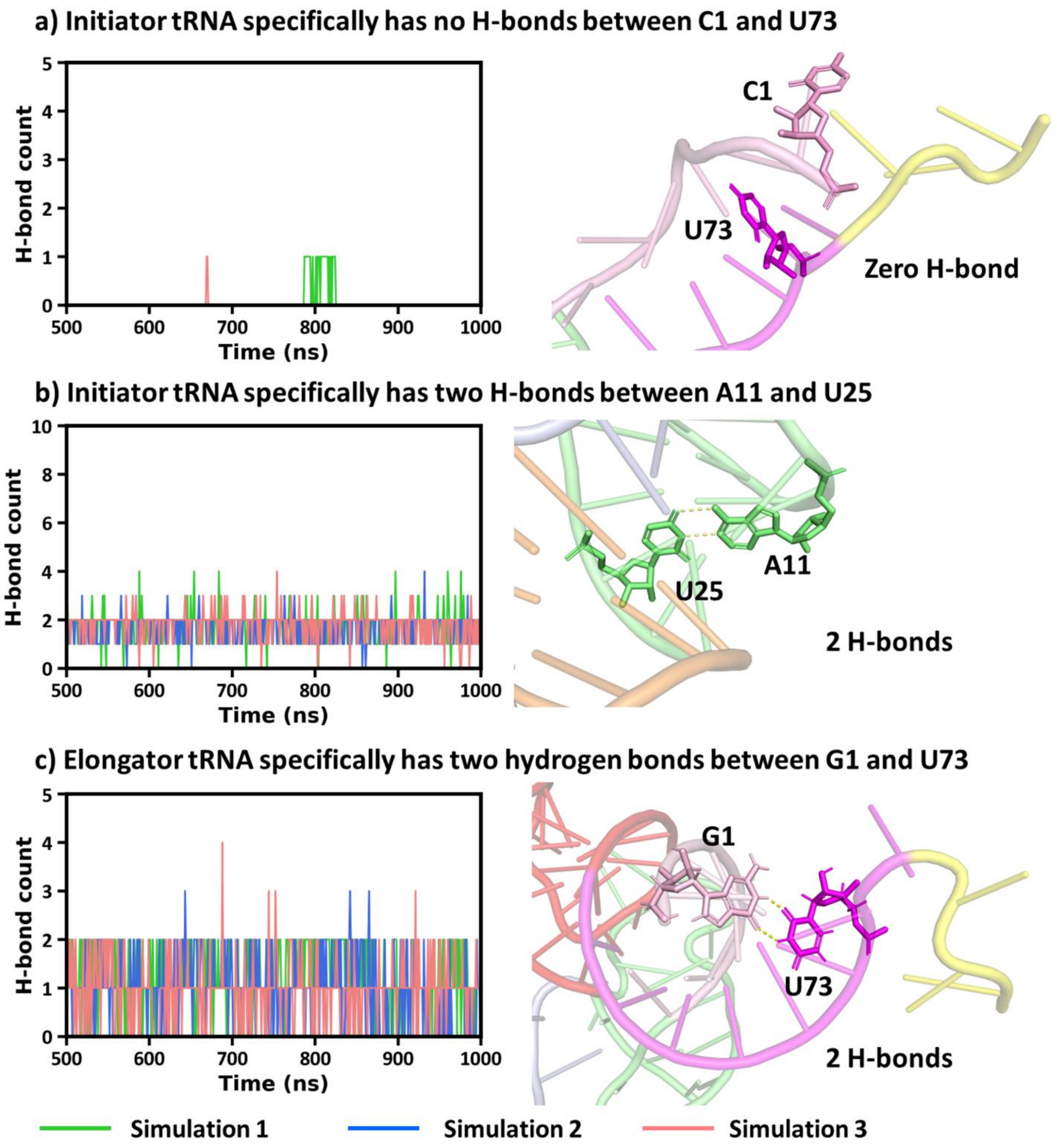

Figure S32. Base-specific hydrogen bonding patterns in initiator and elongator tRNAs. (a) Initiator tRNA lacks hydrogen bonds between C1 and U73, indicating an open end. **(b)** A stable A11-U25 base pair is observed in initiator tRNA with consistent hydrogen bonding. **(c)** Elongator tRNA shows a G1-U73 pair stabilized by two hydrogen bonds, reflecting canonical base pairing at the acceptor stem.

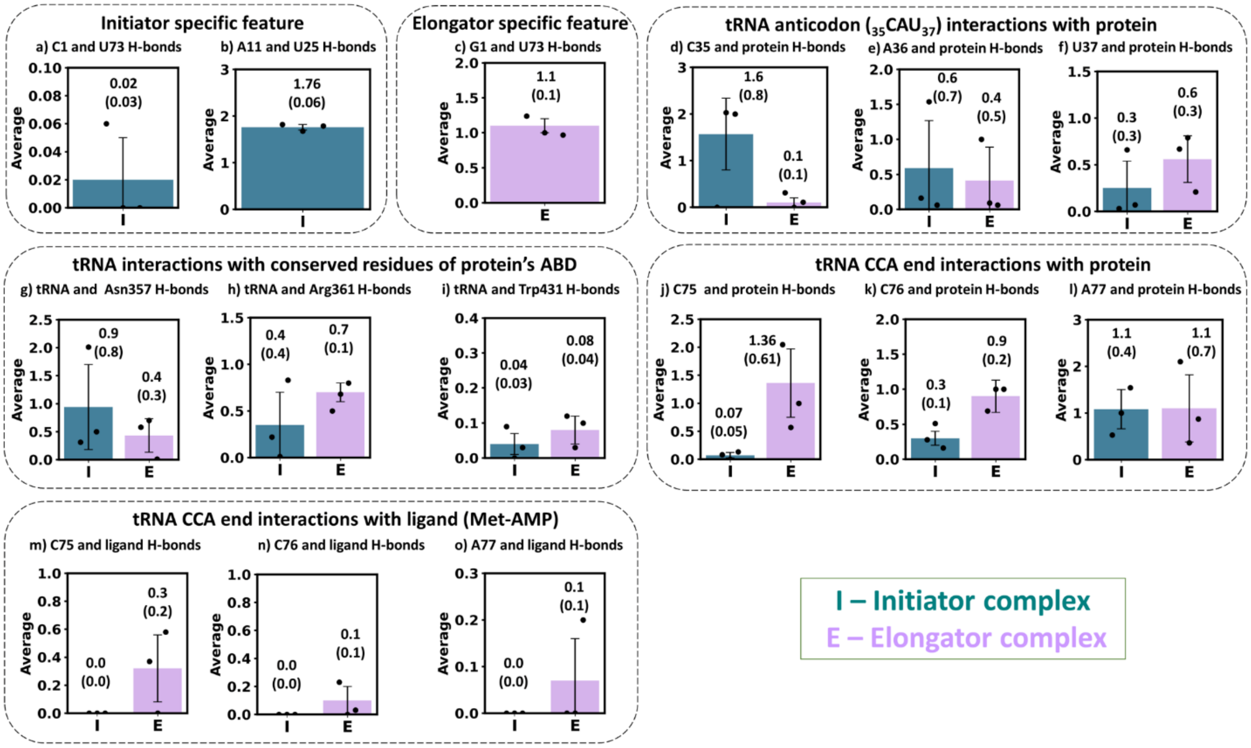

Figure S33. Average and standard value (value in bracket; error bar) comparison of H-bond counts for tRNA centric interactions. (a-b) Characteristic interactions of initiator tRNA, **(c)** characteristic interactions of elongator tRNA, **(d-f)** anticodon (CAU) interactions with protein residues, **(g-i)** tRNAs interactions with conserved protein residues (Asn357, Arg361, Trp431), **(j) - (l)** CCA end of tRNA interactions with protein residues and **(m-o)** CCA end of tRNA interactions with ligand.

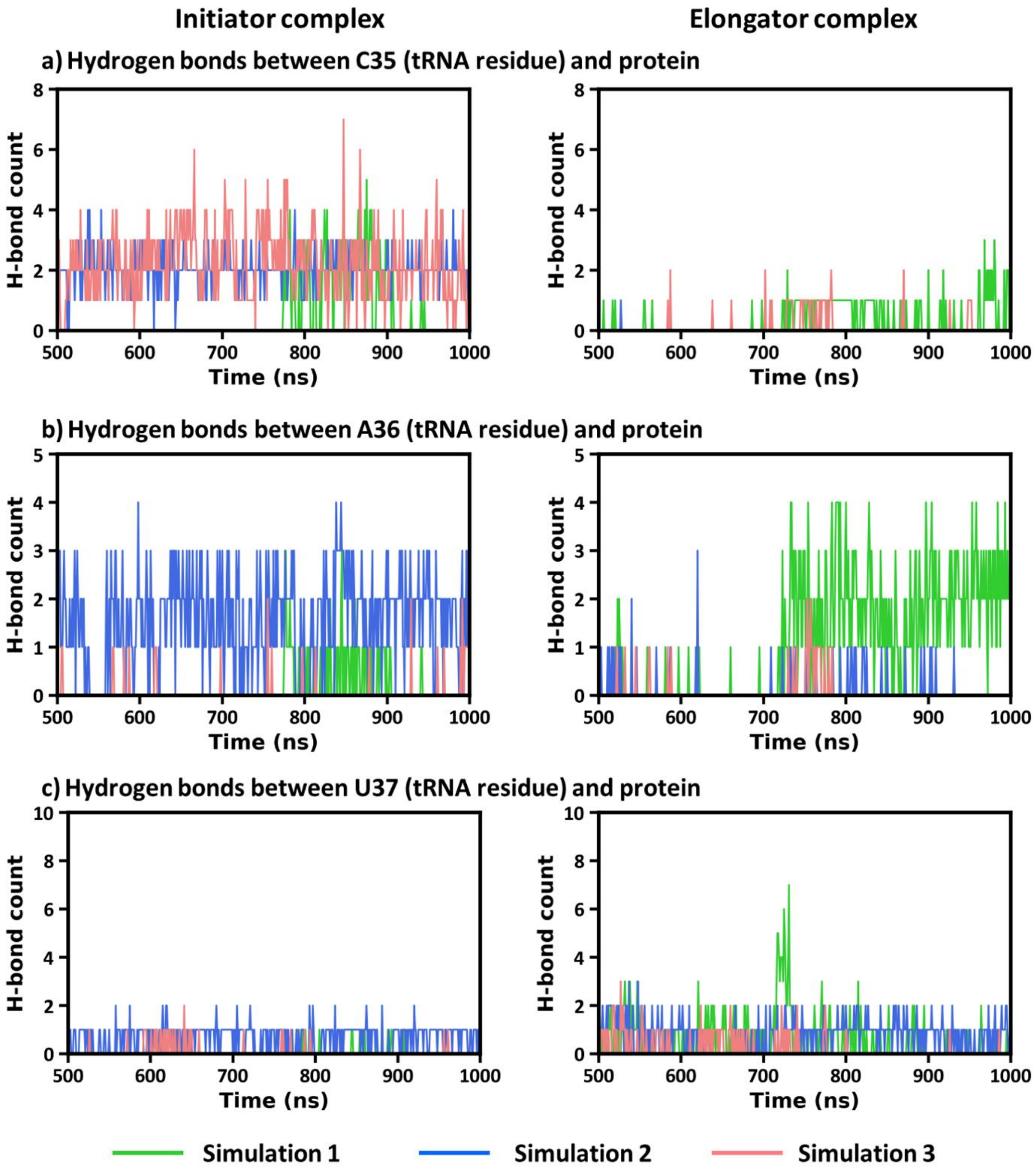

Figure S34. Hydrogen bonding interactions formed between CAU (35-37) anticodon and protein residues during the simulations. Interaction of **(a)** C35 nucleotide, **(b)** A36 nucleotide and **(c)** U37 nucleotide with protein.

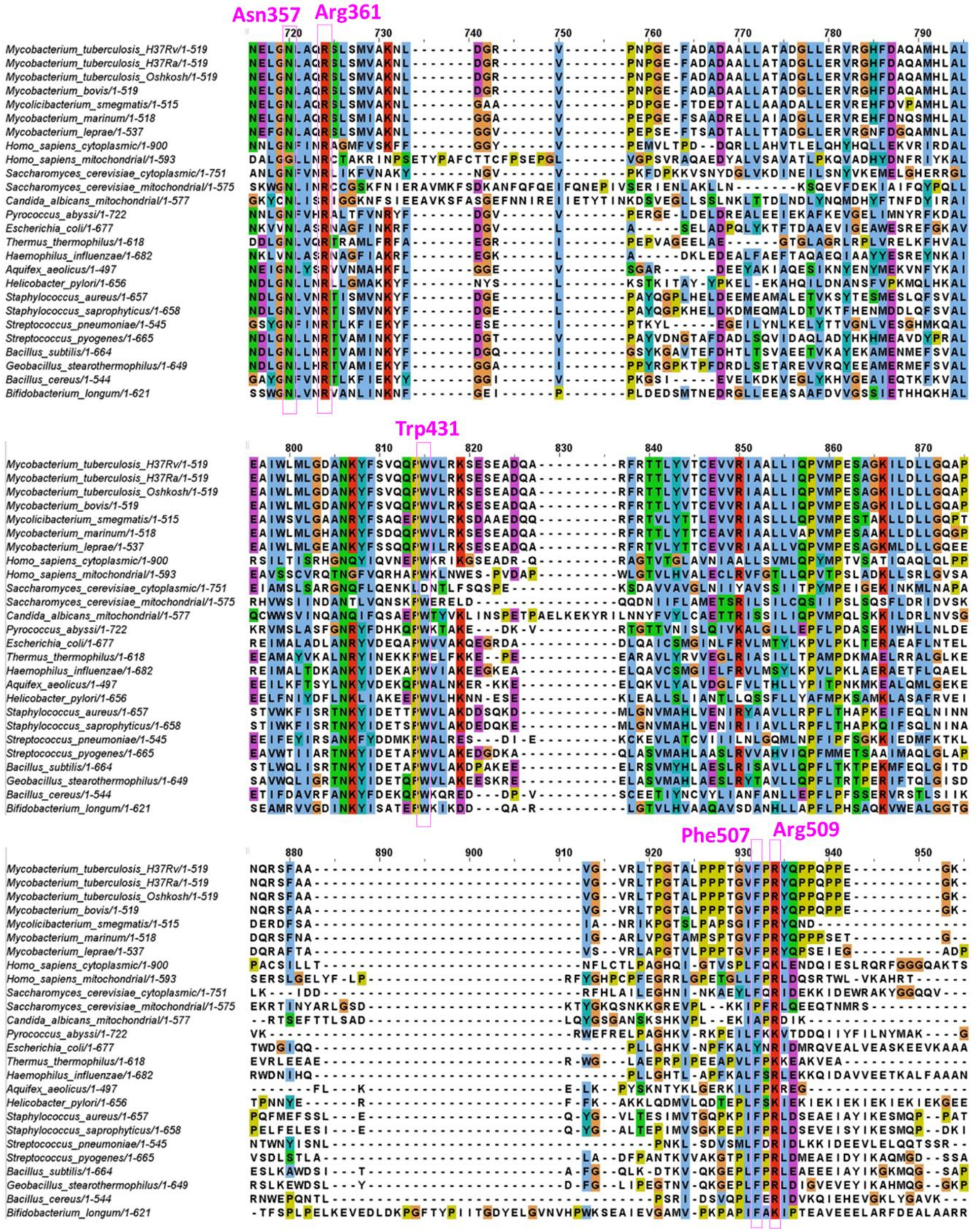

Figure S35. Conserved residues in anticodon domain of MetRS (shown in purple) from 26 different species.

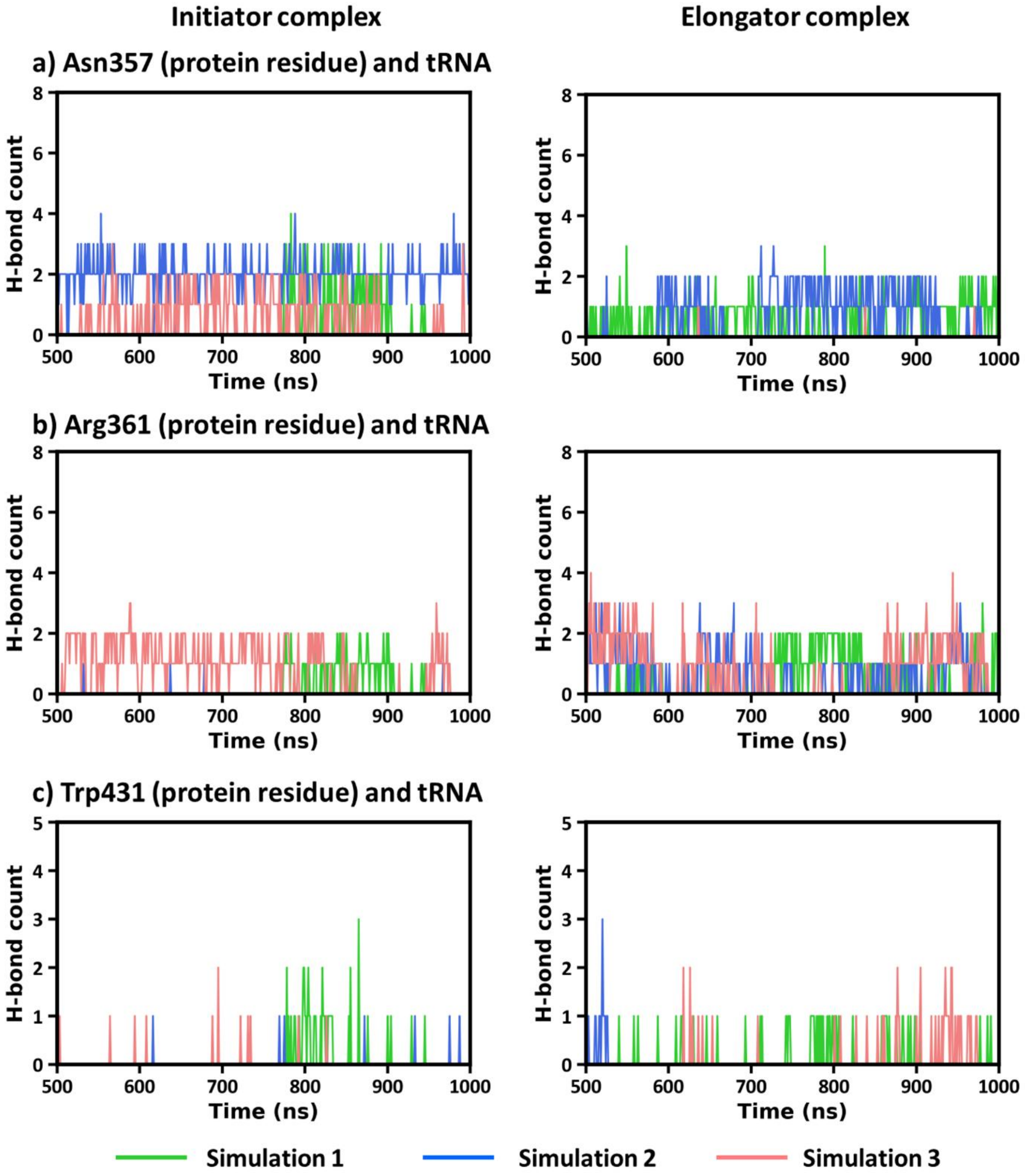

Figure S36. Hydrogen bonding between three conserved protein residues of anticodon domain and tRNA in initiator and elongator complexes. Hydrogen bond formed by **(a)** Asn357, **(b)** Arg361 and **(c)** Trp431 with tRNA.

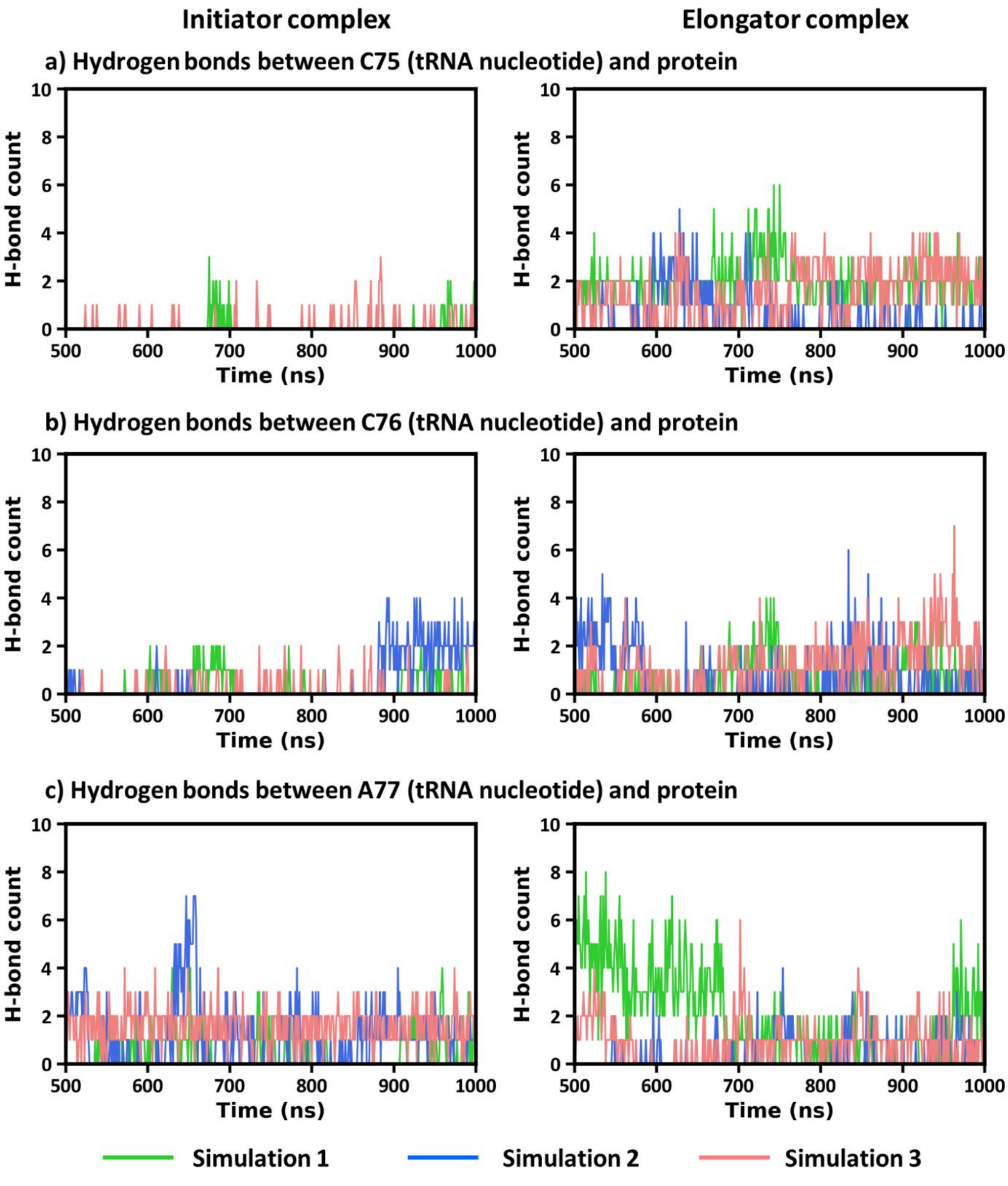

Figure S37. Hydrogen bond interactions formed by CCA (75-77) end of tRNA with protein residues. Interaction of **(a)** C75 nucleotide, **(b)** C76 nucleotide and **(c)** A77 nucleotide with protein.

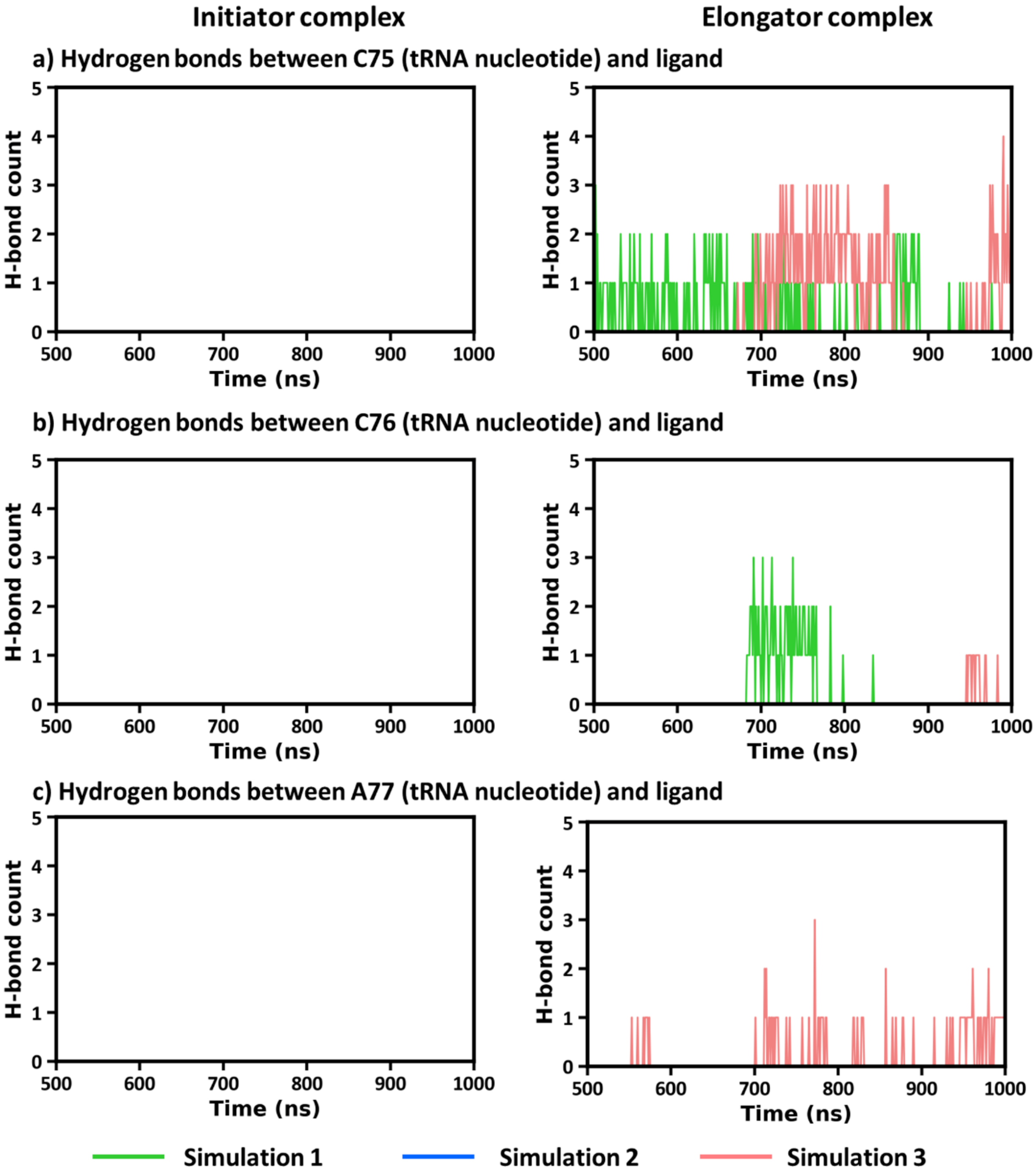

Figure S38. **Hydrogen bonding interactions formed by CCA end (75-77) with ligand (Met-AMP).** Interaction of **(a)** C75 nucleotide, **(b)** C76 nucleotide and **(c)** A77 nucleotide with ligand.

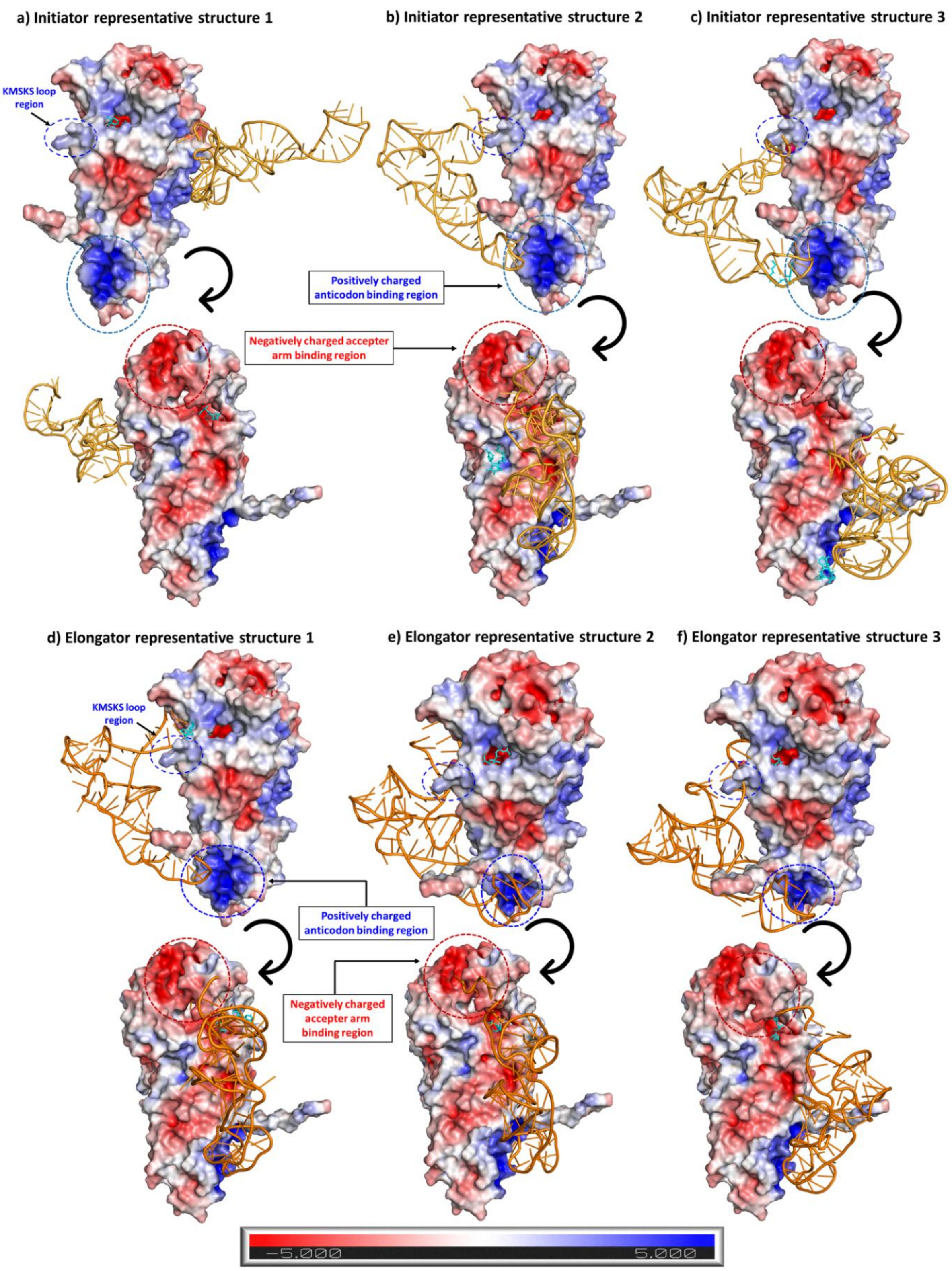

Figure S39. Electrostatic potential surface of representative structures. (a-c) initiator tRNA complex. **(d-f)** elongator tRNA complex.

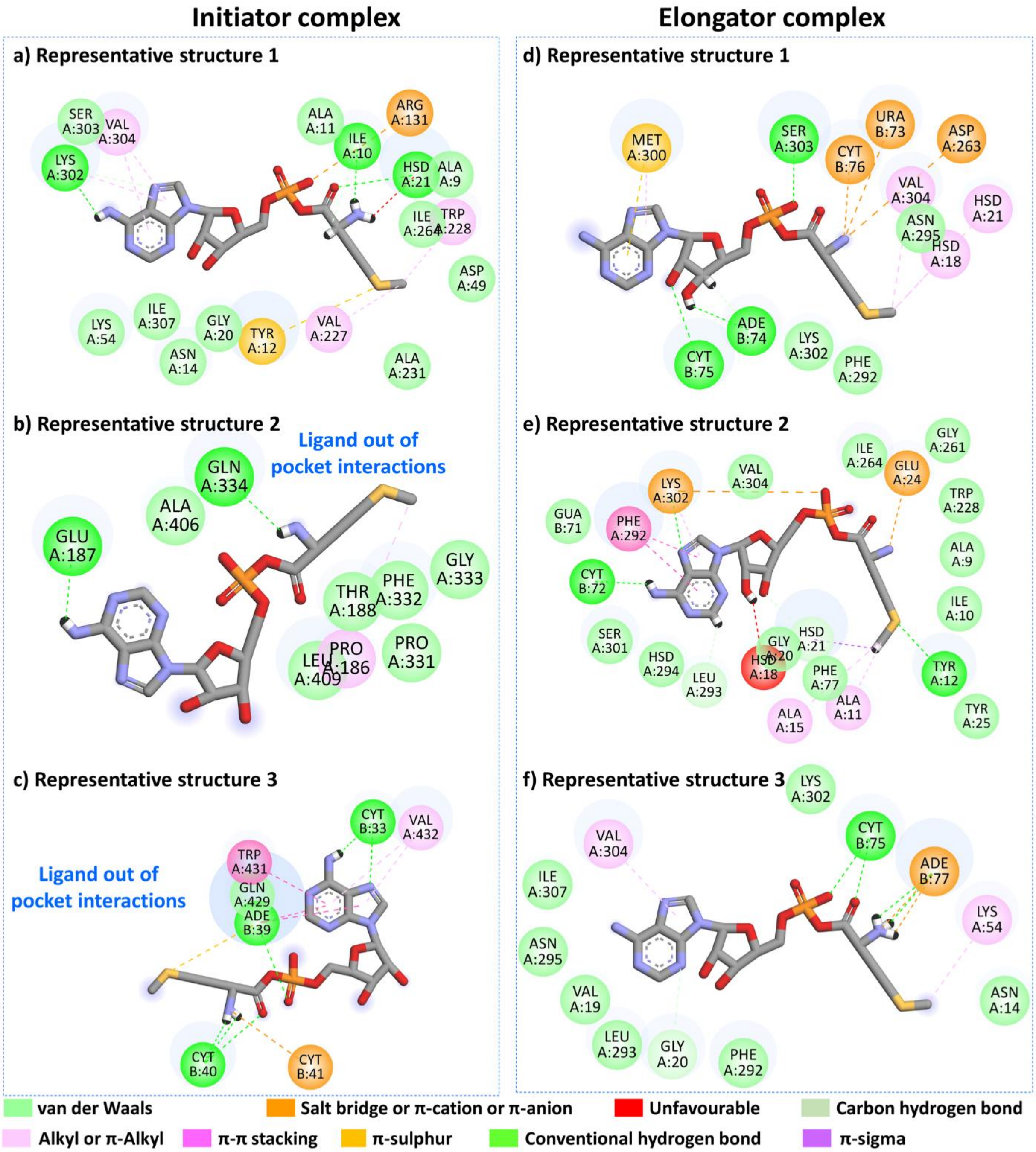

Figure S40. Interactions observed between protein residues, ligand and tRNA nucleotides in representative structures. (a-c) initiator and **(d-f)** elongator complex**.**

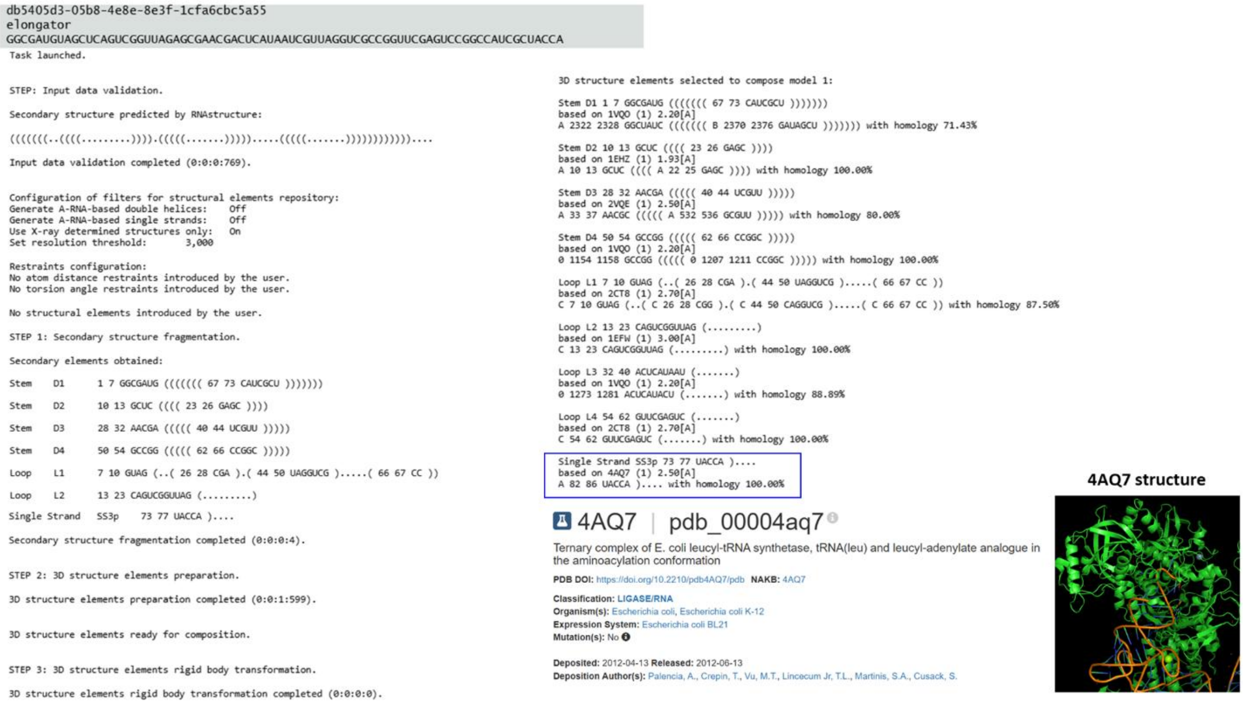

Figure S41. The details of templates used by RNAComposer to model elongator tRNA based on sequence. The 3′ CCA end of elongator tRNA was modelled using PDB – 4AQ7 (in blue box).

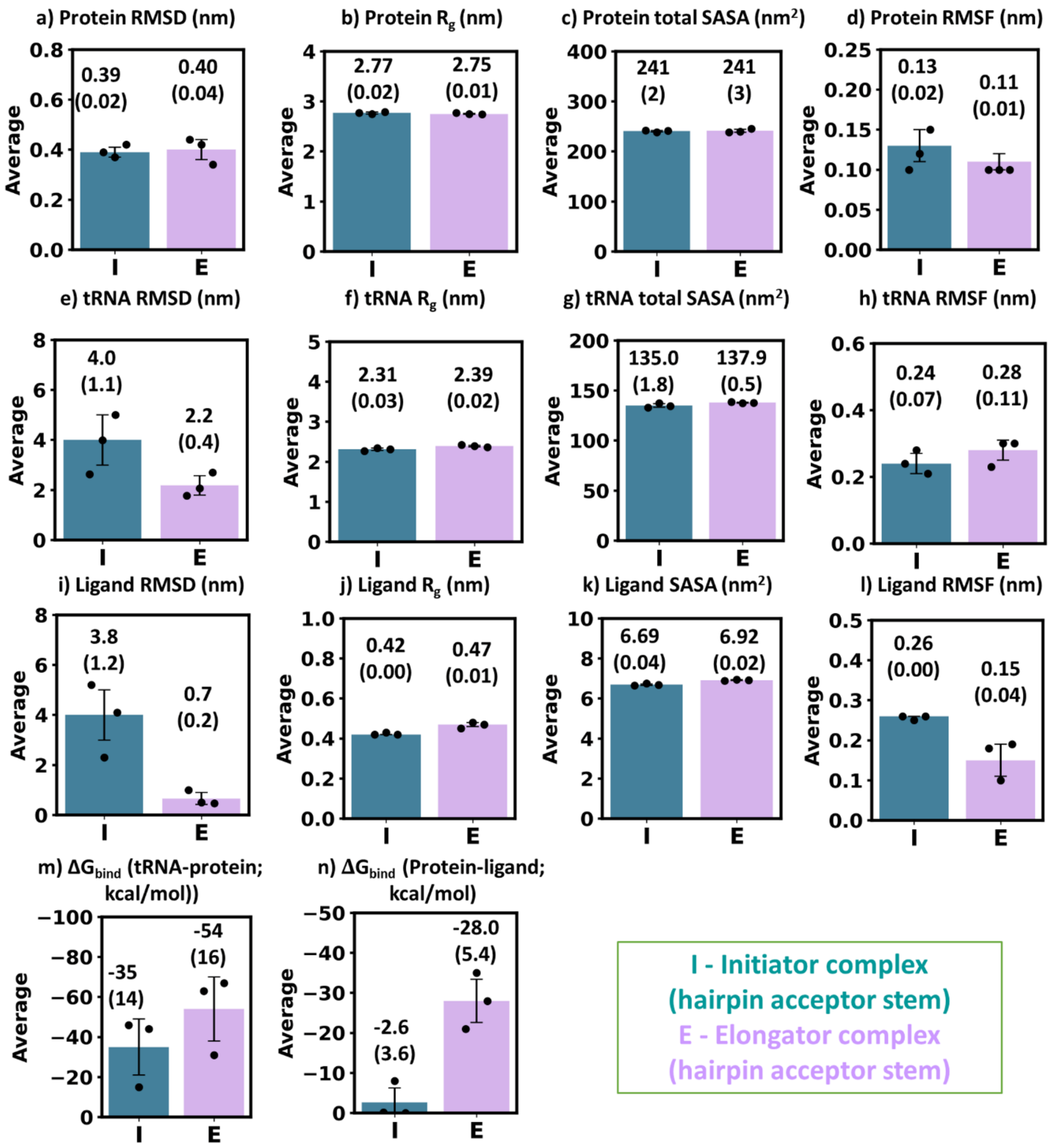

Figure S42. Comparisons of simulated average properties and standard deviations (value in bracket; error bar) from three independent MD runs for initiator (I) versus elongator (E) complexes. Protein properties **(a)** RMSD, **(b)** R_g, **(c)** SASA, and **(d)** RMSF. tRNA properties **(e)** RMSD, **(f)** R_g, **(g)** SASA, and **(h)** RMSF. Ligand (Met-AMP) properties **(i)** RMSD, **(j)** R_g, **(k)** SASA, and **(l)** RMSF. Binding free energy for **(m)** tRNA-protein and **(n)** protein-ligand interaction.

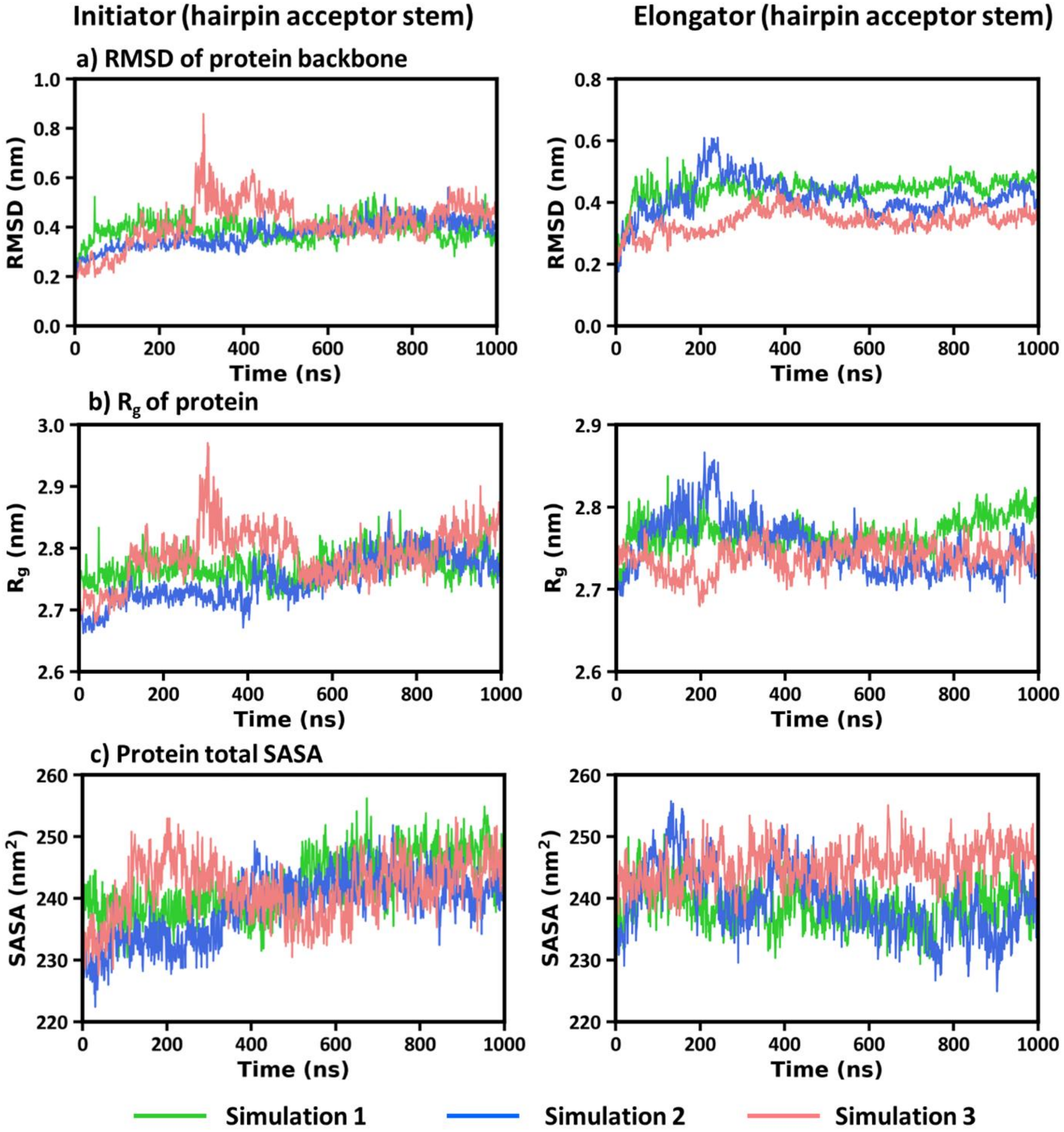

Figure S43. The simulated properties of MetRS protein. (a) The RMSD, **(b)** R_g, **(c)** total SASA of protein over three simulations (green, blue, peach).

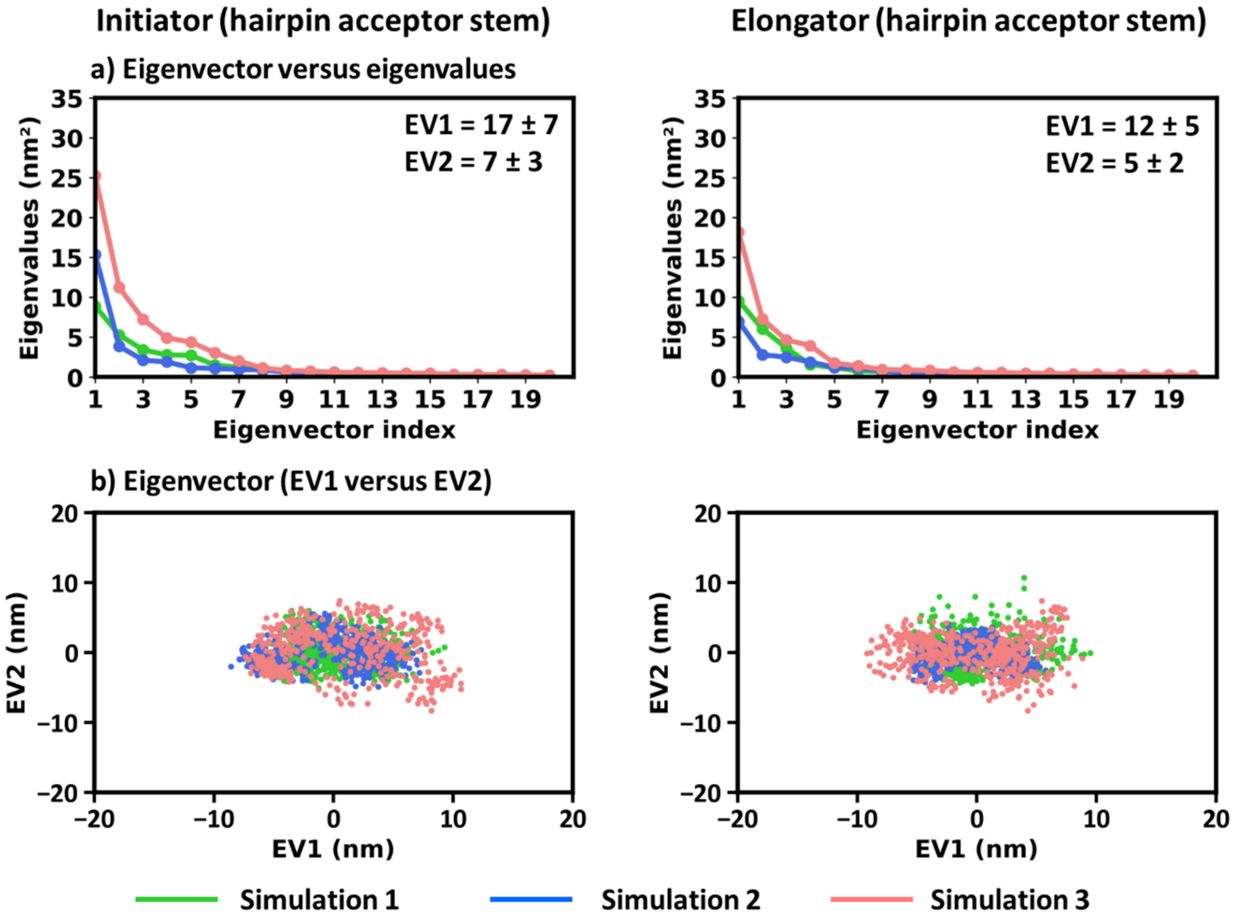

Figure S44. Free energy landscape principal component analysis (FEL-PCA) of protein. (a) First 20 eigenvectors (EV) with their eigenvalues. The average and standard deviation of eigenvalues for EV1 and EV2 over three simulations are provided. **(b)** EV1 versus EV2 projection plot for protein in initiator and elongator complex.

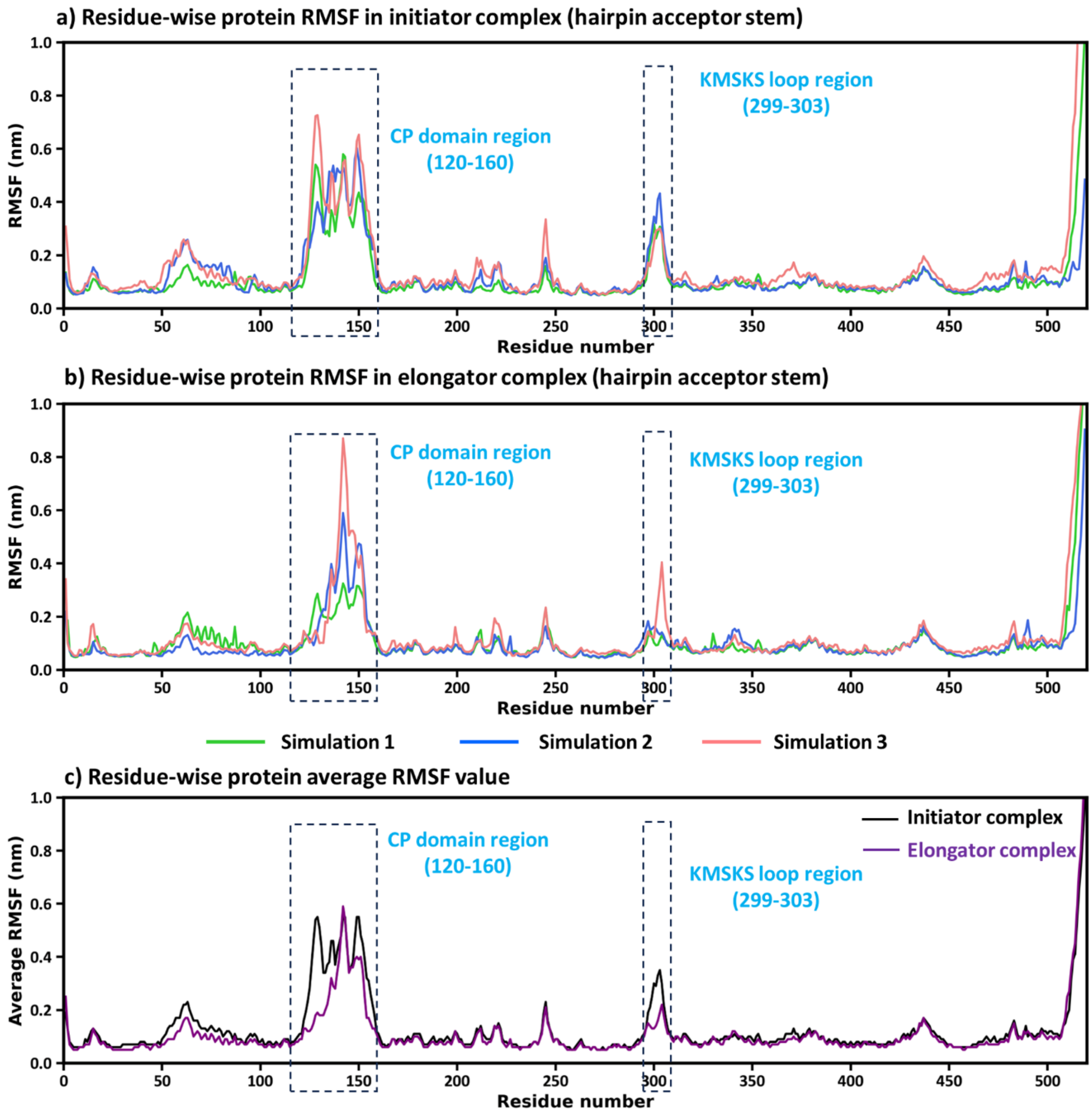

Figure S45. Residue-wise RMSF of MetRS protein in (a) initiator and **(b)** elongator tRNA-bound complexes across three simulations. **(c)** Average RMSF comparison highlights flexible regions, notably the CP domain (residues 120-160) and the KMSKS loop (299-303), showing differential mobility between complexes.

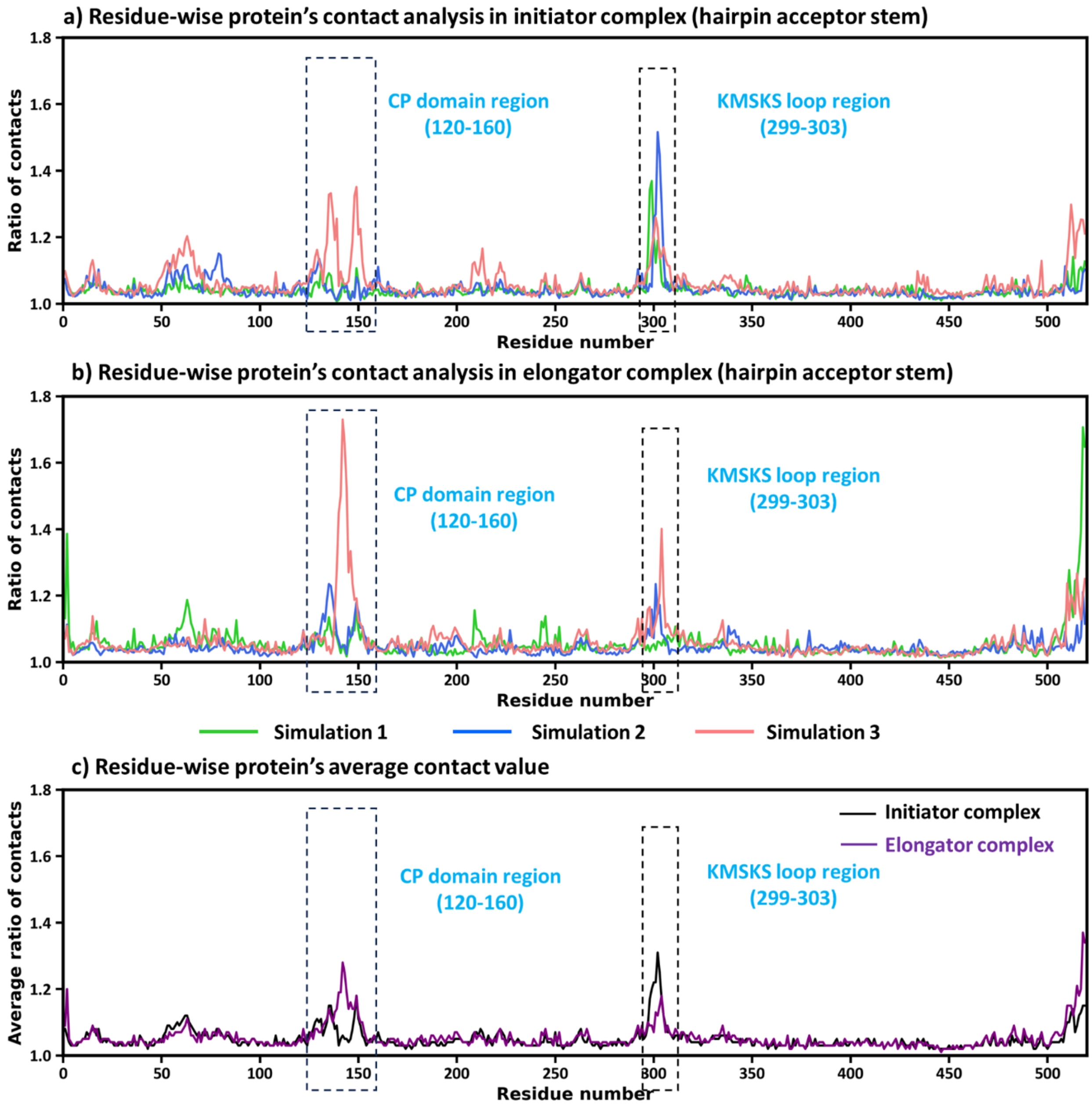

Figure S46. Residue-wise analysis of contacts in MetRS upon tRNA binding for (a) initiator and (b) elongator complexes over three simulations. Here "contacts" means count of number of different atomic contacts formed by each protein residue (x-axis) with atoms of other protein residues during simulations. "Ratio of contacts" is the total number of contacts divided by their mean value. **(c)** Average contact ratio comparison reveals enhanced contact formation in the CP domain (residues 120-160) and KMSKS loop (299-303).

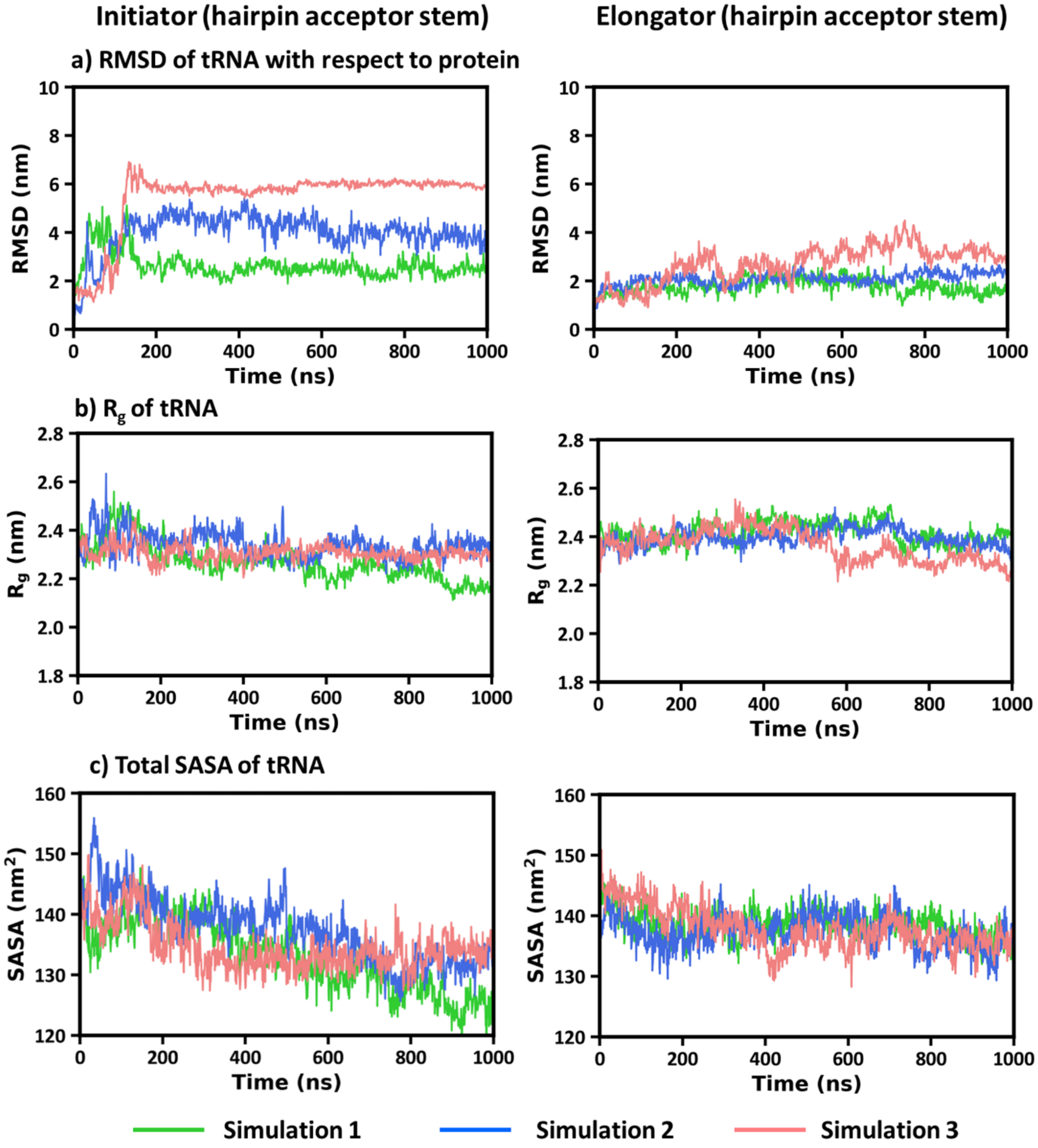

Figure S47. Comparisons of simulated properties of initiator and elongator tRNAs. (a) RMSD. **(b)** R_g. **(c)** Total SASA.

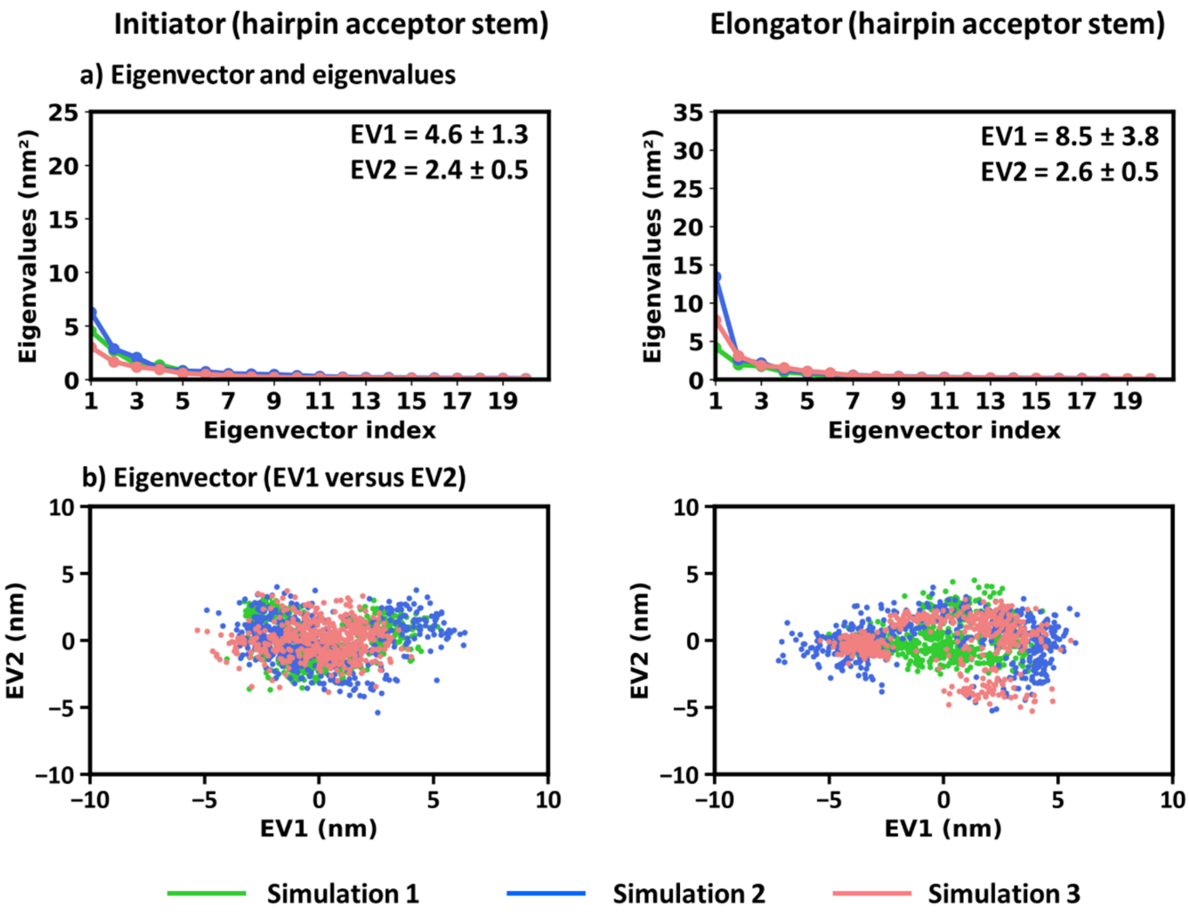

Figure S48. Principal component analysis of tRNA. (a) Eigenvectors versus eigenvalues plots. Average and standard deviation of eigenvalues for EV1 and EV2 over three simulations are provided. **(b)** 2D projections along EV1 and EV2.

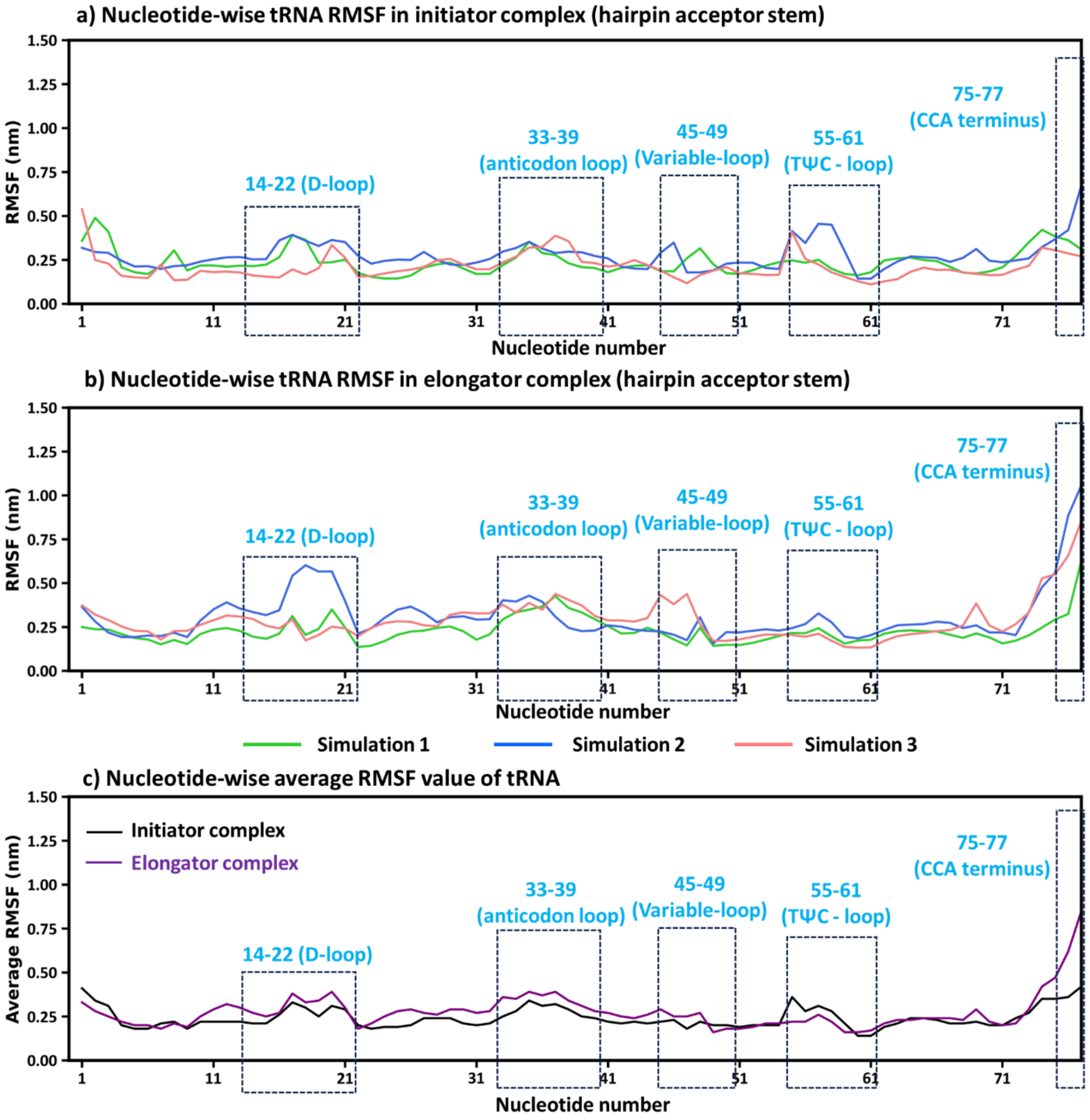

Figure S49. Nucleotide-wise RMSF analysis of tRNA in (a) initiator complex and **(b)** elongator complex. **(c)** Comparison of average RMSF values to show flexibility of nucleotides at the 5' end, D-loop (14-22), anticodon loop (33-39), variable loop (45-49), TΨC loop (55-61) and CCA terminus.

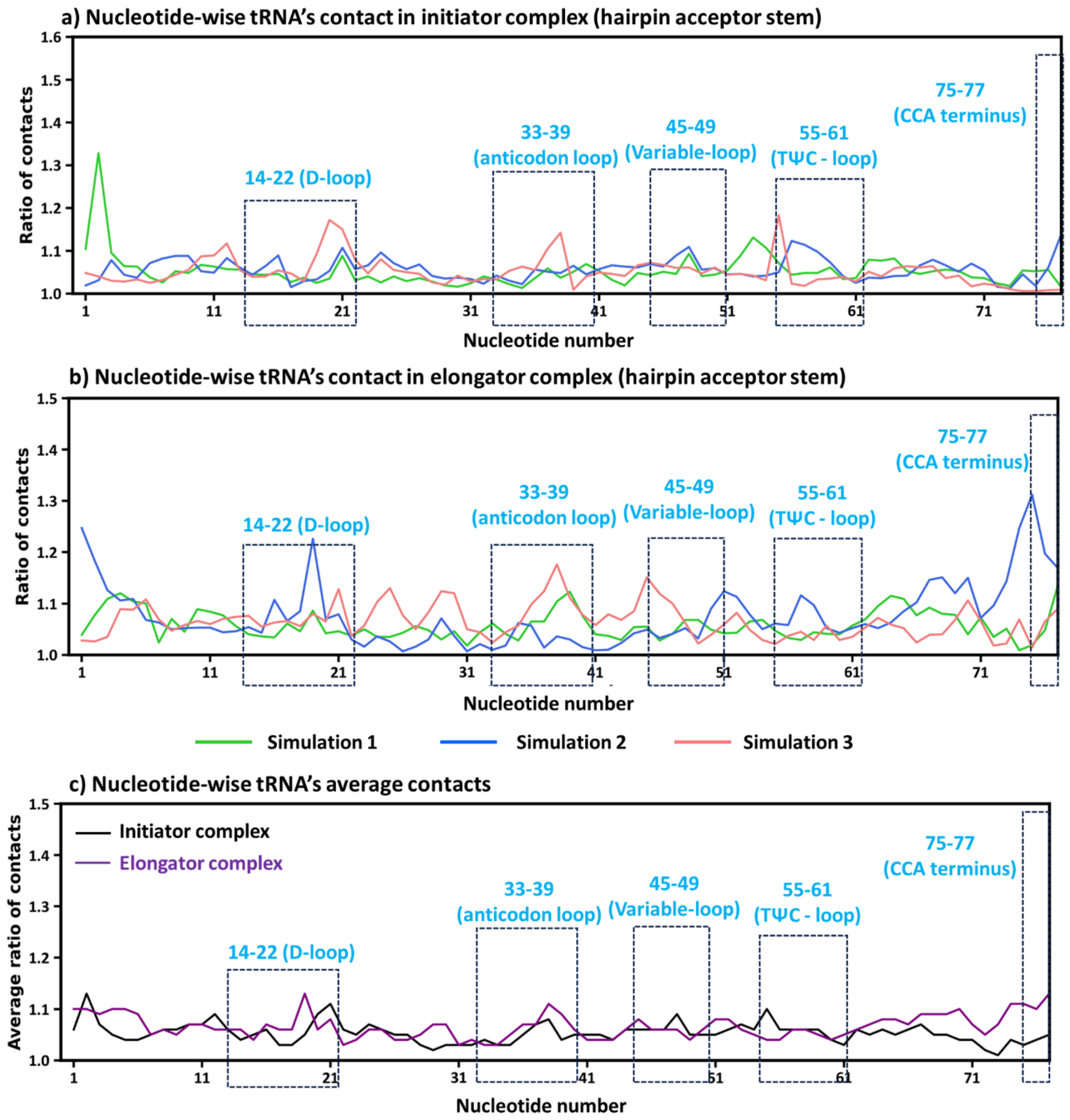

Figure S50. Nucleotide-wise contact analysis of tRNA in (a) initiator complex and **(b)** elongator complex. **(c)** Average contact ratio comparison reveals higher interaction dynamics in the initiator complex, especially at functionally important tRNA motifs. Here "contacts" means count of the number of different atomic contacts formed by each tRNA nucleotide (x-axis) with atoms of other nucleotides during simulations. "Ratio of contacts" is the total number of contacts divided by their mean value.

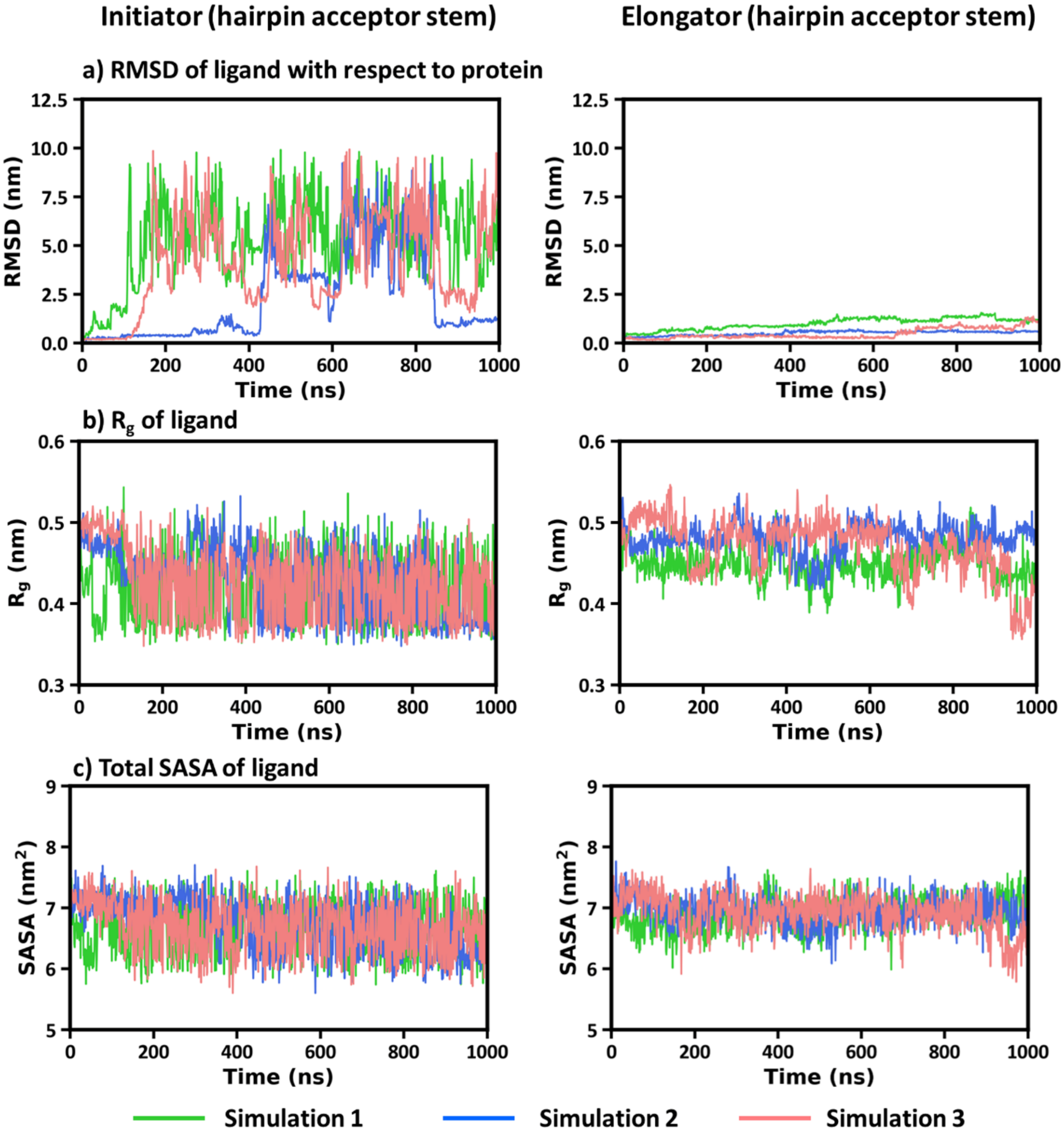

Figure S51. Structural properties of the ligand (Met-AMP) in the initiator and elongator complexes. **(a)** RMSD, **(b)** R_g, and **(c)** SASA of ligand.

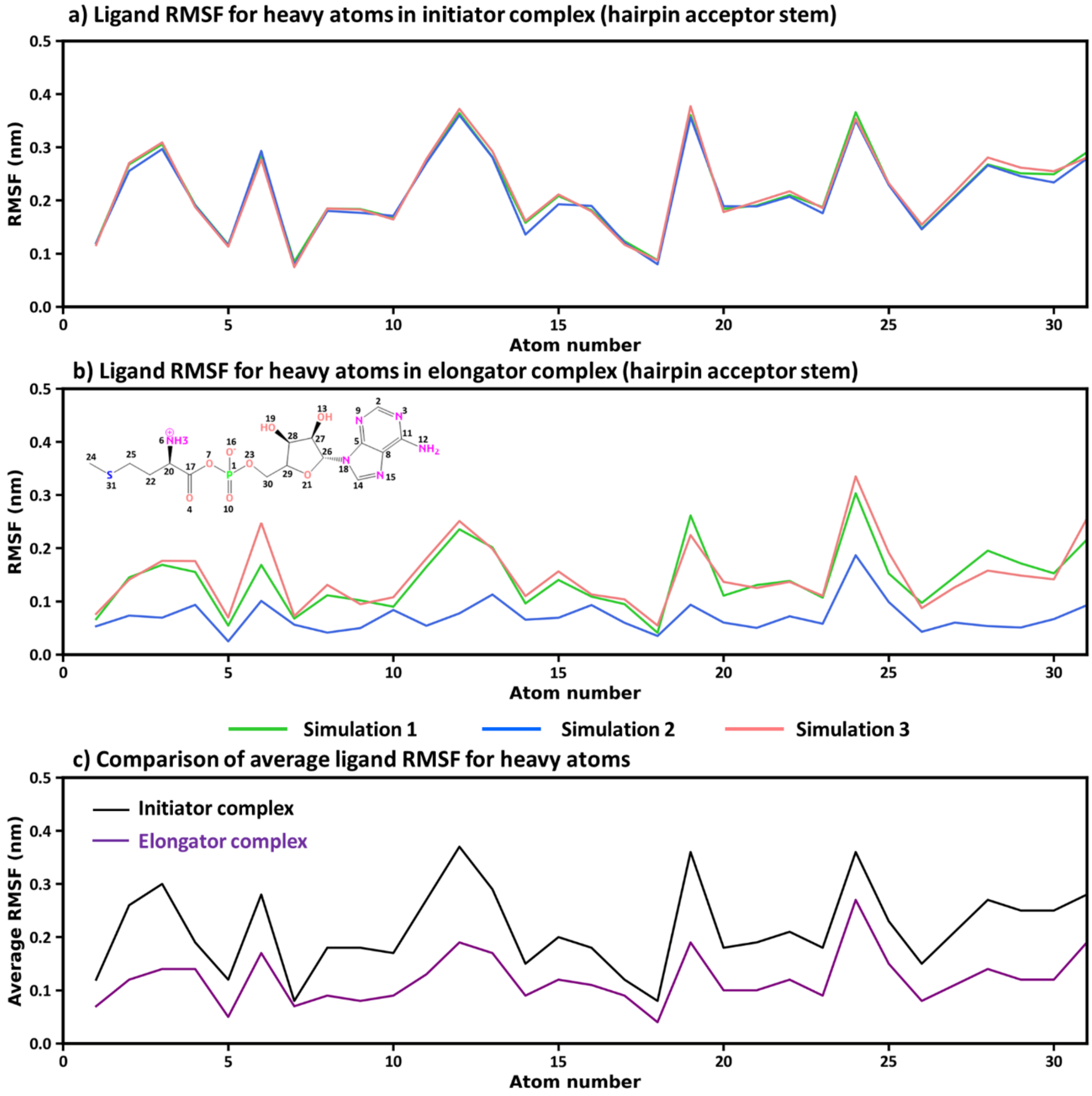

Figure S52. RMSF analysis of heavy atoms in the ligand molecule. (a) and **(b)** depict the heavy atom RMSF of ligand in the initiator and elongator complexes, respectively. **(c)** Comparison of average RMSF values confirms reduced ligand mobility in the elongator complex.

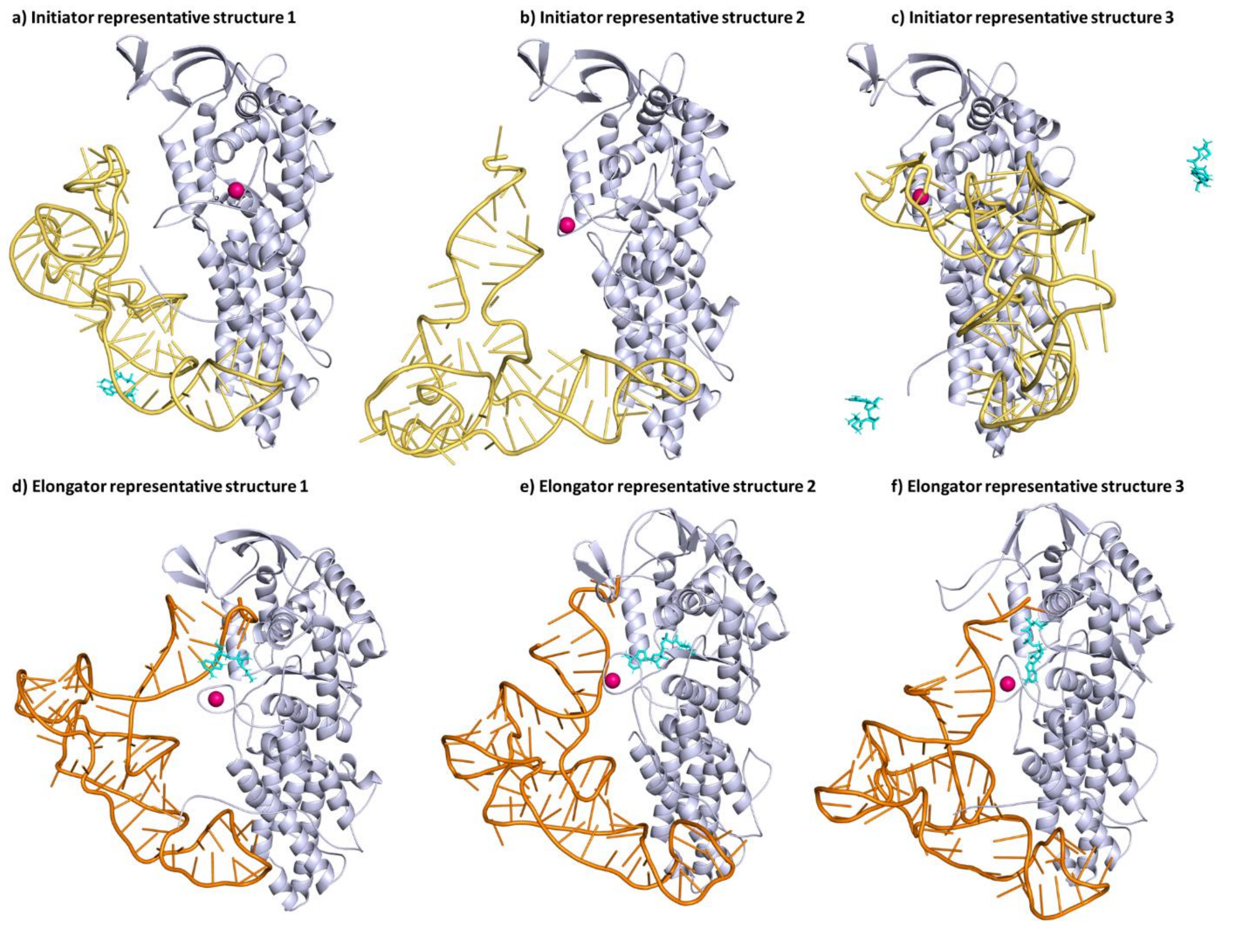

Figure S53. Representative structures from each simulation run of initiator and elongator tRNA. (a-c) initiator tRNA complex (hairpin acceptor stem). **(d-f)** elongator tRNA complex (hairpin acceptor stem).

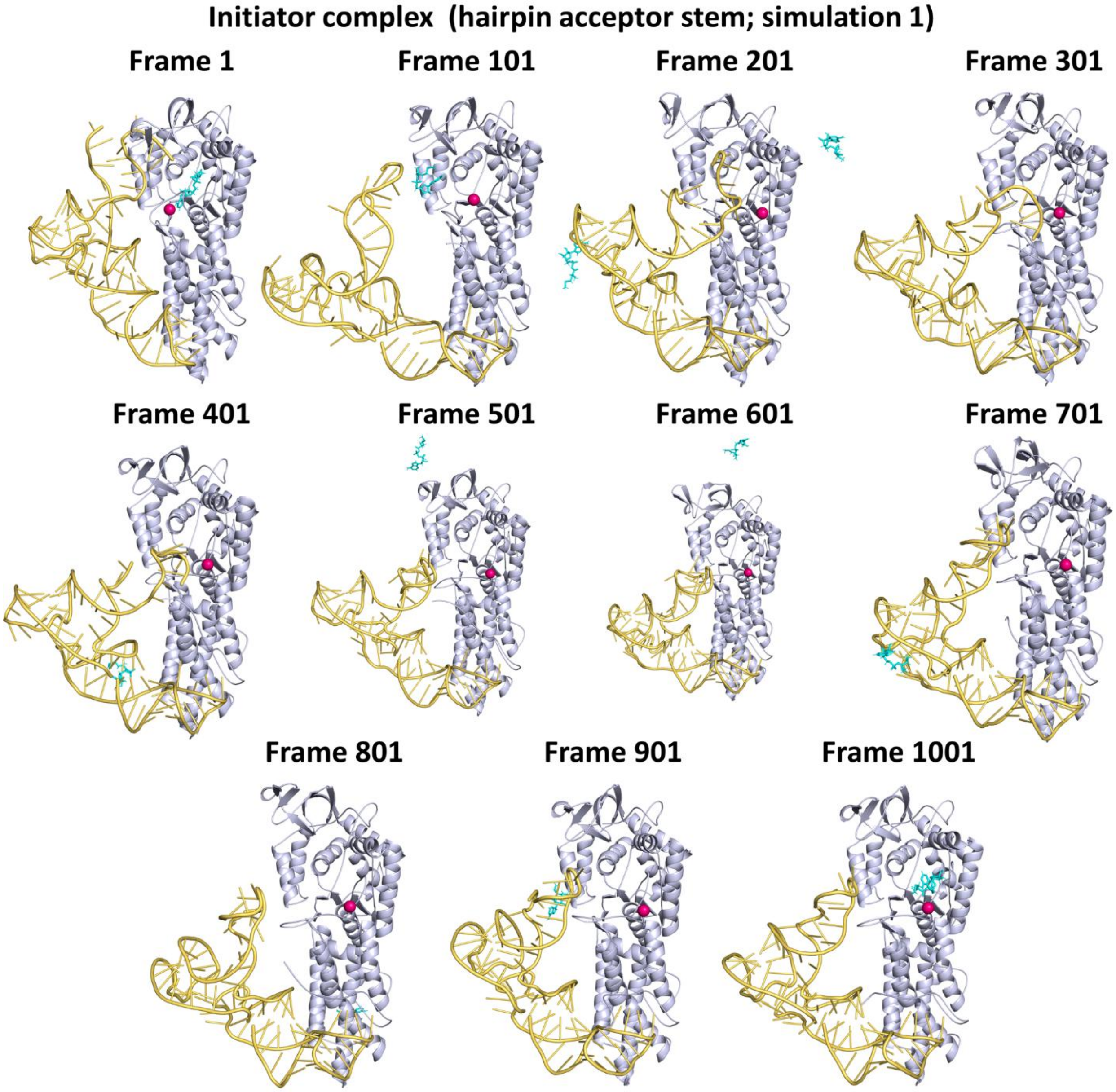

Figure S54. Trajectory snapshots from simulation 1 of the initiator complex (hairpin acceptor stem). Frame 1 corresponds to 0 ns, frame 101 to 100 ns, and at the end frame 1001 to 1000 ns.

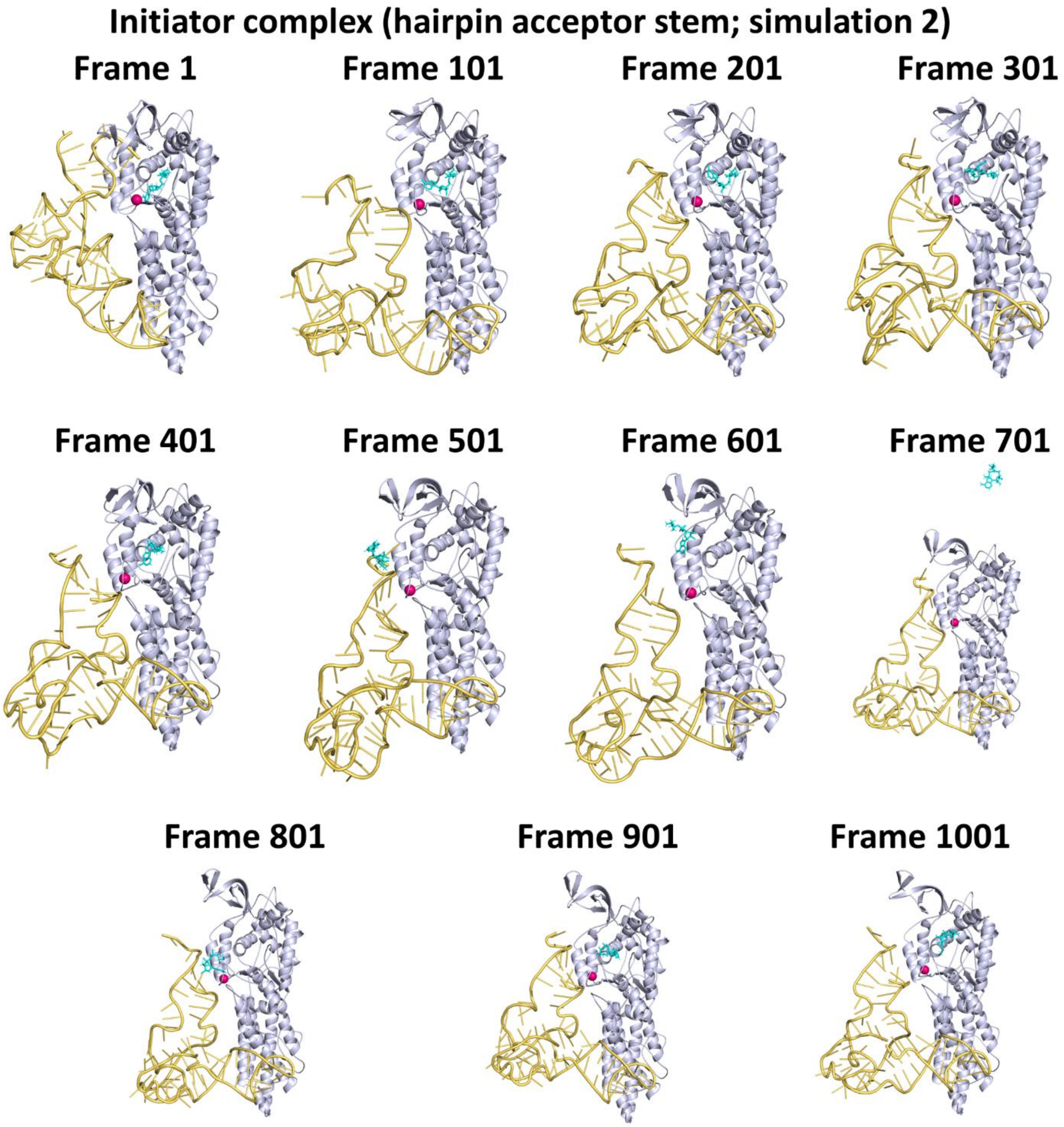

Figure S55. Trajectory snapshots from simulation 2 of the initiator complex (hairpin acceptor stem). Frame 1 corresponds to 0 ns, frame 101 to 100 ns, and at the end frame 1001 to 1000 ns.

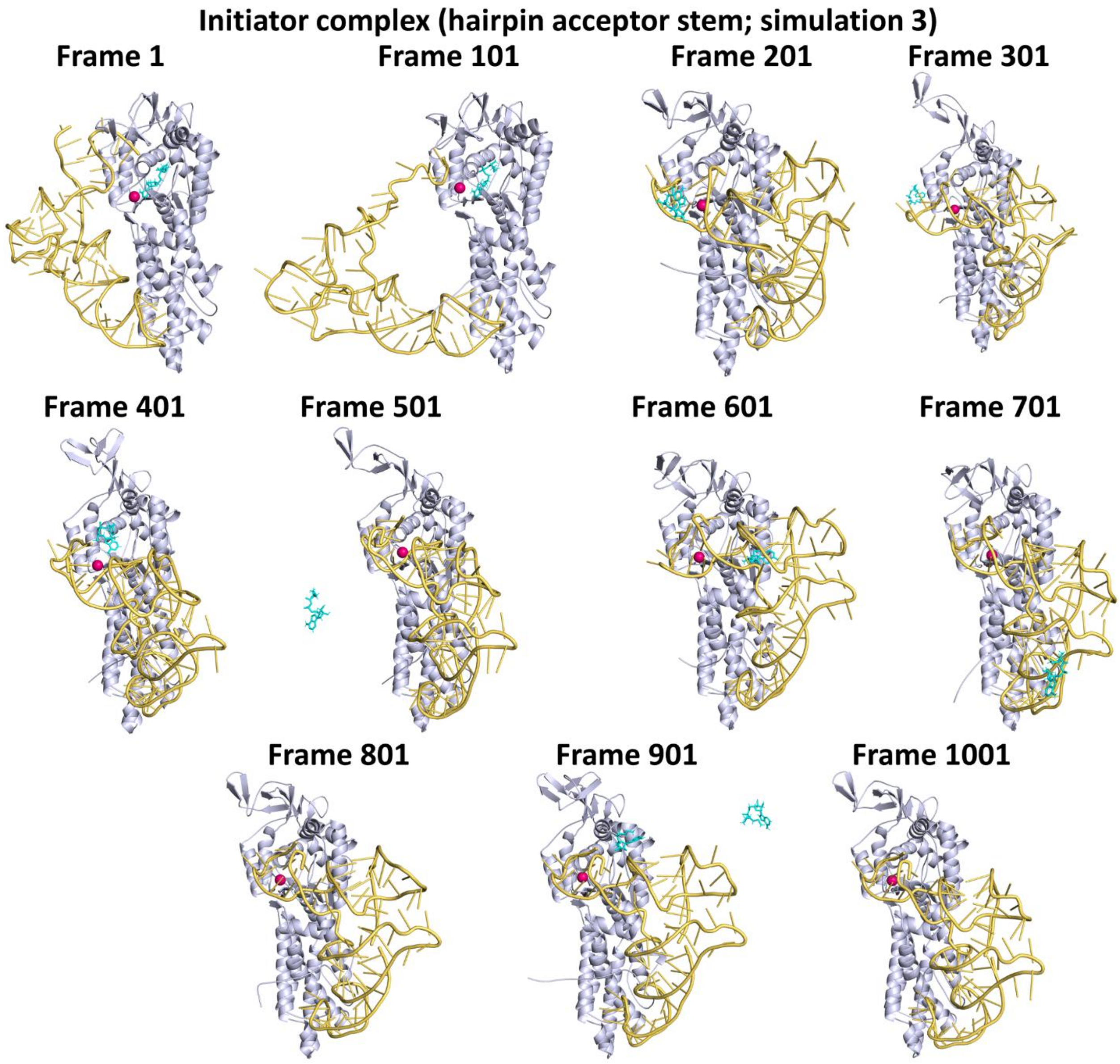

Figure S56. Trajectory snapshots from simulation 3 of the initiator complex (hairpin acceptor stem). Frame 1 corresponds to 0 ns, frame 101 to 100 ns, and at the end frame 1001 to 1000 ns.
